# Where’s Waldo? How perceptual, cognitive, and emotional brain processes cooperate during learning to categorize and find desired objects in a cluttered scene

**DOI:** 10.3389/fnint.2014.00043

**Published:** 2014-06-17

**Authors:** Hung-Cheng Chang, Stephen Grossberg, Yongqiang Cao

**Affiliations:** Graduate Program in Cognitive and Neural Systems, Department of Mathematics, Center for Adaptive Systems, Center for Computational Neuroscience and Neural Technology, Boston UniversityBoston, MA, USA

**Keywords:** visual search, Where’s Waldo problem, spatial attention, object attention, category learning, gain field, reinforcement learning, eye movement

## Abstract

The Where’s Waldo problem concerns how individuals can rapidly learn to search a scene to detect, attend, recognize, and look at a valued target object in it. This article develops the ARTSCAN Search neural model to clarify how brain mechanisms across the What and Where cortical streams are coordinated to solve the Where’s Waldo problem. The What stream learns positionally-invariant object representations, whereas the Where stream controls positionally-selective spatial and action representations. The model overcomes deficiencies of these computationally complementary properties through What and Where stream interactions. Where stream processes of spatial attention and predictive eye movement control modulate What stream processes whereby multiple view- and positionally-specific object categories are learned and associatively linked to view- and positionally-invariant object categories through bottom-up and attentive top-down interactions. Gain fields control the coordinate transformations that enable spatial attention and predictive eye movements to carry out this role. What stream cognitive-emotional learning processes enable the focusing of motivated attention upon the invariant object categories of desired objects. What stream cognitive names or motivational drives can prime a view- and positionally-invariant object category of a desired target object. A volitional signal can convert these primes into top-down activations that can, in turn, prime What stream view- and positionally-specific categories. When it also receives bottom-up activation from a target, such a positionally-specific category can cause an attentional shift in the Where stream to the positional representation of the target, and an eye movement can then be elicited to foveate it. These processes describe interactions among brain regions that include visual cortex, parietal cortex, inferotemporal cortex, prefrontal cortex (PFC), amygdala, basal ganglia (BG), and superior colliculus (SC).

## 1. Introduction

**Figure 1:**
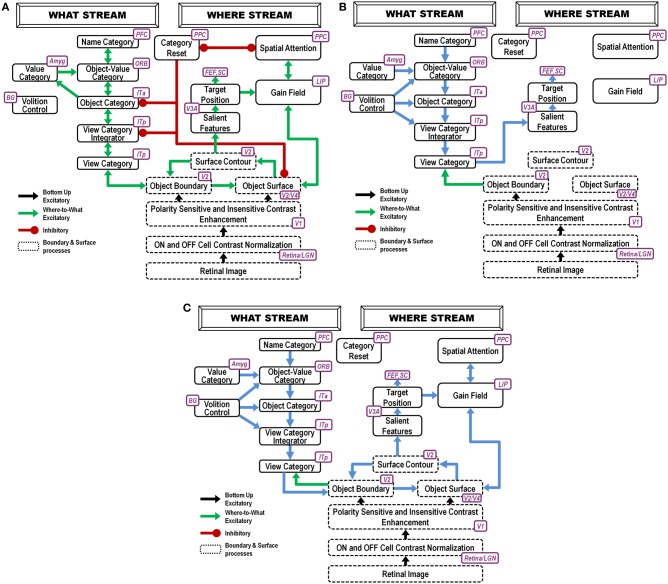
**ARTSCAN Search diagram**. The dashed boxes indicate boundary and surface processes. **(A)** Category learning. The arrows represent the excitatory cortical processes from Where cortical stream to What cortical stream whereby invariant category learning and recognition, and reinforcement learning, occur. The connections ending in circular disks indicate inhibitory connections. **(B)** Direct pathway of top-down primed search from the What to the Where cortical stream. **(C)** Indirect pathway of top-down primed search from the What to the Where cortical stream. In both **(B)** and **(C)**, the green arrows represent bottom-up image-driven processes and the blue arrows represent top-down processes from What cortical stream to Where cortical stream. See Figures 5, 6 and surrounding text for more details about the temporal progression of top-down searches. ITa, anterior part of inferotemporal cortex; ITp, posterior part of inferotemporal cortex; PPC, posterior parietal cortex; LIP, lateral intra-parietal cortex; LGN, lateral geniculate nucleus; ORB, orbitofrontal cortex; Amyg, amygdala; BG, basal ganglia; PFC, prefrontal cortex; SC, superior colliculus; V1 and V2, primary and secondary visual areas; V3 and V4, visual areas 3 and 4.

This paper develops a neural model, called the ARTSCAN Search model (Figure 1), to explain how the brain solves the Where’s Waldo problem; in particular, how individuals can rapidly search a scene to detect, attend, recognize and look at a target object in it. The model predicts how the brain overcomes the deficiencies of computationally complementary properties of the brain’s What and Where cortical processing streams. The ventral What stream is associated with object learning, recognition, and prediction, whereas the dorsal Where stream carries out processes such as object localization, spatial attention, and eye movement control (Ungerleider and Mishkin, 1982; Mishkin et al., 1983; Goodale and Milner, 1992). To achieve efficient object recognition, the What stream learns object category representations that are increasingly invariant under view, position, and size changes at higher processing stages. Such invariance enables objects to be learned and recognized without causing a combinatorial explosion. However, by stripping away the positional coordinates of each object exemplar, the What stream loses the ability to command actions to the positions of valued objects. The Where stream computes positional representations of the world and controls actions to acquire objects in it, but does not represent detailed properties of the objects themselves. The ARTSCAN Search model shows how What stream properties of positionally-invariant recognition and Where stream properties of positionally-selective search and action can interact to achieve Where’s Waldo searches.

The model’s Where cortical stream processes of spatial attention and predictive eye movement control modulate What cortical stream processes whereby multiple view- and positionally-specific object categories are learned and associatively linked to view- and positionally-invariant object categories through both bottom-up and attentive top-down interactions. Gain fields control retinotopic and head-center coordinate transformations that enable spatial attention and predictive eye movements to carry out this role. In addition, What stream cognitive-emotional learning processes enable the focusing of motivated attention upon the invariant object categories of desired objects.

To carry out a goal-directed search, the model can use either a cognitive name or motivational drive to prime a view- and positionally-invariant object category representation in its What cortical stream. A major design challenge for the model is to show how priming of such a positionally-invariant category can drive a search that finds Waldo at a particular position. In particular, how does a positionally-invariant representation in the What stream shift spatial attention in the Where stream to a representation of Waldo’s position and activate an eye movement to foveate that position?

This is proposed to happen as follows: A volitional signal can convert the prime of the invariant object category into suprathreshold activation of the category. Once activated, the invariant category can, in turn, prime What stream view- and positionally-selective categories. When combined with bottom-up activation by the desired target of the positionally-selective category that represents the target’s identity and position, this positionally-selective category can achieve suprathreshold activation. It can then cause spatial attention to shift in the Where stream to a representation of the target’s position, after which an eye movement can be elicited to acquire it.

As illustrated in Figure 1, these processes are assumed to occur in the model analogs of the following brain regions: Spatial attention is carried out in the posterior parietal cortex (PPC). The view- and positionally-selective categories are learned in the posterior inferotemporal cortex (ITp). View- and positionally-invariant categories are learned in the anterior inferotemporal cortex (ITa). The cognitive priming by names arises in the prefrontal cortex (PFC), whereas motivational priming arises in the amygdala (AMYG) and activates object-value categories in the orbitofrontal cortex (ORB). The volitional signals arise in the basal ganglia (BG). The selection and control of eye movements includes cortical area V3A, the frontal eye fields (FEF), and the superior colliculus (SC). The gain fields within the lateral interparietal cortex (LIP) are activated by V3A and mediate between PPC and visual cortical areas that include V4. Preprocessing of visual boundary and surface representations occurs in the retina and lateral geniculate nucleus (LGN) and cortical areas V1, V2, and V4. More detailed explanations are provided below. The model has been briefly reported in Chang et al. (2009a,b, 2013).

This theoretical synthesis unifies and extends several previous neural models, notably the ARTSCAN model of view-invariant object category learning (Grossberg, 2007, 2009; Fazl et al., 2009; Foley et al., 2012), its extension to the positionally-invariant ARTSCAN, or pARTSCAN, model of view-, position-, and size-invariant object category learning (Cao et al., 2011), and the CogEM (Cognitive-Emotional-Motor) model of cognitive-emotional learning and motivated attention (Grossberg, 1971, 1972a,b, 1975, 1982, 1984; Grossberg and Levine, 1987; Grossberg and Schmajuk, 1987; Grossberg and Seidman, 2006; Dranias et al., 2008; Grossberg et al., 2008). pARTSCAN’s ability to recognize objects in multiple positions is needed as part of the Where’s Waldo search process. In particular, name or motivational primes can then, supplemented by a volitional signal, activate an object-value category and, from there, an object category that has view- and positionally-invariant properties. Such cognitive-emotional and motivated attention processes are modeled in the CogEM model, which is joined with pARTSCAN to enable motivationally-primed searches in the ARTSCAN Search model.

All of these component models have quantitatively explained and predicted large psychological and neurobiological databases. Some of these explanations are reviewed below. ARTSCAN Search preserves these previously demonstrated explanatory and predictive capabilities, while also making novel predictions.

During a Where’s Waldo search, when the positionally-invariant category is activated in the What stream, it needs to be able to activate, through top-down learned connections, its corresponding view- and positionally-selective categories in the What stream. The pARTSCAN model included only bottom-up learned links from view- and positionally-selective category representations in ITp to view- and positionally-invariant category representations in ITa, and then to naming categories in PFC. The ARTSCAN Search model incorporates, in addition, reciprocal top-down learned links from PFC to ITa, and from the invariant ITa categories to the variant ITp categories (Figures 1B,C).

Such reciprocal links are a part of Adaptive Resonance Theory, or ART, learning dynamics whereby invariant recognition categories and their naming categories are learned. As explained by ART (Grossberg, 1980b, 2012; Carpenter and Grossberg, 1991), these top-down links dynamically stabilize category learning against catastrophic forgetting. With all these top-down learned links in place, activating a name for the desired goal object can activate the corresponding positionally-invariant category representation, which in turn can attentively prime all the positionally-selective categories where the sought-after target object may be. When one of the primed positionally-selective categories is also activated bottom-up by the sought-after object, that category can fire, and can thereby activate the corresponding positional representation in PPC (Figures 1B,C). This What-to-Where stream interaction can draw spatial attention to the position of the desired target, which in turn can activate an eye movement to foveate the target before further engaging it. In addition to these top-down connections, volition control signals from the BG (Figures 1B,C), which were also not part of the pARTSCAN model, ensure that the appropriate top-down connections can fully activate, rather than just subliminally prime, their target cells (Figures 1B,C).

The ARTSCAN Search model hereby incorporates both cognitive-emotional and cognitive-perceptual bi-directional interactions between cortical streams to achieve both Where-to-What invariant object category learning and What-to-Where primed search for a desired object.

Sections 2 and 3 summarize how the ARTSCAN model embodies solutions to three important design problems in order to learn view-invariant object categories: the view-to-binding problem, the coordination of spatial attention and visual search, and the complementary interactions that occur between spatial attention and object attention. Section 3 summarizes how the ARTSCAN model regulates spatial attention using predictive remapping, surface contour signals, and eye movement search. Section 4 summarizes how the pARTSCAN model enables learning of object categories that are view-invariant and positionally-invariant. They are also size-invariant, but that is not a focus of the present study. Section 5 describes how CogEM cognitive-emotional interactions regulate reinforcement learning and motivated attention. Section 6 describes how top-down primed cognitive and motivational searches are incorporated into the ARTSCAN Search model via What-to-Where stream interactions, including the top-down learned cognitive and motivational priming connections, and the volitional signals that are needed to convert subthreshold primes into suprathreshold top-down signals. Section 7 provides a detailed, but non-mathematical, exposition of all the ARTSCAN Search neural mechanisms. This section also lists the equation numbers for the corresponding model equations that are defined in the Appendix, and provides pointers to the relevant model circuit diagrams. This three-way coordination of expository information is aimed at making the model more accessible. Section 8 describes computer simulations of Where’s Waldo capabilities of the final ARTSCAN Search model. Section 9 provides a discussion and comparison with alternative models. Finally, the Appendix summarizes the model’s mathematical equations and parameters.

## 2. Some key issues

Many neuroanatomical, electrophysiological, and lesion studies have supported the hypothesis that two parallel, but interacting, visual cortical systems exist (Ungerleider and Mishkin, 1982; Mishkin et al., 1983; Goodale and Milner, 1992). Starting from primary visual cortex, the dorsal Where stream passes through the parietal cortex and controls processes of spatial localization and action. The ventral What stream passes through the inferotemporal cortex and carries out processes of object learning, recognition, and prediction. The inferotemporal cortex and its cortical projections learn to recognize *what* visual objects are in the world, whereas the parietal cortex and its cortical projections learn to determine *where* objects are and *how* to locate them, track them through time, and direct action toward them.

### 2.1. The view-to-object binding problem

Accumulating evidence supports the hypothesis that the brain learns about individual views of an object, coded by “view-tuned units.” As this happens through time, neurons that respond to different views of the same object learn to activate the same neuronal population, creating a “view-invariant unit.” In other words, the brain learns to link multiple view-specific categories of an object to a view-invariant categorical representation of the object (Baloch and Waxman, 1991; Bülthoff and Edelman, 1992; Seibert and Waxman, 1992; Tanaka, 1993; Logothetis et al., 1994; Bradski and Grossberg, 1995; Bülthoff et al., 1995; Carpenter and Ross, 1995; Riesenhuber and Poggio, 2000; Hung et al., 2005).

Many view-based models have focused on changes in retinal patterns that occur when a three-dimensional (3D) object rotates about its object-centered axis with respect to a fixed observer. However, complex objects are often actively explored with saccadic eye movements. When we consider how eye movements help us to learn about an object, a fundamental *view-to-object binding problem* must be confronted.

How does the brain know when the views that are foveated on successive saccades belong to the same object, and thereby avoid the problem of erroneously learning to classify parts of different objects together? How does the brain do this without an external teacher under the unsupervised learning conditions that are the norm during many object learning experiences *in vivo*?

### 2.2. Coordinating spatial and object attention during view-invariant category learning

The ARTSCAN model proposes how the view-to-object binding problem may be solved through the coordinated use of spatial and object attention. Several authors have reported that the distribution of spatial attention can configure itself to fit an object’s form. Form-fitting spatial attention is sometimes called an *attentional shroud* (Tyler and Kontsevich, 1995). ARTSCAN explains how an object’s preattentively formed surface representation can induce a form-fitting attentional shroud that is predicted by the model to accomplish two things:

First, a shroud enables eye movements to lock spatial attention onto an object of interest while they explore salient features on the object’s surface, thereby enabling different view-specific categories of the same object to be learned and then linked via associative learning to an emerging view-invariant object category. Consistent psychophysical data of Theeuwes et al. (2010) show that, indeed, the eyes prefer to move within an object rather than to an equally distant different object, other things being equal. Other data show that successive eye movements are not random, but rather tend to be attracted to salient features, such as bounding contours, corners, intersections, and boundary high curvature points (Yarbus, 1961; Jonides et al., 1982; Gottlieb et al., 1998; Krieger et al., 2000; Fecteau and Munoz, 2006). Consistent with these data, the ARTSCAN model predicts, as explained in section 3, how the surface contour signals that initiate figure-ground separation (Grossberg, 1994, 2007) may be used to compute target positions at salient features of an object that provide the most information for the view-specific category learning that then gets linked to a view-invariant object category.

Second, a shroud keeps the emerging view-invariant object category active while different views of the object are learned and associated with it. This is proposed to happen through a temporally coordinated cooperation between the brain’s What and Where cortical processing streams: The Where stream maintains an attentional shroud through a *surface-shroud resonance* that is supported by positive feedback signals between cortical areas V4 and PPC, among other brain regions. When an object’s surface is part of a surface-shroud resonance, spatial attention is focused on it. When the eyes fixate a particular view of the attended object, a view-specific category is learned by the What stream, say in ITp. This category focuses object attention via a learned top-down expectation on the critical features in the visual cortex that will be used to recognize that view and its variations in the future. When the first such view-specific category is learned, it also activates a cell population at a higher cortical level, say ITa, that will become the view-invariant object category.

Suppose that the eyes or the object move sufficiently to expose a new view whose critical features are significantly different from the critical features that are used to recognize the first view. Then the first view category is reset, or inhibited. This happens due to the mismatch of its learned top-down expectation, or prototype of attended critical features, with the newly incoming view information to the visual cortex (Grossberg, 1980a, 2012; Carpenter and Grossberg, 1987, 1991). This top-down prototype focuses object attention on the incoming visual information. Object attention hereby helps to control which view-specific categories are learned by determining when the currently active view-specific category should be reset, and a new view-specific category should be activated. However, the view-invariant object category should *not* be reset every time a view-specific category is reset, or else it can never become view-invariant by being associated with multiple view-specific categories. This is what the attentional shroud accomplishes: It inhibits a tonically-active reset signal that would otherwise shut off the view-invariant category when each view-based category is reset (Figure 1). As the eyes foveate a sequence of object views through time, they trigger learning of a sequence of view-specific categories, and each of them is associatively linked through learning with the still-active view-invariant category.

When the eyes move off an object, its attentional shroud collapses in the parietal cortex of the Where stream, thereby transiently disinhibiting a parietal reset mechanism that shuts off the view-invariant category in the What stream (Figure 1). When the eyes look at a different object, its shroud can form in the Where stream and a new view-specific category can be learned that can, in turn, activate the cells that will become a new view-invariant category in the What stream.

### 2.3. Supportive psychophysical and neurobiological data

The ARTSCAN model prediction that a spatial attention shift (shroud collapse) causes a transient reset burst in parietal cortex that, in turn, causes a shift in categorization rules (new object category activation) has been supported by experiments using rapid event-related functional magnetic resonance imaging in humans (Chiu and Yantis, 2009). These coordinated effects also provide a neurophysiological explanation of how attention can be disengaged, moved, and engaged by different object surfaces (Posner, 1980).

When a surface-shroud resonance forms, positive feedback from a shroud to its surface is also predicted to increase the contrast gain of the attended surface, as has been reported in both psychophysical experiments (Carrasco et al., 2000) and neurophysiological recordings from cortical areas V4 (Reynolds et al., 1999, 2000; Reynolds and Desimone, 2003). In addition, the surface-shroud resonance strengthens feedback signals between the attended surface and its generative boundaries, thereby facilitating figure-ground separation of distinct objects in a scene (Hubel and Wiesel, 1959; Grossberg, 1994, 1997; Grossberg and Swaminathan, 2004; Grossberg and Yazdanbakhsh, 2005). These experiments, and others summarized below, provide important psychophysical and neurobiological markers for testing predictions of the model.

## 3. ARTSCAN model main concepts

This section outlines the main concepts from the FACADE, ARTSCAN, pARTSCAN, and CogEM models that are unified and extended in the ARTSCAN Search model.

### 3.1. Image processing and spatial attention

Scenic inputs are processed in a simplified model retina/LGN by a shunting on-center off-surround network that contrast-normalizes the image. In the full FACADE model, and its extension and refinement by the 3D LAMINART model, object surface representations are formed in stages within the V1 blobs, V2 thin stripes, and V4. The current model does not consider 3D figure-ground separation of partially occluded objects, so can restrict its attention to a 2D filling-in process within the model analog of V2 thin stripes (Figure 1A, V2/V4) that is confined by object boundaries that form in the model analog of V2 pale stripes (Figure 1A, V2). The surfaces topographically activate spatial attention to induce a surface-fitting attentional shroud in the model PPC (Figure 1A, PPC) through a gain field (Figure 1A, LIP) that transforms the retinotopic coordinates of the surface into the head-centric coordinates of the shroud. This transformation maintains shroud stability during eye movements that explore different views of the object surface. In particular, the gain field is updated by predictive eye movement signals that are derived from *surface contour* signals (Figure 1, V2) from filled-in surfaces to their generative boundaries. Surface contour signals are generated by contrast-sensitive on-center off-surround networks that receive topographic inputs from their filled-in surface representations. Due to their contrast-sensitivity, they occur at the bounding contours of surface regions at which brightness or color values change suddenly across space.

### 3.2. Figure-ground separation and surface contour signals

Surface contour signals from a surface back to its generative boundaries strengthen the perceptual boundaries that will influence object percepts and recognition events, inhibit irrelevant boundaries, and trigger figure-ground separation (Grossberg, 1994, 1997; Kelly and Grossberg, 2000; Grossberg and Yazdanbakhsh, 2005). When the surface contrast is enhanced by top-down spatial attention (Figure 1A, PPC-LIP-V2/V4) as part of a surface-shroud resonance, its surface contour signals, because they are contrast-sensitive, become stronger, and thus its generative boundaries become stronger as well, thereby facilitating figure-ground separation. This feedback interaction from surfaces to boundaries via surface contour signals is predicted to occur from V2 thin stripes to V2 pale stripes, respectively.

### 3.3. Linking figure-ground separation to eye movement control

Corollary discharges are derived from these surface contour signal (Figure 1A, V3A, Nakamura and Colby, 2000; Caplovitz and Tse, 2007). They are predicted to generate saccadic commands that are restricted to the attended surface (Theeuwes et al., 2010) until the shroud collapses and spatial attention shifts to enshroud another object.

It is not possible to generate eye movements that are restricted to a single object until that object is separated from other objects in a scene by figure-ground separation. Various neurophysiological data support the idea that key steps in figure-ground separation occur in cortical area V2 (e.g., Qiu and von der Heydt, 2005). Thus, these eye movement commands are generated no earlier than cortical area V2. Surface contour signals are predicted to be computed in V2 (Grossberg, 1994). They are plausible candidates from which to derive eye movement target commands at a later processing stage because they are stronger at contour discontinuities and other distinctive contour features that are typical end points of saccadic movements. ARTSCAN proposes how surface contour signals are contrast-enhanced at a subsequent processing stage to choose the position of their highest activity as the target position of the next saccadic eye movement. The ARTSCAN model suggests that this choice takes place in cortical area V3A, which is known to be a region where vision and motor properties are both represented, indeed that “neurons within V3A… process continuously moving contour curvature as a trackable feature… not to solve the ‘ventral problem’ of determining object shape but in order to solve the ‘dorsal problem’ of what is going where” (Caplovitz and Tse, 2007, p. 1179).

### 3.4. Predictive remapping, gain fields, and shroud stability

These eye movement target positions are chosen before the eyes actually move. In addition to being relayed to regions that command the next eye movements, such as the FEF and SC (see Figure 1), they also maintain the stability of the active shroud in head-centered coordinates within the PPC, so that the shroud does not collapse every time the eyes move. They do this by controlling eye-sensitive gain fields that update the active shroud’s head-centered representation even before the eyes move to the newly commanded position. These gain fields thus carry out *predictive remapping* of receptive fields during eye movements. These ARTSCAN mechanisms new light on electrophysiological data showing perisaccadic (around the time of the saccade) remapping of receptive fields in parietal areas, including the lateral intraparietal cortex (LIP; Andersen et al., 1990; Duhamel et al., 1992) and the FEF (Goldberg and Bruce, 1990), as well as more modest remapping in V4 (Tolias et al., 2001). In particular, attended targets do not cause new transient activity in these regions after saccades (see Mathôt and Theeuwes, 2010 for a review). ARTSCAN predicts that the anatomical targets of these gain fields include an active shroud (viz., a form-sensitive distribution of spatial attention) in PPC that inhibits the reset of view-invariant object categories in ITa via a reset mechanism that transiently bursts when a shift of spatial attention occurs to a new object. This prediction suggests that manipulations of reset, such as those proposed by Chiu and Yantis (2009), be combined with manipulations of predictive remapping of receptive fields, such as those proposed by Andersen et al. (1990) and Duhamel et al. (1992).

## 4. pARTSCAN: positionally-invariant object learning and supportive neurophysiological data

**Figure 2:**
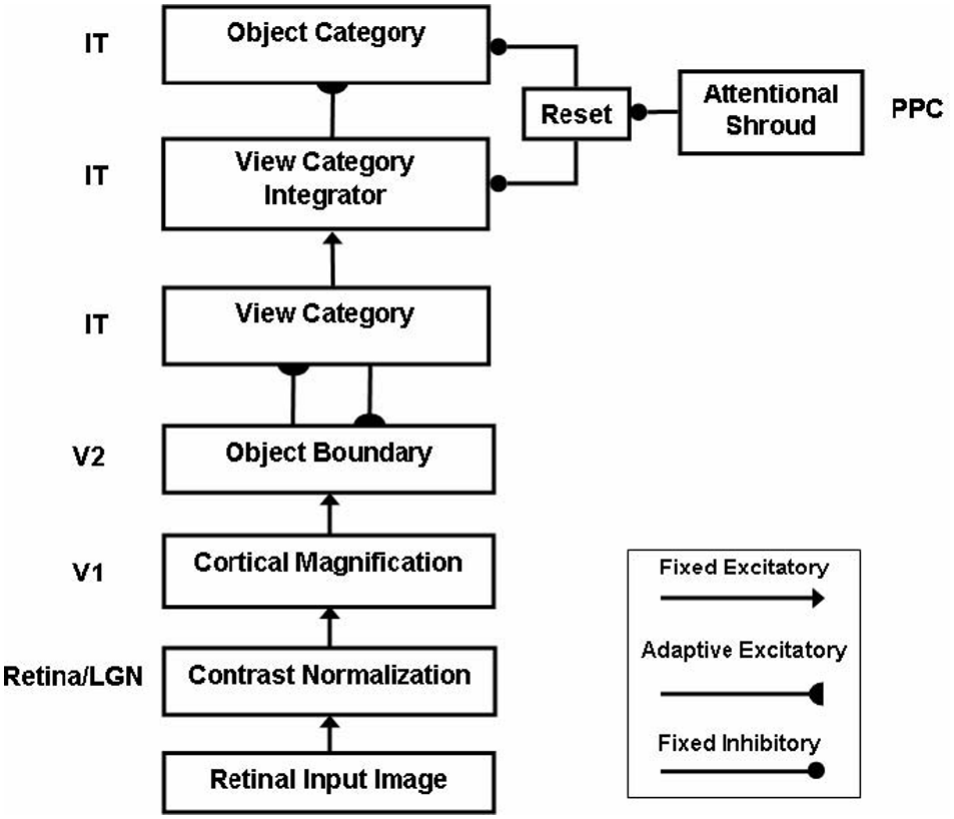
**Microcircuit of the pARTSCAN model (Cao et al., 2011; Figure 2)**. See text for details.

ARTSCAN does not explain how an object that is viewed at more peripheral retinal positions can be associated through learning with the same object category. However, peripheral vision makes important contributions to the execution of search tasks (Erkelens and Hooge, 1996). Electrophysiological data show that cells in the inferotemporal (IT) cortex respond to the same object at different retinal positions (Gross et al., 1972; Desimone and Gross, 1979; Ito et al., 1995; Booth and Rolls, 1998), and the selectivity to objects of an IT neuron can be altered by experiences with objects at such positions (Li and DiCarlo, 2008). The pARTSCAN extension of ARTSCAN (Cao et al., 2011), shown in Figure 2, explains how positionally-invariant object learning can be achieved.

pARTSCAN builds on ARTSCAN by proposing how the following additional processes in the What cortical processing stream enable both view-invariant and positionally-invariant object categories to be learned: IT cells with persistent activity, defined by view category integrator cells; and a combination of normalized object category competition and a view-to-object learning law which together ensure that unambiguous views have a larger effect on object recognition than ambiguous views. Persistently firing neurons in the inferotemporal cortex have been observed in neurophysiological experiments (Fuster and Jervey, 1981; Miyashita and Chang, 1988; Tomita et al., 1999; Brunel, 2003), but not given a functional interpretation in terms of positionally-invariant object category learning. pARTSCAN also simulates neurophysiological data of Li and DiCarlo (2008) from monkeys showing how unsupervised natural experience in a target swapping experiment can gradually alter object representations in IT. The swapping procedure is predicted to prevent the reset of the attentional shroud, which would otherwise keep the representations of multiple objects from being combined by learning.

The view category integrator stage in pARTSCAN model occurs between the view category and object category stages (Figure 2). A view category integrator cell, unlike a view-category cell, is not reset when the eyes explore new views of the same object. It gets reset when the invariant object category stage gets reset due to a shift of spatial attention to a different object.

**Figure 3:**
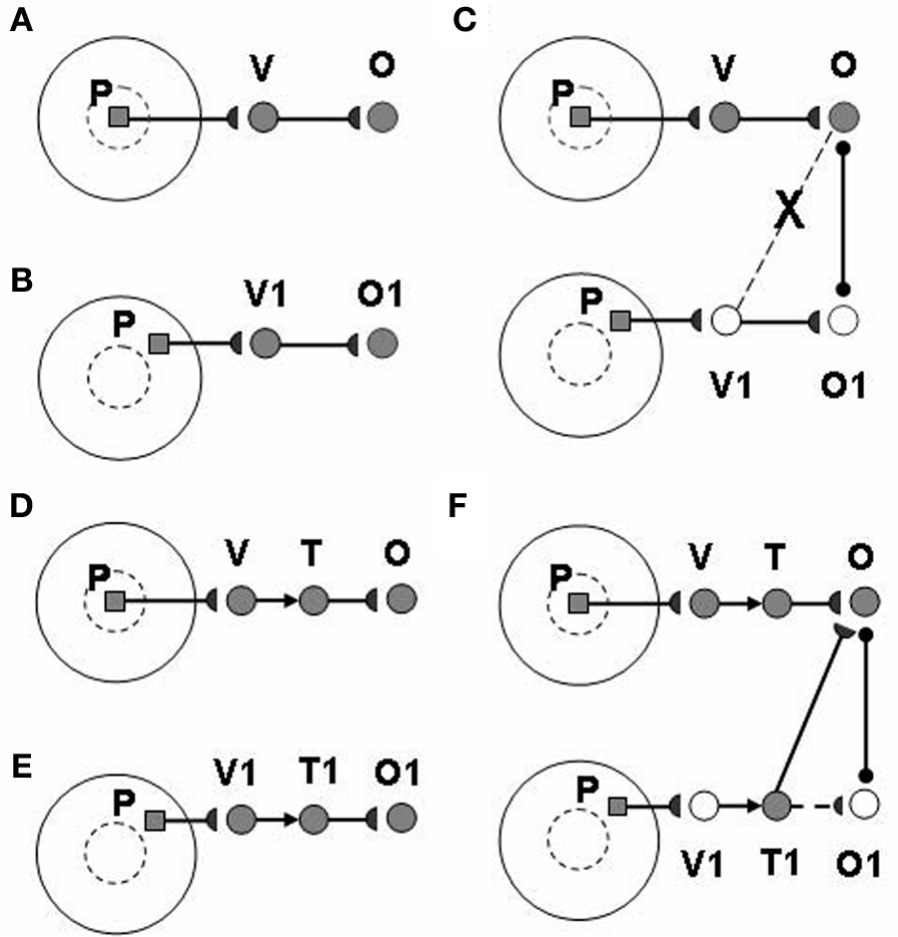
**How the view category integrator helps to learn a positionally-invariant object category**. See text for details. [Reprinted from Cao et al. (2011), Figure 4, with permission].

The view category integrator plays a key role in enabling learning of positionally-invariant object categories. Without the view category integrator, the following problem can occur: Suppose that a view of object P is generated by eye fixation in the fovea and sequentially triggers activations of view-specific category V and view-invariant object category O (Figure 3A). If the same object P appears in the periphery of the retina, as in Figure 3B, the model learns a new view-specific category V1 and in turns activates object category O1. Once a saccadic eye movement brings the object P into the foveal region (Figure 3C), it activates the previously learned view-specific category V and the object category O. Without the view category integrator, view category V1 is shut off with the saccade and it cannot learn to be associated with the object category O. As a result, object P learns to activate two object categories O and O1 corresponding to foveal and peripheral positions, respectively, and the same object at different positions can create different object categories. The view category integrator keeps the object from creating multiple object categorical proliferations. In Figures 3D,E, the view category integrators T and T1 preserve the activities of view categories V and V1 and learn connections to object categories O and O1. In Figure 3F, after the object P is foveated again, T1 is still active due to persistent activity, even though V1 is shut off by a saccade. Therefore, view category integrator T1 can be associated with object category O.

In summary, the pARTSCAN model predicts persistent activity in inferotemporal cortex (IT) that enables the model to explain how both view- and positionally-invariant object categories may be learned in cortical area ITa. The same process enables size-invariant categories to be learned. The target swapping experimental data of Li and DiCarlo (2008), which show that IT neuron selectivity to different objects gets reversed at the swap position with increasing exposure, can also be explained using these mechanisms. Finally, pARTSCAN can identify Waldo targets at non-foveated positions, but does not in itself show how these targets can lead to a shift of attention and foveation.

## 5. Joining invariant category learning with reinforcement learning and motivated attention

**Figure 4:**
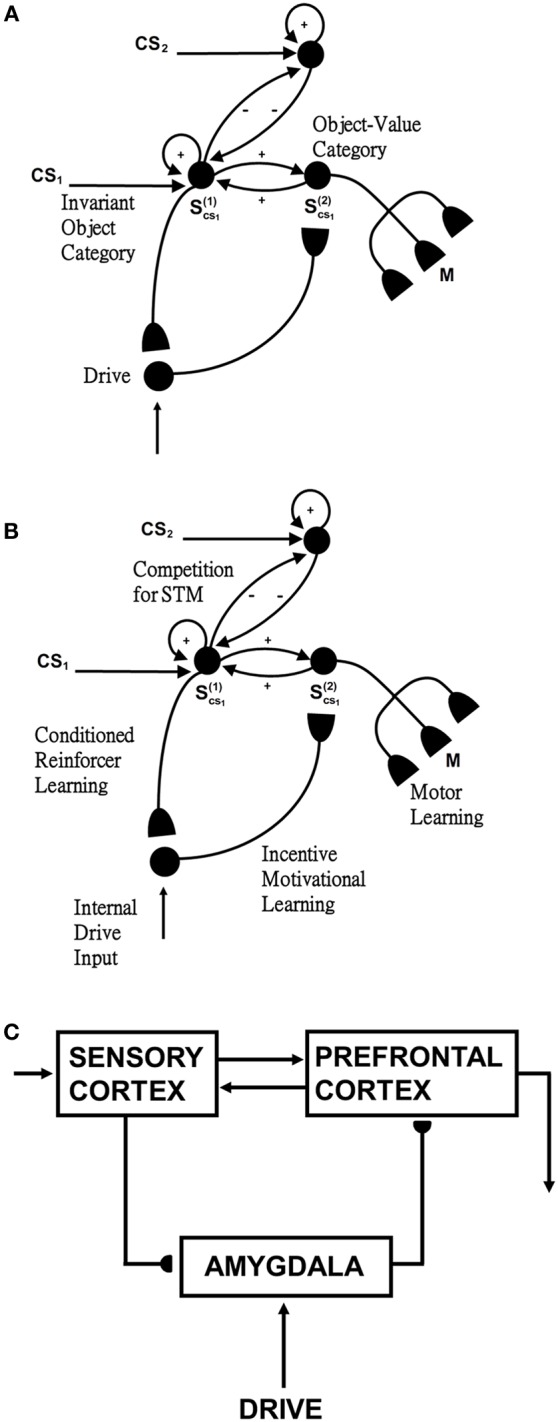
**Reinforcement learning circuit of the CogEM model (Grossberg, 1971, 1975; Grossberg and Seidman, 2006). (A)** Processing stages of invariant object category, object-value category, and drive representation (value category) representations. CS, conditioned stimuli; S, sensory representations; and M, motor representations. **(B)** Conditioned reinforcer learning enables sensory events to activate emotional reactions at drive representations. Incentive motivational learning enables emotions to generate a motivational set that biases the system to process information consistent with that emotion. Motor learning allows sensory and cognitive representations to generate actions. **(C)** Anatomical interpretations of the processing stages. [Adapted from Grossberg and Seidman (2006), Figures 4, 5, with permission].

The activation of an invariant recognition category by pARTSCAN mechanisms does not reflect the current emotional value of the object. Augmenting pARTSCAN with a CogEM circuit for reinforcement learning and motivated attention enables activation of an invariant category that is currently valued to be amplified by motivational feedback from the reinforcement learning circuit (Figure 4). Then the additional mechanisms of the ARTSCAN Search What-to-Where stream interactions can locate this motivationally salient object.

Such a CogEM circuit includes interactions between the inferotemporal cortex, orbitofrontal cortex, and amygdala (Figure 4C; Barbas, 1995). Activation of the feedback circuit through inferotemporal-amygdala-orbitofrontal interactions can create a resonance that focuses and maintains motivated attention upon a motivationally salient object category, while also supporting what Damasio has called “core consciousness” of goals and feelings (Grossberg, 1975, 2000; Damasio, 1999).

Such interactions were predicted by the CogEM, model, starting in Grossberg (1971), which simulates how sensory, or object, category representations (e.g., inferotemporal cortex, IT), drive, or value, representations (e.g., amygdala, AMYG), and object-value category representations (e.g., orbitofrontal cortex, ORB) interact via conditioned reinforcement, incentive motivational, and motor learning pathways (Figure 4). Various data support the prediction that drive-sensitive value category cells are found in the amygdala (Aggleton, 1993; LeDoux, 1993). Multimodal amygdala cells that are hunger and satiety selective (Muramoto et al., 1993; Yan and Scott, 1996) and respond in proportion to the value of a food reward have been extensively studied in the primate and rodent (Nishijo et al., 1988; Toyomitsu et al., 2002).

In the CogEM model, in response to visual cues, object-selective sensory representations in the inferotemporal cortex (Figures 4A,C) learn to activate drive representations in the amygdala via learned conditioned reinforcer pathways (Figures 4B,C). Activated drive representations can, in turn, activate the orbitofrontal cortex via learned incentive motivational pathways (Figure 4B). Motivationally salient sensory representations can hereby provide inputs directly to object-value representations (Figure 4A), and indirectly via the two-step learned conditioned reinforcer and incentive motivational pathway through the drive representations (Figures 4A,B). The incentive input determines how vigorously the object-value representation is activated (Rolls, 1999, 2000; Schoenbaum et al., 2003). The most active object-value representations can then select, and focus attention upon, motivationally consistent sensory representations. This selection process is driven by positive feedback from the object-value representations to their sensory representations, combined with competition among the sensory representations (Figure 4A). The motivationally most salient sensory representations can, in turn, attentionally block irrelevant sensory cues.

In summary, the CogEM model simulates how an invariant object category that is learned by pARTSCAN can learn to trigger an inferotemporal-amygdala-orbitofrontal resonance, thereby enabling motivationally enhanced activation of the invariant object category via top-down attentive feedback from the orbitofrontal cortex. Within the additional circuitry of the ARTSCAN Search model, a name category can prime the corresponding orbitofrontal object-value cells to initiate the process whereby a motivationally-enhanced top-down attentional priming signal triggers search for the valued object in the scene.

## 6. ARTSCAN search: bottom-up and top-down search from the what-to-where streams

**Figure 5:**
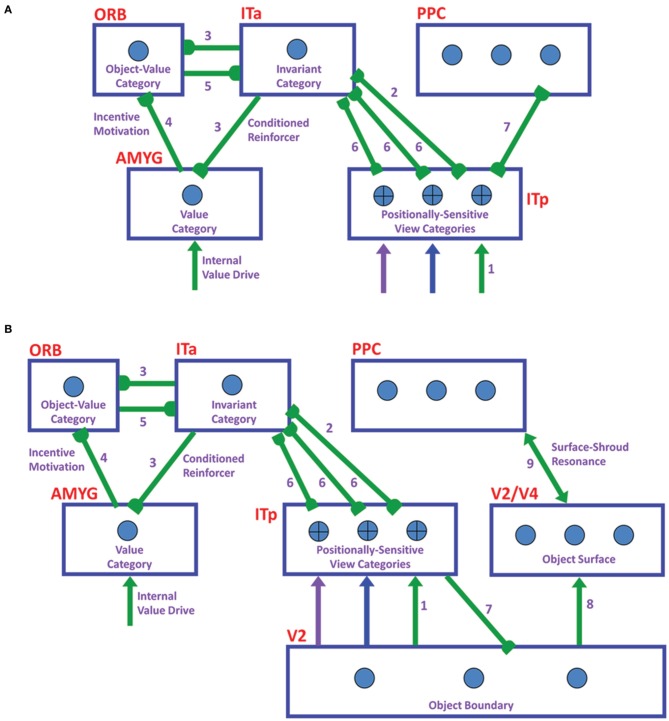
**Bottom-up stimulus-driven What stream recognition to Where stream search and action through (A) a direct What-to-Where pathway and (B) an indirect What-to-Where pathway**. Interactions between multiple brain regions, such as ITa, ITp, amygdale, and orbitofrontal cortex (ORB) in the What stream guide Waldo discovery in the posterior cortex (PPC) in the Where stream. The numbers indicate the order of pathway activations. See text for details. [Figure A is adapted with permission from Grossberg (2009), Figure 6].

Six different routes can, in principle, drive a Where’s Waldo search (Figure 5): bottom-up direct and indirect routes; top-down cognitive direct and indirect routes; and top-down motivational direct and indirect routes. For completeness, the model was simulated for all six routes, and it was shown that the direct routes can operate more quickly than the indirect routes.

### 6.1. Bottom-up direct route

First, bottom-up scenic inputs activate ITp cells that learn view- and positionally-specific categories. These cells also topographically project to PPC, where the target locations of an object are represented (Figure 5A). This is one of the What-to-Where stream interactions in the model.

Second, ITp cells activate view- and positionally-invariant object categories in ITa. These invariant object categories are learned using the Where-to-What stream interactions of the pARTSCAN model whereby an attentional shroud in PPC modulates the activity of an emerging invariant object category in ITa as sequences of view-specific categories of the object are activated, learned, and reset in ITp using reciprocal Adaptive Resonance Theory, or ART, connections between ITp and ITa (Figure 5A). Even if all the objects in the scene are equally salient, they can activate their invariant object categories because of the nature of the *normalized quenching competition* that occurs among all the categorical processing stages (see section 7.3.7). However, they cannot yet activate an eye movement to foveate one of them.

Third, ITa cells activate AMYG and send inputs to ORB.

Fourth, convergent ITa and AMYG inputs together can activate the corresponding ORB object-value category cells (Grossberg, 1975, 1982; Barbas, 1995, 2000; Schoenbaum et al., 2003) using learned incentive motivational signals from the AMYG. In other words, incentive motivation can amplify activation of a valued object-value category.

Fifth, an activated ORB object-value category can draw motivated attention to a valued object by sending top-down attentional signals back to its ITa source cells. Typically, such top-down attentional signals are modulatory. However, when combined with volitional signals from the BG, they can generate suprathreshold activation of the target ITa cells, thereby enabling the feedback loop between ITa, AMYG, and ORB to close. As a result, a valued ITa invariant object category may be motivationally amplified by an inferotemporal-amygdala-orbitofrontal resonance, which enables it to better compete for object attention with other ITa representations.

Sixth, the amplified ITa cells can then send larger top-down priming signals to all of its ITp representations. The ITp representation whose position corresponds to the valued object is selectively amplified due to the amplification of its bottom-up input from the object by the top-down attentional prime.

Seventh, these selectively amplified ITp cells can send amplified signals to the object position that is represented in the PPC. PPC activation draws spatial attention to that position, which can elicit an eye movement to foveate the desired object.

### 6.2. Bottom-up indirect route

The sequence from step one to step six in the bottom-up indirect route is the same as for the bottom-up direct route except the ITp cells do not project directly to the PPC (Figure 5B).

Seventh, the selectively amplified ITp cell corresponding to the target position provides top-down excitatory feedback to selectively prime the boundary representation of the Where’s Waldo target object. This boundary representation is hereby enhanced in strength relative to other object boundaries in the scene.

Eighth, the enhanced boundary representation gates the object’s surface filling-in process and thereby increases the contrast of the selected target surface.

Ninth, the enhanced surface representation projects to the PPC to facilitate its competition for spatial attention. As a surface-shroud resonance forms, the target surface can competitively win to form an active shroud which draws spatial attention and an eye movement to the target position.

### 6.3. Top-down cognitive direct route

**Figure 6:**
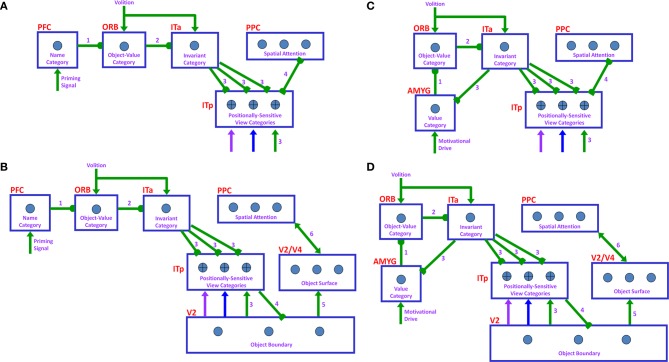
**Top-down name-driven What stream recognition to Where stream search and action through (A) a direct What-to-Where pathway and (B) an indirect What-to-Where pathway**. Top-down value-driven What stream recognition to Where stream search and action through **(C)** a direct What-to-Where pathway, and **(D)** an indirect What-to-where pathway. See text for details.

Many experiments have shown that top-down mechanisms play an important role in visual processing (e.g., Tomita et al., 1999; Barceló et al., 2000; Miyashita and Hayashi, 2000; Ranganath et al., 2004). The ARTSCENE Search model clarifies how such mechanisms may play an important role during a Where’s Waldo search (Figure 6).

In particular, when the name of a desired object is presented to the model, the corresponding name category neuron in PFC can top-down prime the object-value category in ORB (Figure 6A). When BG volitional signals are also activated, this prime can supraliminally activate ORB cells which can, in turn, prime the corresponding view-invariant object category neuron in ITa. Here too a volitional signal can enable the prime to supraliminally activate the primed ITa cells, which can then activate all compatible positionally-selective view categories in ITp. This prime can amplify the ITp category that receives a match from the bottom-up Waldo input. Then the selected category can activate the corresponding position in PPC, which can direct an eye movement and other actions toward Waldo (Figure 6A).

### 6.4. Top-down cognitive indirect route

This route executes the same top-down pathway as the cognitive direct route from the desired name category neuron to selectively amplify the view-specific category neurons in ITp via the object-value category cells in ORB and view-invariant object category neurons in ITa. The amplified ITp cell activates the same pathways as the bottom-up indirect route from the seventh to ninth steps to create a surface-shroud resonance corresponding to the target object and leading to foveation of this object (Figure 6B).

### 6.5. Top-down motivational direct route

An object-value category in ORB can be primed by a value category in AMYG via incentive motivational signals (Figure 6C). Then the same process is activated as for the cognitive prime above.

### 6.6. Top-down motivational indirect route

This route performs similar interactions as the top-down cognitive indirect route except the initial stage begins with priming from the value category in AMYG (Figure 6D).

## 7. Model description

The ARTSCAN Search model incorporates and unifies the following innovations that go beyond the structure of the ARTSCAN model:

1. The gain field stage, which mediates the coordinate transformation between a retino-centric object surface representation and a head-centric spatial attention map, is processed by separate and parallel bottom-up and top-down channels, instead of combining them linearly in a single stage, as in ARTSCAN. See section 7.2.1.
2. As in pARTSCAN (Figure 2), a view category integrator stage occurs after the view category stage in the What stream to enable positionally-invariant as well as view-invariant categories to be learned. View category integrator neurons preserve view-specific category neural activities while the eyes scan the same object, and thereby enable view-specific categories of the same object at different positions to be associated with the same view-invariant object category. See section 7.3.2.
3. Reset is triggered when the total shroud activity reduces below a threshold value due to activity-dependent habituation in the surface-shroud feedback loop. The reset wave is extended to nonspecifically inhibit the spatial attentional map in PPC and the object surface representation in V4, not just ITa, as in ARTSCAN. Such a reset mechanism can more efficiently shut of the entire current surface-shroud resonance to allow a smooth attention shift to another object surface. In addition, as in pARTSCAN, the reset signal inhibits the currently active view category integrator neurons. See section 7.2.4.
  Because the reset mechanism in the Where stream can inhibit the spatial attentional map, it is rendered transient by being multiplied, or gated, by a habituative transmitter. Otherwise, it could tonically inhibit the spatial attentional map and prevent the next object from being spatially attended. In contrast, the reset mechanism in the What stream is not gated by a habituative transmitter. This ensures that the view-specific categories of a newly attended object cannot be spuriously associated with the invariant object category of a previously attended object.
4. Value category and object-value category processing stages from the CogEM model (Figure 4) are added to enable valued categories to be motivationally amplified and attended, thereby facilitating their selection by an inferotemporal-amygdala-orbitofrontal resonance. See sections 7.3.3–7.3.5.
5. As in CogEM, there are adaptive conditioned reinforcer learning pathways from invariant object categories in ITa to value categories in AMYG, and incentive motivational learning pathways from AMYG to object-value categories in ORB. In addition, and beyond CogEM, ITa can also send adaptive excitatory projections to ORB to enable one-to-many associations to be learned from a given object representation to multiple reinforcers.
6. Top-down pathways and BG volitional control signals (Figure 6) together enable a top-down search for Waldo to occur from the What stream to the Where stream. The volitionally-enhanced excitability enables modulatory priming stimuli to fire their target cells and send thereby send top-down signals to lower processing stages.

**Figure 7:**
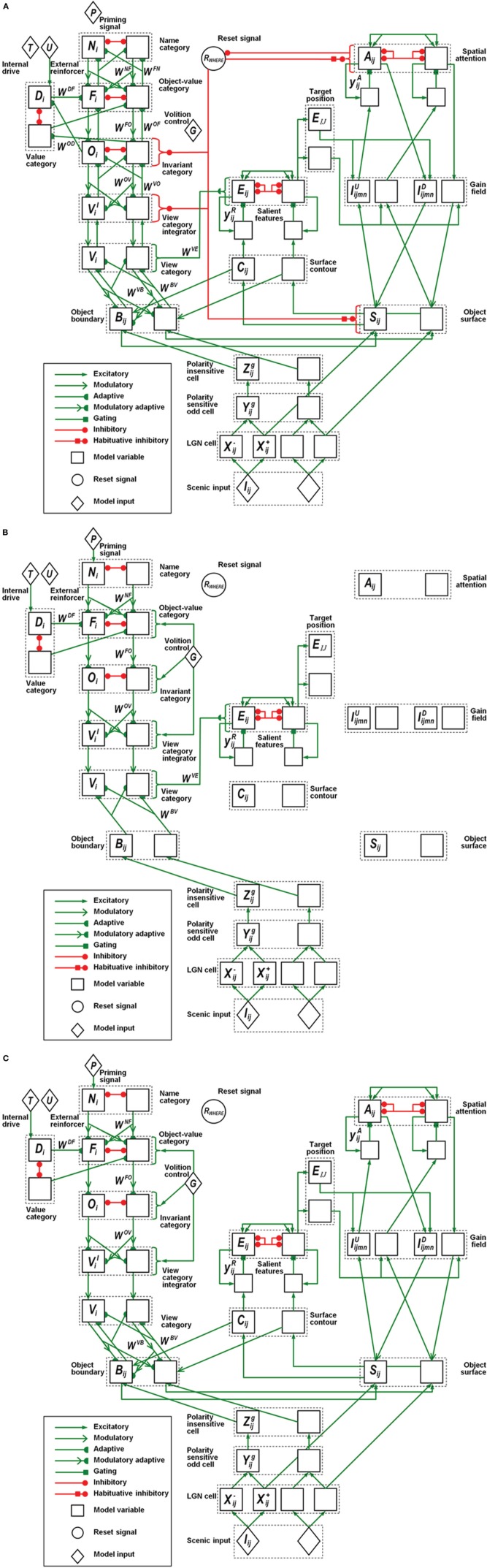
**Model variables and their computational relations. (A)** Category learning. **(B)** Direct pathway of top-down primed search. **(C)** Indirect pathway of top-down primed search. The dashed boxes correspond to the layers of the box diagram in Figure 1. Each layer has two neurons indicating the connections to the neighboring layers. Different types of connections correspond to excitatory, adaptive, or inhibitory effects between two layers. The letter inside each neuron refers to the variables or the constant values specified in the Appendix.

In all, the ARTSCAN Search model includes three component networks: (1) Boundary and Surface Processing, (2) WHAT Stream, and (3) WHERE Stream. Each component consists of several processing stages. Figure 1 shows a block diagram of the main model processing stages. Figure 7 illustrates model circuit interactions more completely.

### 7.1. Retina and primary visual cortex processes

#### 7.1.1. Retina and LGN polarity-sensitive cells

Input preprocessing is simplified to include only properties needed to carry out the category-level simulations that are the focus of the article. The model retina and LGN are accordingly lumped together. Together they normalize contrast of the input pattern using polarity-sensitive ON and OFF cells. ON (OFF) cells obey cell membrane, or shunting, equations that receive retinal outputs and generate contrast-normalized activities that discount the illuminant using multiple-scales of on-center off-surround (off-center on-surround) networks, respectively [Equations (A4–A8)]. These cells input to the simple cells in the model’s cortical area V1.

#### 7.1.2. V1 polarity-sensitive oriented simple cells

The polarity-sensitive simple cells [Figure 7; Equations (A9–A14)] in primary visual cortical area V1 (Hubel and Wiesel, 1959, 1962) have elongated excitatory and inhibitory zones that form an oriented receptive field and produce a multiple-scale boundary representation of the image by processing the multiple-scale unoriented output signals from the LGN. Each receptive field consists of polarity-sensitive ON- and OFF-subregions. The ON-subregions receive excitatory ON LGN signals and inhibitory OFF LGN signals, while the OFF-subregions have the converse relation to the LGN channels (Hubel and Wiesel, 1962; Grossberg and Todorović, 1988; Reid and Alonso, 1995; Hirsch et al., 1998; Raizada and Grossberg, 2001).

#### 7.1.3. V1 polarity-insensitive complex cells

Rectified output signals from opposite-polarity like-oriented simple cells at each position input to complex cells, which are therefore polarity-insensitive oriented detectors that are processed at multiple spatial scales [Figure 7; Equations (A15–A17)].

#### 7.1.4. V2 boundaries and surface-to-boundary attentional priming

Because the 2D image database we simulated does not have illusory or missing contours or occlusions, the model simplifies the computation of object boundaries by omitting depth-selective disparity tuning processing in cortical area V1 and boundary completion processing in V2.

Object boundaries [Figure 7; Equations (A18–A20)] are modeled as V2 pale stripe neurons that receive multiple-scale bottom-up inputs from V1 complex cells. These boundaries multiplicatively gate a surface filling-in process, again at multiple scales, within model V2 thin stripe neurons. These boundary-to-surface signals contain the filling-in of surface brightnesses and colors within their borders. The boundaries are also gain-amplified by surface-to-boundary surface contour feedback signals [Figure 7; Equations (A27–A31)]. Top-down attention from a surface-shroud resonance can increase the perceived contrast of an attended surface, which increases the strength of the corresponding surface contour signals, thereby strengthening attend object boundaries as well, while weakening the boundaries of non-attended surfaces. Object boundaries also project to the What stream, where their adaptive pathway embody the learning of view-specific categories in cortical area ITp.

#### 7.1.5. V2 surface filling-in

The filling-in of object surface activities in V2 thin stripe cells takes place within Filling-In Domains (FIDOs) [Figure 7; Equations (22–25)]. Filling-in is activated bottom-up by multiple-scale ON and OFF LGN inputs that activate different FIDOs (Cohen and Grossberg, 1984; Grossberg and Todorović, 1988; Grossberg, 1994).

A weighted sum across the multiple scales of the surface representations [Equation (A26)] generates topographic outputs to the spatial attention region in PPC, where these PPC inputs competitively bid to form a winning attentional shroud. The winning shroud delivers positive feedback to the corresponding surface representation, thereby inducing a surface-shroud resonance that locks spatial attention upon that surface while increasing its contrast.

Successfully filled-in surfaces generate contour-sensitive output signals via surface contours. Surface contours are computed by inputting the filled-in surface activities to a contrast-sensitive on-center off-surround shunting network [Equations (A27–A31)]. The surface contour outputs project back to their generative object boundaries across all scales. As noted in section 7.1.4, when a surface is attended as part of a surface-shroud resonance, its enhanced contrast increases its surface contour outputs which, via surface-to-boundary feedback, strengthens the corresponding boundaries and inhibits the boundaries of unattended surfaces.

The surface-shroud resonance can be inhibited at the FIDOs by a reset signal from the Where processing stream.

### 7.2. Where stream

A surface-shroud resonance in the Where stream ensures that successive eye fixations are restricted to salient features within the attended surface. These fixations enable the learning of multiple view-specific categories of the object, which can all be associated with the emerging view- and positionally-invariant object category until shroud collapse, and a shift of spatial attention away from the object, cause the invariant object category to be inhibited due to transient disinhibition of the category reset mechanism.

#### 7.2.1. Gain field

Keeping the view-invariant object category active during these sequential saccades within the object requires that the reset mechanism continuously receives a sufficient amount of inhibition from the currently active shroud. In pARTSCAN, the surface representation is computed in retinotopic coordinates that change during a saccade. If all the coordinates of the shroud changed as well, reset could occur whether or not a saccade landed within the same object. Maintaining inhibition of reset is facilitated by computing shrouds in head-centric coordinates. The coordinate transformation from retinotopic to head-centered coordinates uses gain fields (Figure 7), which are known to act on the parietal cortex, notably the lateral intraparietal area (LIP), among other brain regions (Andersen et al., 1985; Colby et al., 1993).

A number of neural models have been proposed for how the outflow commands that control eye movements also activate a parallel corollary-discharge pathway which computes gain fields that transform retinotopic coordinates into head-centered coordinates (Grossberg and Kuperstein, 1986, 1989; Zipser and Andersen, 1988; Gancarz and Grossberg, 1999; Pouget and Snyder, 2000; Xing and Andersen, 2000; Mitchell and Zipser, 2003; Pouget et al., 2003; Cassanello and Ferrera, 2007). Equations (A32–A36) mathematically describe the gain field transformation that is used in this article.

#### 7.2.2. Spatial attention: attentional shroud

The head-centric spatial attention neurons [Figures 1, 7; Equations (A37–A41)] receive bottom-up input from gain field neurons. The spatial attention neurons select a winning shroud through recurrent on-center off-surround interactions whose short-range excitations and surface-shroud positive feedback keep the winning shroud active, while longer-range off-surround feedback inhibits other spatial attentional neurons. The top-down feedback from the selected shroud neurons reaches object surface neurons through gain field neurons. This surface-shroud gain-field-modulated resonant feedback loop links retinotopic surface representations with head-centric spatial attentional shrouds. It is the neural event that corresponds to focusing spatial attention on the object surface.

Decay of an active shroud’s activity below a threshold value triggers a reset signal which, in turn, sends a nonspecific inhibitory signal back to the spatial attention network to ensure that the shroud is totally inhibited. However, the reset mechanism, in the absence of other factors, is tonically active [Equation (A55)]. In order to prevent reset-mediated inhibition from persisting indefinitely due to its tonic inhibition of the spatial attention network, all Where stream reset signals are multiplied, or gated, by an activity-dependent habituative transmitter that causes the gated reset signal to be transiently active [Equations (42, 47)]. Such a transmitter multiplies the reset signal, so when it collapses due to sufficient recent activity of the reset signal, the net reset signal collapses too. After the transient reset signal collapses, spatial attention can shift to another object and the cycle of attention shifting and invariant category learning can continue.

#### 7.2.3. Eye movements to salient surface features and inhbition-of-return

The salient feature neurons [Figure 7; Equations (A43–A46)] receive their largest inputs from the surface contour neurons whose activities are amplified by the active shroud. The surface contour neurons hereby play two roles: (1) they strengthen the boundaries of an attended surface while also inhibiting unrelated boundaries via surface-to-boundary feedback, and (2) they activate a parallel pathway, hypothesized to involve cortical area V3A, that converts the salient features into target positions of saccadic eye movements aimed at the attended surface. This conversion is carried out by a contrast-enhancing recurrent on-center off-surround shunting network that chooses the most active position on the surface contour. This position marks the most salient feature at that time, as well as an “attention pointer” (cf. Cavanagh et al., 2010) to the target position of the next saccade. In this way, the eyes move to foveate the most salient features on the attended object, like corners and intersections.

The eye movement map is gated by habituative transmitters [Equation (A47)]. Once the eyes foveate a saccadic target position, these transmitters deplete in an activity-dependent way, thereby enabling another eye movement neuron to win the competition for the next target on the attended surface. This habituative mechanism instantiates the concept of “inhibition-of-return (IOR)” by preventing perseveration of eye movements to the same object position.

#### 7.2.4. Object category reset by transient parietal burst

The reset-activated pathways to both the object surfaces and the spatial attention network are also gated by activity-dependent habituative transmitters [Equation (A52)]. These habituative gates facilitate the collapse of an active surface-shroud resonance after a period of sustained spatial attention directed toward the corresponding object surface. While an attentional shroud is active, the currently active neurons within that shroud inhibit the category reset neurons. The category reset stage [Equations (A50, A51)] in the Where stream is modeled by a tonically active neuronal population that nonspecifically inhibits the region where invariant object categories are learned within cortical area ITa of the What stream. The attended invariant object category can remain active because the category reset stage is inhibited by the currently active shroud. When the currently active shroud collapses, the category reset neurons are disinhibited, thereby enabling reset signals to inhibit the currently active invariant object category, as well as the currently active shroud. As a result of this transient reset burst, a shift of spatial attention can enable a correlated shift in categorization rules (Yantis et al., 2002; Serences and Yantis, 2006; Chiu and Yantis, 2009).

In the ARTSCAN Search model, unlike the ARTSCAN model, the reset signals are delivered to the view category integrators, invariant object categories, object surfaces, and spatial attention neurons. Reset may be initiated after only part of a shroud collapses, using a ratio reset rule that is more sensitive to the global structure of the shroud than was used in the ARTSCAN model. Due to the inhibition by the reset signal of the surface-shroud resonance itself, the more the attentional shroud collapses, the more the reset activity is disinhibited. This disinhibitory feedback loop enables fast and complete collapse of the currently active surface-shroud resonance, and a shift of attention to another object surface.

### 7.3. What stream

The What cortical stream in the ARTSCAN Search model includes several different kinds and sites of learning (Figure 7). First, there is view- and positionally-specific category learning in cortical area ITp. Second, there is view- and positionally-invariant category learning in cortical area ITa. Third, there is object-value learning from ITa to the orbitofrontal cortex (ORB). Fourth, there is conditioned reinforcer learning from ITa to the amygdala (AMYG). Fifth, there is incentive motivational learning from AMYG to ORB.

There are two types of reset events during category learning: First, there are the Where-to-What stream resets of the view- and positionally-invariant categories in ITa, discussed above, that are triggered by a surface-shroud collapse between V4 and PPC. Second, What stream resets of the view- and positionally-selective categories in ITp are mediated by sufficiently big mismatches of bottom-up visual input patterns with the top-down expectations that are read out to visual cortex from the currently active view- and positionally-selective categories (Carpenter and Grossberg, 1991; Grossberg, 2012).

The What stream also includes other top-down expectations that are used to perform a Where’s Waldo search (Figures 1, 6). These expectations carry priming signals from name categories in PFC to object-value categories in ORB, then to view- and positionally-invariant object categories in ITa, and finally to view- and positionally-specific categories in ITp. All of these top-down signals are modulatory: Without additional inputs to enhance them, they cannot fire their target cells. BG volitional signals enable the object-value and invariant object categories to fire when such top-down priming signals are also active. The subset of primed view-specific categories that receive bottom-up sensory inputs can also fire, and thereby activate the corresponding positions in PPC via a What-to-Where stream interaction, which leads to competitive selection of the most active position, and then a saccadic eye movement to that position.

#### 7.3.1. View-specific categories

The view-specific category neurons, which are proposed to be computed in cortical area ITp, receive inputs from an object’s boundaries, which are proposed to be computed in the pale stripes of cortical area V2 [Figure 7; Equations (A55–A60)]. Each view-specific category learns to encode a range of boundary shapes, sizes, and orientations may be experienced when foveating different gaze positions of the same object view. View-specific categories are learned using an Adaptive Resonance Theory, or ART, classifier, notably *Fuzzy* ART (Carpenter et al., 1991, 1992), which is capable of rapidly learning and stably remembering recognition categories of variable generality in response to arbitrary sequences of analog or binary input patterns. Fuzzy ART includes learning within both a bottom-up adaptive filter that is tuned to cause category activation with increasing selectivity and vigor, and a top-down expectation that is matched against bottom-up input patterns to focus attention upon the set of critical features in the bottom-up input pattern that were previously learned by the top-down expectation. A big enough mismatch leads to reset of the currently active category via an orienting system. This reset triggers search for a new, or previously learned and better-matching, category with which to represent the current input. See Grossberg (2012) for a heuristic review of ART as a cognitive and neural theory.

As noted in section 6, view-specific categories can be activated during a Where’s Waldo search by either a bottom-up or a top-down route. The bottom-up route involves focusing motivated attention on the corresponding invariant object category via an ITa-AMYG-ORB resonance [Figure 5; Equations (A55–A71)]. The top-down routes involve top-down priming by a name category [Figures 6A,B; Equations (A72–A74)] via a PFC-ORB-ITa-ITp route or by a value category via an AMYG-ORB-ITa-ITp route. These top-down signals can selectively amplify the selected ITa representation which, in turn, sends larger top-down priming signals to its ITp representations. These ITp neurons correspond to different positions and views of the object. The view that is seen at a given position generates a bottom-up input that matches the corresponding top-down prime and can then better compete with other active ITp representations. The chosen ITp neuron can either activate a direct What-to-Where pathway from ITp to PPC to rapidly induce an eye movement [Figures 6A,C; Equation (A48)], or a longer path along an ITp-V2-V4-LIP-PPC route (Figures 6B,D) to direct the eye movement to desired target.

#### 7.3.2. View category integrators

Each view-specific category activates its own population of view category integrator neurons [Figures 1, 2, 3; Equation (A61)]. These integrators stay active as the eyes move to explore different views of the same attended object, even after their view-specific category is reset. View category integrator neurons are reset when the shroud corresponding to a given object collapses, attention shifts to another object, and the eyes begin to explore the new object.

As explained in section 4, these neurons were introduced in the pARTSCAN model to show how object category neurons could learn to be positionally-invariant as well as view-invariant (Cao et al., 2011).

#### 7.3.3. Invariant object categories

Object category neurons [Figures 1, 5, 6; Equations (A63–A65)] learn to become both view- and positionally-invariant due to the learning that occurs within the adaptive input signals that they receive from multiple view category integrator neurons; see section 4. This learning goes on as long as the view category integrator neurons are active. When attention shifts to another object, both the view category integrator neurons and the invariant object category neurons get reset, both to prevent them from being associated with another object, and to allow selective learning of many objects to occur.

Unlike the resets of Where stream spatial attention, these What stream resets are not gated by a habituative transmitter [Equations (A61, A63)]; rather, they are shut off by inhibition from the next shroud that forms. If What stream resets were transient, then the previously active invariant category could be reactivated during the time between the collapse of the previous shroud and the formation of the next shroud. As a reset, the previous invariant category could be erroneously associated with view-specific categories of the next object.

#### 7.3.4. Value categories

Invariant object category representations can be amplified by an ITa-AMYG-ORB resonance (Figures 4C, 5), which can focus motivated attention on objects that are valued at a particular time. Such a resonance can develop as a result of two types of reinforcement learning (Grossberg, 1971, 1972a,b, 1982), as summarized in section 5: First, pairing the object with a reinforcer can convert the object representation into a *conditioned reinforcer* by strengthening the connection from the active invariant object category in ITa to an active value category, or drive representation, in AMYG [Figures 1, 4, 5, 6; Equation (A78)]. Many neurobiological data support the hypothesis that AMYG is a value category (e.g., Aggleton, 1993; LeDoux, 1993; Muramoto et al., 1993; Yan and Scott, 1996). Conditioned reinforcer learning is *many-to-one* learning because multiple categories can be associated with the same drive representation, much as multiple types of foods can be associated with the motivation to eat.

#### 7.3.5. Object-value categories

The invariant object category in ITa can also send adaptive excitatory projections to object-value representations [Figures 1, 4, 5, 6; Equations (A70, A71)] in ORB (e.g., Barbas, 1995; Cavada et al., 2000; Rolls, 2000; Schoenbaum et al., 2003; Kringelbach, 2005). The adaptive nature of these connections is a new feature of the model, which enables associations to be learned from a given object representation to multiple reinforcers. A second many-to-one kind of learning in the model is incentive motivational learning. This type of learning can increase the incentive motivational signals from a value category in the AMYG to an object-value category in the ORB by strengthening the corresponding AMYG-to-ORB pathway. Motivationally salient invariant object category representations in ITa can hereby provide inputs directly to object-value representations in ORB, and indirectly via two-step learned conditioned reinforcer and incentive motivational pathways. Such favored object-value representations can generate positive feedback to the corresponding invariant object category representation via an ORB-to-ITa pathway [Equation (A77)]. This feedback amplifies the favored invariant object category in ITa and allows it to better compete for object attention, as occurs during attentional blocking experiments (Grossberg and Levine, 1987).

#### 7.3.6. Name categories

Name category neurons in PFC [Figures 1, 6; Equations (A72–A74)] learn to be associated with the corresponding object-value category neurons in ORB and can thus send excitatory priming feedback to the corresponding object-value category neurons to enhance their representations during a top-down Where’s Waldo search [Equation (A81)].

#### 7.3.7. Normalized quenching competitive dynamics during searches

The many-to-one nature of the learned connections between invariant object categories, value categories, and object-value categories could potentially cause problems during searches. Suppose, for example, that there were a winner-take-all competition at each of these processing stages. Choosing a winning view-specific category is needed, for example, to activate a single object’s boundary representation and thereby direct eye movements toward salient features of the boundary’s surface contours during indirect searches.

In apparent conflict with this useful property is how a winner-take-all choice can undermine motivational searches. During the initial bottom-up processing of a scene containing multiple objects of equal perceptual salience, there may be no clear winner of a winner-take-all competition. To break this tie, suppose that a winning view-specific category was arbitrarily chosen, say based on a random attentional spotlight. Suppose, moreover, that this view-specific category does not correspond to an invariant object category that was associated through reinforcement learning with the active value category during a motivational search. Then incentive motivational signals from the value category could prime all the object-value categories with which it was earlier associated. However, by itself, such a prime could not activate any of these categories because bottom-up input from an invariant object category corresponding to one of these object-value categories would also have to occur. However, if the winning view-specific category does not activate any of these object-value categories through its invariant object category, then the search could not continue.

This problem is overcome by incorporating mathematically proven properties of recurrent competitive dynamics among cells that obey the membrane equation, or shunting, dynamics of biological neurons (e.g., Grossberg, 1973, 1980b, 2013b). In particular, there exists a *quenching threshold* in such networks so that choices are not made in response to input activities that are too close to one another, but can be made in response to an input that is sufficiently bigger than its competitors. Moreover, such networks tend to *normalize* their total activities, whether or not a choice is made, using the automatic gain control property that follows from shunting dynamics. Normalization allows “weighing the evidence” among several equally salient alternatives. These properties is incorporated algorithmically in the competitive networks that determine the outputs of the view-specific categories [Equation (A60)], invariant object categories [Equation (A64)], value categories [Equation (A69)], object-value categories [Equation (A71)], and name categories [Equation (A74)]. This competition is henceforth called *normalized quenching competition*.

Given this refined competition property, in response to a bottom-up input from several equally salient inputs, the normalized network activity is divided equally among them. They can all activate their view-specific, invariant object, and object-value categories. Suppose that a value category now primes several object-value categories, but only one of them has a bottom-up input. Because it now receives a bottom-up input as well, this object-value category is selectively amplified and can win the competition among the object-value categories. The chosen object-value category can, in turn, enable the corresponding invariant object category to win its competition. The winning invariant object category can, in turn, prime all of its view-specific categories. Only one of these view-specific categories receives a bottom-up input, and this one can win its competition and drive either a direct or indirect eye movement to the position of the corresponding object.

The top-down cognitive and motivational searches also work because they enable a single object-value category to win its competition and thereby trigger the same top-down cascade of events that was just summarized.

## 8. Simulation results

**Figure 8:**
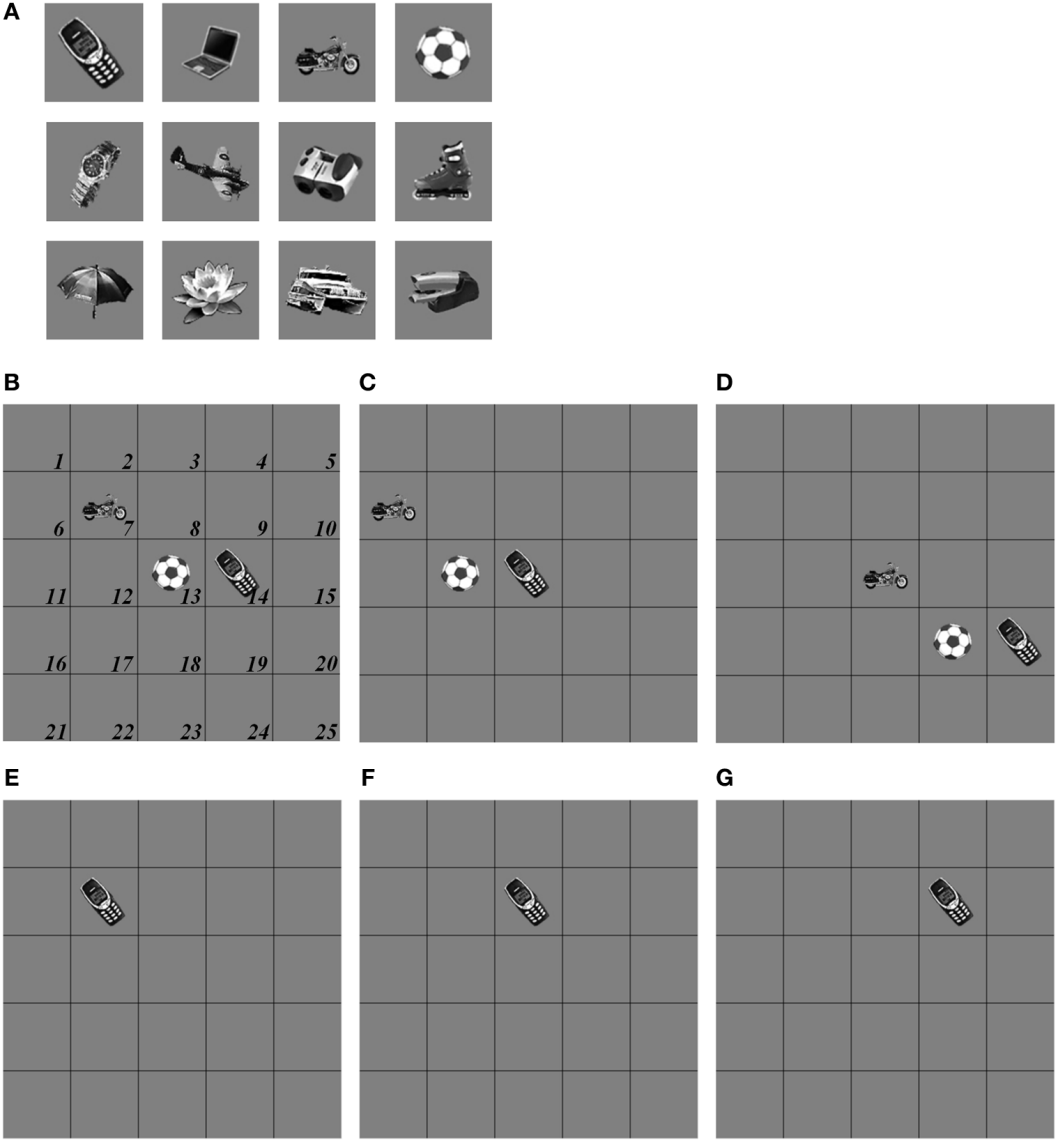
**Set of object stimuli for view- and positionally-invariant category learning. (A)** Each object reflects the relative size within 100 × 100 pixels from Caltech 101 dataset. **(B)** A simulated scene for simulations of view-invariant object category learning in section 8.1. A scenic input image is partitioned into 25 regions (solid lines) and objects are located in the central regions of the input scene (regions 7, 8, 9, 12, 13, 14, 17, 18, and 19). Region 5 is the foveal region and others are the peripheral regions. **(C)** The bottom-up input representations after cellphone becomes the attended object and is foveated. **(D)** The bottom-up input representation when motorcycle becomes foveated after the soccer ball and cellphone are learned. **(E,F)** A sequence of simulated scenes for simulations of positionally- and view-invariant object category learning in section 8.2. Each scenic input only contains one object located in one of the center regions.

The simulations of the ARTSCAN Search model demonstrate multiple sites of coordinated category, reinforcement, and cognitive learning, and use of the learned connections to carry out both bottom-up and top-down Where’s Waldo searches. ARTSCAN Search simulations process 24 objects taken from natural images of the Caltech 101 data base, with each object selected from different categories as Where’s Waldo exemplars. Each object is customized into 100 × 100 pixels (Figure 8A) against a homogeneous gray background with a luminance value of 0.5. The objects are in a gray scale with luminance values between 0 and 1. Input scenes are presented and simulated in Cartesian coordinates, for simplicity. A simulated scene is represented by 500 × 500 pixels and is divided into 25 regions of 100 × 100 pixels, with each region denoted as one position capable of representing one object.

The simulations are separated into three processes. The first process replicates view-invariant category learning of the ARTSCAN model. The purpose of the simulation is to show that the ARTSCAN Search model maintains the properties of the ARTSCAN model while adding the view category integrator stage and reinforcement and cognitive learning. This simulation allows us to observe the dynamics of how spatial attentional shrouds form and then collapse to trigger category reset, of how spatial attention shifts from one object to another, and of how the model learns view-invariant object categories as the eyes autonomously explore a scene. While each shroud is active, the eyes move to approximately 7–8 hotspots on the attended surface. The duration of each fixation is approximately 0.3 s until the eye movement map computes the next saccadic eye movement command.

Initially, three out of 24 objects are randomly chosen and scattered into the central nine positions of the input scene, for reasons that are stated below. Putting the objects in the central region of the scene leaves enough space for the objects to remain in the scene after each eye movement. For example, in Figure 8B, the soccer ball object is the attended object in the center of the scene, whereas the motorcycle and cellphone objects are located at the 7 and 14th positions, respectively. Once spatial attention shifts from the soccer ball object to the cellphone object, the position of the soccer ball is shifted from the 13th to the 12th position, and the motorcycle shifts from the 7 to 6th position (Figure 8C). Figure 8D illustrates the shift when the motorcycle is foveated, and the soccer ball and cell phone shift to other positions in the scene.

The second process carries out the view- and positionally-invariant and category learning of the pARTSCAN model. Unlike the input image in the first process, the scenic input contains only one object located in one of the central nine positions to generate different peripheral views of the object. The persistent properties of the view category integrator neurons enable the positionally-sensitive categories (view-specific categories) that are activated by the object in peripheral positions to be associatively linked to the same object category after the object is foveated (see section 4). Section 8.2, summarizes a simulation trial that describes learning driven by three illustrative input scenes which are located from the 7th to the 9th regions (Figures 8E–G) to clarify how ARTSCAN Search cumulatively learns various peripheral views of the same object.

The third process performs a Where’s Waldo search task after positionally-invariant object category learning have previously occurred. Twenty-four objects from the Caltech 101 image data base were selected from 24 distinct categories and each object was presented individually in the central nine regions of the input scene to learn a positionally-invariant category. About 1512 views (24 objects by nine positions by approximately seven eye movements per object) are generated during positionally-invariant object category learning of 24 objects. These object exemplars were compressed through learning to 445 positionally-specific category neurons and, as a result, 24 invariant categories. In addition, during reinforcement learning, the 24 objects were divided into three groups of eight and each group was associated with a different value category to perform many-to-one associations between invariant object categories, value categories, and object-value categories. In all, the 24 invariant object categories were associated with three value categories, 24 object-value categories, and 24 name categories. Each object was simulated on 40 training trials at each position to ensure that learning equilibrated between categorical layers. Although the Fuzzy ART classifier that learns view-specific categories is capable of one trial learning (Carpenter et al., 1991), a slower learning rate between positionally-specific and invariant object categories was used to ensure that, given the vagaries of eye movement search, enough evidence was accumulated to enable sufficiently accurate positionally-invariant object category learning to occur (Cao et al., 2011).

The simulations of Where’s Waldo searches carried out searches via bottom-up, cognitive, and motivational pathways through direct or indirect interactions from the What-to-Where streams to locate Waldo. To create the search scenes, each of the 24 learned objects was placed randomly in a non-foveal position to serve as Waldo. The other eight search scene positions were filled by randomly chosen objects from the other two reinforcement learning groups, so that Waldo was the only object associated with its value category in each scene. The Waldos in these 24 search scenes were then searched bottom-up, cognitively, and motivationally via both direct and indirect pathways, yielding 144 search trials in all. In addition to showing that Waldo could be found in all these cases, search reaction times were also simulated to illustrate the total effect of the number of processing stages that were used to carry out the search trials.

### 8.1. View-invariant object category learning

**Figure 9:**
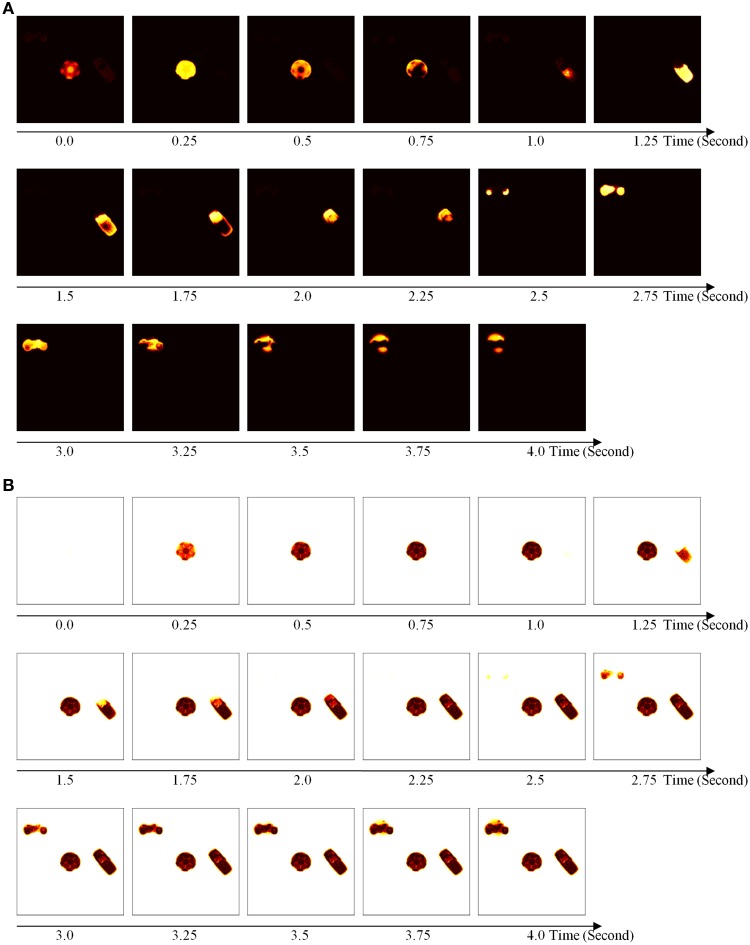
**Temporal dynamics of model simulations in spatial attention map and corresponding habituative transmitter representation**. The input to the simulation contains three objects: a soccer ball, a cellphone, and a motorcycle (see Figure 8B). Each slice represents neural activity at each time step. Darker colors represent lower values. **(A)** Spatial attention map activity in time series when the attentional shroud forms around the attended object. In this case, shroud formation travels from soccer to ocellphone and then to motorcycle. **(B)** Habituative transmitter levels during the times corresponding to **(A)**.

The first simulation shows how view-invariant object categories can be learned within the full ARTSCAN Search architecture. It uses Figure 8B as the scenic input to illustrate the dynamics of how an attentional shroud forms around an attended object, collapses through time, and shifts to another object. In Figure 9A, a shroud forms around a soccer ball, then a cellphone, and finally a motorcycle. Figure 9B shows the level of habituative transmitter gating [Equations (A37, A42)], which is one of the IOR mechanisms that regulates shroud collapse and switching. In particular, shroud collapse induces a reset signal [Equations (A50–A52)] that allows other objects to start to compete for the next attentional shroud. Consistent with the nomenclature proposed by Posner (1980), these shroud changes through time illustrate how attention can be *disengaged*, *move*, and *engaged* by different object surfaces.

**Figure 10:**
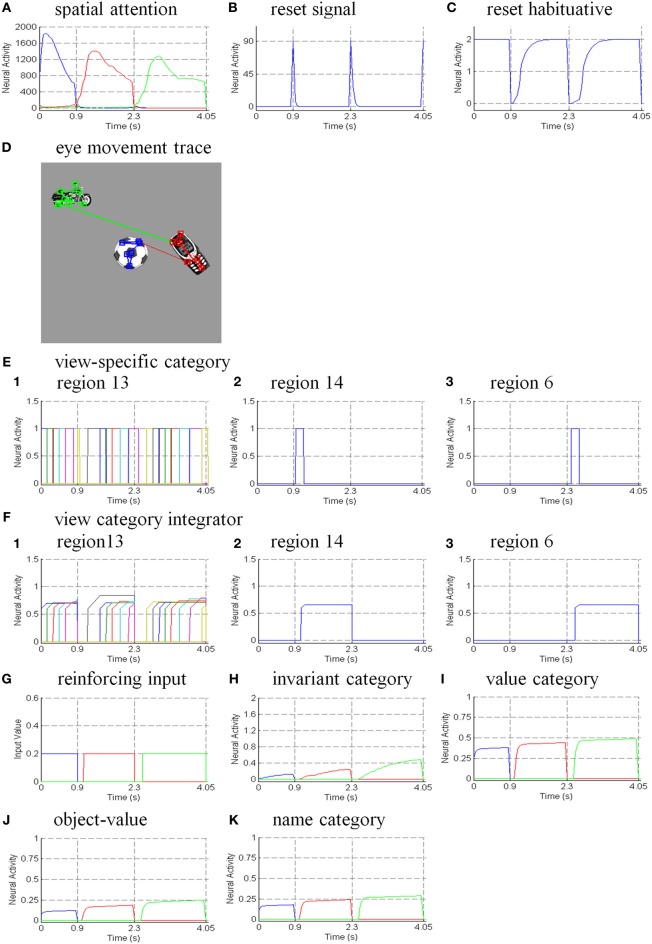
**Model simulations of view-invariant object category learning, after ten reinforcement simulation trials**. Figure 8B presents the scenic input for the simulation. The attentional shrouds competitively form around objects in the Where stream and the winner shroud carries out view-invariant object category learning in the What stream. The persistence of a shroud controls the eye movements on the salient features on the object surface, thereby generating a sequence of views to that are encoded by view-specific categories which are, in turn, associated with the view-invariant object category. The collapse of an active shroud triggers a reset signal which shuts off the corresponding layers, including the spatial attention map, object surface, view category integrator, and view-invariant object category, to enable an attentional shift to another object. **(A)** Sum of the neural activities of each shroud. Each line indicates the total activities of the shroud that is activated by the corresponding object. Blue line: soccer ball; red line: cellphone; green line: motorcycle. **(B)** Object category reset signals. A reset is triggered at time = 1.25, 2.6, and 3.95 when collapse of the shroud reaches the threshold ε for triggering a reset signal in Equation (A55). **(C)** Habituative gate of reset signal. The depletion of the habituative neurotransmitter in Equation (A57) causes the reset signal in Equation (A42) to collapse after its transient burst and then to replenish through time to enable future resets to occur. **(D)** Eye movement traces of the simulated scene. The figures show only the central regions of the simulated scene. The initial eye fixation is located at the center of the scene and each square indicates an eye fixation on the object surfaces. **(E)** View-specific category activities in corresponding regions. Different colored lines indicate that each category activates for a short time and gets reset after the saccadic eye movement occurs. **(1)** Region 13 activation corresponding to the foveal views. **(2)** Region 14 activation corresponding to the extra-foveal view after the first object is learned. **(3)** Region 6 activation corresponding to the extra-foveal view after the second object is learned. **(F)** View category integrator activities in the corresponding regions. Different colored lines indicate integrators’ persistent activities that are inhibited when they receive a reset signal. **(G)** Reinforcing inputs are presented to value categories when the view-invariant object categories are active. **(H)** Invariant object category activities. The activation of the first object category corresponds to learning the cellphone; activation of the third object category corresponds to learning the motorcycle. **(I)** Value category activities corresponding to the activations of invariant object categories. **(J)** Object-value category activities driven by activations of invariant object categories. **(K)** Name category activities.

Figure 10 details the results of view-invariant object category learning of these three objects during reinforcement learning trials. Within a simulation trial, three successive formations and collapses of attentional shrouds in the Where stream (Figure 10A) support learning of three object categories in the What stream. About 24 views are generated (three objects by approximately eight eye movements) leading to learning of the corresponding view-specific categories and activation of the corresponding view category integrator neurons which, in turn, are associated with three view-invariant object category neurons.

A soccer ball is the first object to undergo invariant category learning in the simulation. When the attentional shroud of the soccer ball object (Figure 10A; blue curve) is active in the Where stream, the model spontaneously generates sequences of saccadic eye movements on that soccer ball surface and each eye movement generates a new retinotopic view of the soccer ball for category learning in the What stream. Figure 10D represents all the eye movements and fixations (blue lines and circles) on the soccer ball, cellphone (red lines and circles), and motorcycle (green lines and circles) through time. When a shroud is active, it inhibits the reset neurons, but when the shroud collapses to a threshold level [Equation (A50)], a transient reset signal is activated (Figure 10B).

The reset signal nonspecifically inhibits the spatial attentional and object surface neurons (Figure 7A) and is gated by habituative transmitters (Figure 10C) that help to limit its duration [Equation (A37)]. The more neural activities are decreased by the reset signal, the faster is the reset signal disinhibited and increased, leading to complete inhibition of the currently active shroud and object surface. The transiency of the reset signal allows the objects in the scenic input to compete to form the next attentional shroud, and the habituative transmitters to be replenished during the next surface-shroud resonance (Figure 10C). Because neural transmitters corresponding to the soccer ball object have been depleted, the shroud of the soccer ball loses the next competition, so that other object surfaces can compete to form the next winning shroud. In this simulation, the surface of the cellphone creates the next surface-shroud resonance (Figure 10A; red curve) for invariant object category learning in What stream, and the motorcycle is the last (Figure 10A; green curve).

Each view generated by an eye fixation is represented in retinotopic coordinates and the representations of the attended object will be shifted to the foveal region which is in the center of the scene denoted as region 13. Therefore, when the shroud of the soccer ball is active, several eye movements on the soccer ball generate different boundary representations which activate different view-specific category neurons at region 13 (Figure 10E1). In addition, the view category integrator neuron that is activated by a view-specific category as the eyes explore an object remains active even after its view-specific category gets reset (Figure 10F1), after which a new object can induce its surface-shroud resonance (Figure 10A) and be attended while the eyes explore it and lead to invariant object learning about it. After reset occurs due to the collapse of the soccer ball’s shroud, the cellphone wins spatial attention over the motorcycle and forms the next shroud.

When the cellphone shroud is active and before an eye movement command is generated toward the attended cellphone, the view-specific category neuron and the view category integrator neuron is activated in response to the peripheral view of the cellphone at the 14th region (Figures 10E2,F2). After the eye fixation is on the cellphone surface, it brings the cellphone into the foveal region 13 (Figure 8C) leading saccadic eye movements to start to explore the features on the cellphone. These explorations create a series of foveal views at region 13 which trigger invariant object category learning of cellphone until the reset occurs again (Figure 10E1).

The same explanation holds for the motorcycle. Once the collapse of the cellphone shroud triggers reset and before the motorcycle is foveated, a view-specific category and view category integrator neurons are activated in response to a peripheral view of the motorcycle in the 6th region (Figures 10E3,F3). Then the motorcycle is shifted from the retina periphery to the fovea at 13th region (Figure 8D) to perform view-invariant motorcycle object category learning.

The persistent activations of view category integrator neurons throughout the search of each object help to keep the emerging invariant object category active (Figure 10H) after the first view category integrator activates it, after which multiple view category integrator neurons can be associated with it.

**Figure 11:**
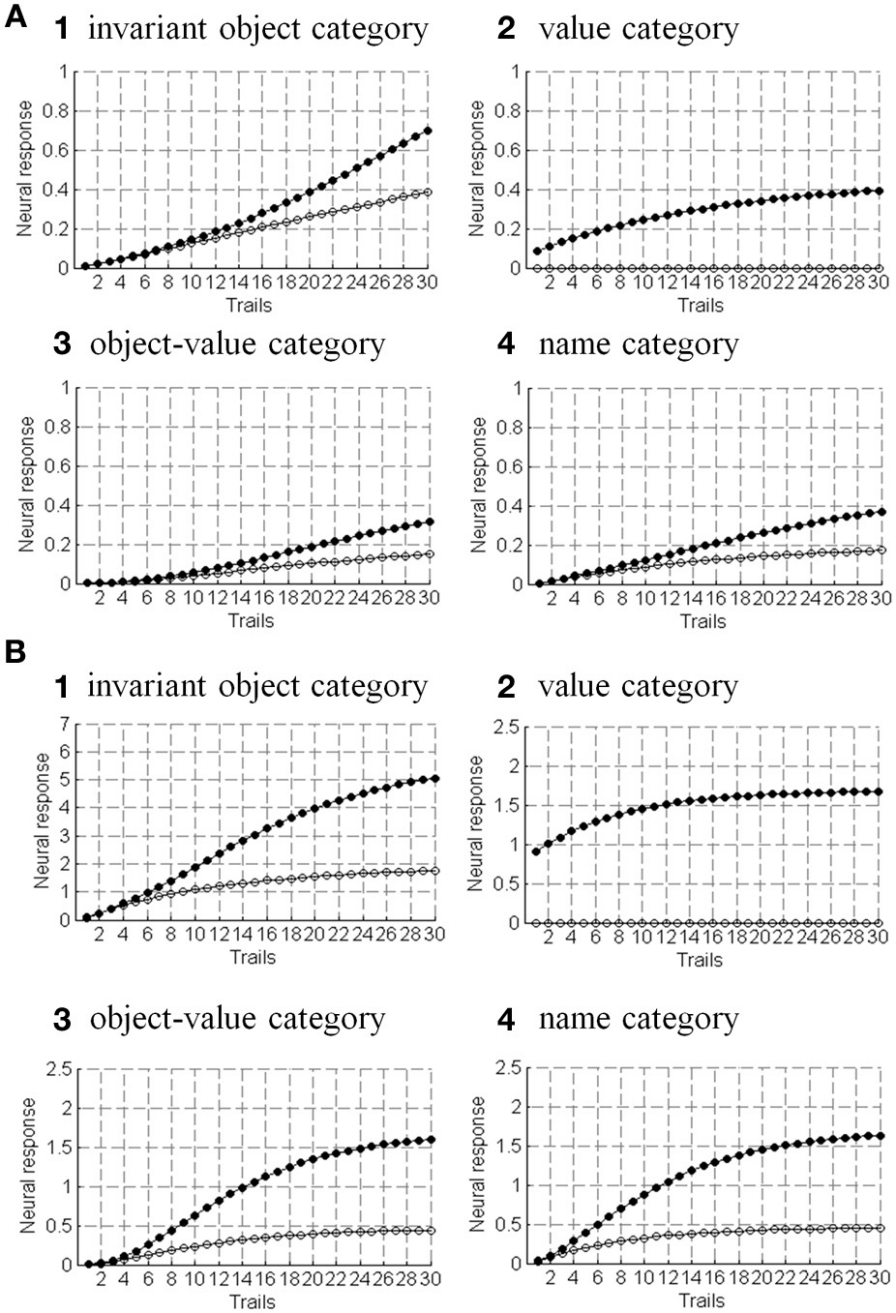
**(A)** Trial-by-trial category activities during view-invariant object category learning. Category learning activities are shown both without (open circles) and with (solid circles) simultaneous reinforcement learning. Each condition involves 30 trials with each trial processing a simulated scene with an average duration of 4.05 s, corresponding to Figures 8B, 10. Data points represent the average activity levels during cellphone learning. Other objects generate similar learning curves. **(1)** Average view-invariant object category responses. **(2)** Average value category responses. **(3)** Average object-value category responses. **(4)** Average name category responses. **(B)** Trial-by-trial changes in positional- and view-invariant object category learning. Each trial processes three consecutive simulated scenes, each with an average duration of 3.95 s, corresponding to Figures 8E–G, 12. The curves are analogous to those in **(A)**.

Association of an active invariant object category with a reinforcer-activated (Figure 10G) drive representation triggers conditioned reinforcer learning and incentive motivational learning processes (Figure 4) that lead to enhanced activations of value category neurons (Figure 10I) and object-value category neurons (Figure 10J) through ITa-AMYG-ORB resonances, and enhanced activations of the corresponding name category neurons (Figure 10K). To distinguish the effects of reinforcement learning, the ITa-AMYG-ORB resonances are shut off by fixing the adaptive weights from the invariant categories to the value categories and from the object-value categories to invariant categories to equal zero before reinforcement simulation trials. This simulation demonstrates that the model is capable of performing category learning in the absence of reinforcement learning. Figure 11 compares the neural responses, when the cellphone’s shroud is active, across thirty trials with (solid circles) and without (open circles) reinforcement learning, thereby showing how reinforcement learning enhances cell activations.

For the soccer ball and motorcycle, the neural responses of the categorical stages are similar to those activated by the cellphone because the model performs category learning of individual objects through time. Before reinforcement learning, the neural responses of the value category stay at the rest level due to the absence of learned associations from the invariant object categories (Figure 11A2; open circles). After reinforcement learning, the responses of view-invariant categories (Figure 11A1), value categories (Figure 11A2), object-value categories (Figure 11A3), and name categories (Figure 11A4) show enhanced activations relative to their values in the absence of reinforcement learning.

### 8.2. Positionally-invariant object category learning

**Figure 12:**
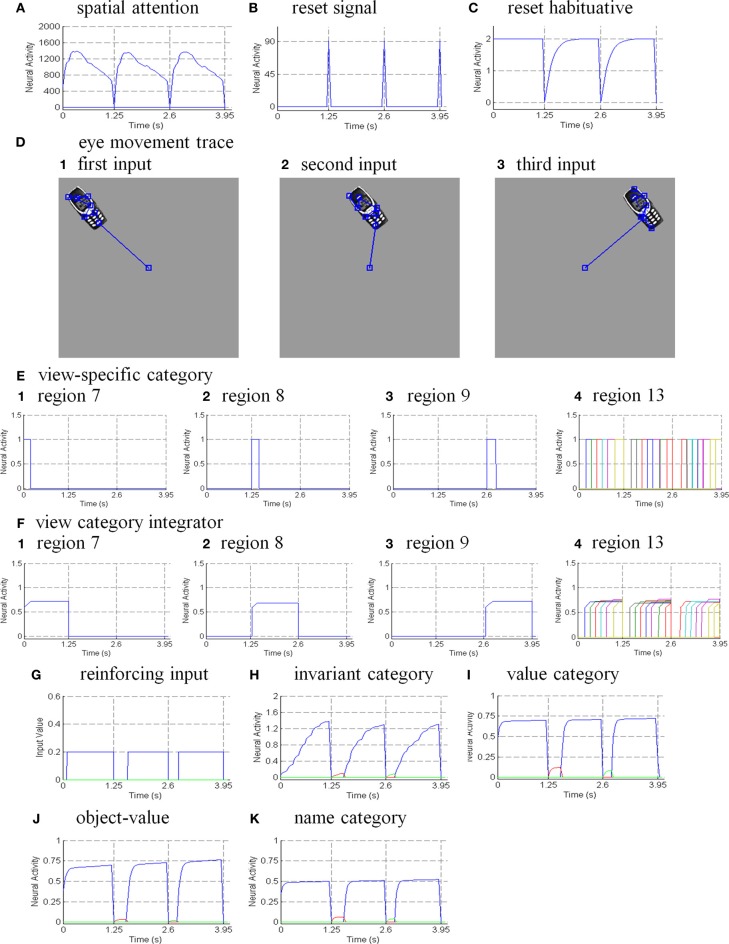
**Model simulations of positionally- and view-invariant cellphone object category learning, after 10 reinforcement simulation trials**. Model receives a sequence of three simulated scenes. Each scene contains a single cellphone placed at different positions (see Figures 8E–G), and the initial eye fixation is located at the center of the scene. Before the object is brought to the foveal region by a saccadic eye movement, a view from the retinal periphery is generated to activate the view-specific category in the What stream and the subsequent categorical stages. An attentional shroud forms around the cellphone in the Where stream and controls the eye movements visiting several salient features on the cellphone surface which generate a sequence of views to the What stream during shroud persistence. After the collapse of an attentive shroud triggers a reset to inhibit the spatial attention map, object surface map, view category integrator neurons, and view-invariant object category neurons, another simulated scene is fed to the model to repeat category learning until all the scenes are learned. **(A)** Sum of the neural activities in three attentional shrouds which are active at times 0–1.25, 1.25–2.6, and 2.6–3.95 s. **(B)** Object category reset signals occur at times 1.25, 2.6, and 3.95 s when shroud collapse reaches the reset threshold. **(C)** Habituative gate of reset signal. **(D)** Eye movement traces scanning the cellphone presented in three positions. **(E)** View-specific category activities of the corresponding regions. Different colored lines indicate that each category activates for the duration of an eye fixation and gets reset after the saccadic eye movement occurs. **(1)** neural activation corresponding to the extra-foveal view of the first cellphone input at region 7. **(2)** activation corresponding to the extra-foveal view of the second input at region 8. **(3)** activation corresponding to the extra-foveal view of the third input at region 9. **(4)** activation corresponding to the foveal views of all the scenes at the foveal region 13. **(F)** View category integrator neuron activities in corresponding regions. **(G)** Reinforcing inputs **(H)** Invariant object category neuron activities. From *t* = 0–1.25 s, the invariant category is activated via a series of activations from view category integrators until it receives a reset signal. Another invariant category neuron (red line) is activated corresponding to the beginning of the second scene’s category learning and then is inhibited by the previously learned invariant category which is activated by a previous view-specific category when a feature on the cellphone is repeatedly selected. The activation of the other invariant category (green line) corresponds to the beginning of third scene’s category learning and is inhibited by the first learned invariant object category when a previously learned view-specific category is activated. **(I)** Value category activities corresponding to the activations of invariant object categories. **(J)** Object-value category activities corresponding to the activations of invariant object categories. **(K)** Name activities corresponding to activations of object-value categories.

This section uses the ARTSCAN Search model to simulate both view- and positionally-invariant category learning. Positionally-invariant category learning was carried out for all 24 objects in all nine positions. To illustrate how the network behaves, Figure 12 shows a simulation in which three consecutive input scenes generate a series of shrouds through time. Each shroud controls a sequence of 7–8 explorations of the positions of salient features, or hot spots, by eye movements on the object surface and each eye fixation provides either an extra-foveal or a foveal view for category learning. About 21 views are hereby generated (three objects by approximately seven eye movements) during the simulation trial learning of three cellphone exemplars. Because features on an attended object surface that are selected by eye movements can be repeatedly chosen when learning of same object at different positions, the same features can activate the previously learned view-specific category. As a result, these views are compressed to 16 view-specific categories through learning, and all the activated view category integrator neurons are associated with the same invariant object category, value category, object-value category, and name category neurons.

Figure 12 illustrates this process by starting with the cellphone as the scenic input in the 7, 8, and 9th regions. The following sequence of events occurs through time during learning of the cellphone’s positionally-invariant category. When the cellphone begins in position 7, a cellphone surface-shroud resonance forms. The persistence of the shroud (Figure 12A, first blue curve) enables saccadic eye movements to move from the center of the scene and explore several hotspots on the cellphone surface (Figure 12D1) while object category learning continues until the shroud collapses, thereby triggering category reset signals (Figure 12B). The reset signals shut off the spatial attention map and object surface representations, and inhibit the invariant object category in the What stream. The transient burst of the reset signal leads to depletion and replenishment of its gated habituative transmitters through time (Figure 12C).

Cellphone learning proceeds as follows: Initially, one view-specific category in region seven gets activated in response to the extra-foveal view of the cellphone (Figure 12E1) and, in turn, activates the corresponding view category integrator neuron (Figure 12F1) which remains active and is associated with the corresponding invariant object category neuron (Figure 12H). After the first saccadic eye movement command is computed by the eye movement map, the cellphone is shifted from the periphery to the foveal region (region 13). The persistence of the shroud enhances the surface representation and its surface contours, whose selection controls eye movements that explore salient features on the surface, thereby activating a sequence of foveal views and the corresponding sequence of view-specific category neurons (Figure 12E4) and their view category integrator neurons (Figure 12F4). View category integrator neurons persist during the active shroud even after the corresponding view-specific category neurons get reset. Because the view-invariant object category neuron is active before the object is foveated, these persistent properties of view category integrator neurons help both extra-foveal and foveal views to be associated with the emerging invariant object category.

Reinforcement learning pairs activations of the emerging invariant object category with a sequence of external reinforcing inputs (Figure 12G). It hereby converts the active invariant object category into a conditioned reinforcer and source of incentive motivation by strengthening associative links from the category to the value category, and from the value category to the object-value category, respectively. In all, the corresponding ITa-AMYG-ORB resonances lead to enhanced activities of invariant categories (Figure 12H), value categories (Figure 12I) and object-value categories (Figure 12J), which influence the activations of the name categories (Figure 12K).

The collapse of the cellphone’s shroud in region 7 results in category reset at the view category integrator and view-invariant object category layers [Equations (A61, A63)] as well as a complete inhibition of activity across the spatial attention and object surface layers. After the reset occurs, another simulated scene with the cellphone in position 8 as in Figures 12F,D2 is fed into model to repeat the learning processes. As explained above, the initial eye fixation is located at the center of the scene, so the cellphone generates an extra-foveal view to the What stream where a view-specific category neuron in region 8 gets activated (Figure 12E2), which activates the corresponding view category integrator neuron (Figure 12F2), which persists and learns to be associated with a new invariant object category neuron (Figure 12H, red curve) and the subsequent categorical layers. After a saccadic eye movement is generated to bring the cellphone into the foveal region (region 13), the active shroud of the cellphone in region 8 enables eye movement explorations to occur on the cellphone surface and thus generate a sequence of foveal views that initiate new view-specific category learning and view integrator activations (Figures 12E4,F4).

However, as noted in section 2, how the eyes choose the next saccadic target is not random. Surface contour signals are selected to ensure that the eye movements select the salient features on the attended object’s surface (Figure 12D2). The features that are selected in the simulated scene of cellphone at region 7 are thus chosen again when learning the cellphone located in the region 8. That is, at least one previously learned view-specific category neuron is activated in turn activates the corresponding view category integrator. This integrator learned to be associated with the previously learned invariant object category. Due to the persistent activities of view category integrator neurons, the view category integrator neuron which is activated by the extra-foveal view in region 8 can be associated through learning with the previously learned invariant object category (Figures 3, 12H, second blue curve). As the result, the extra-foveal views of the cellphone (regions 7 and 8) are linked to the same invariant object category, thereby developing its positionally-invariant property.

After reset occurs due to collapse of the shroud of the cellphone in region 8, a simulated input containing only one cellphone object in the 9th region (Figures 8G, 12D3) is fed into the system to extend the positional invariance of the emerging object category. Before the cellphone is shifted into the foveal region by a saccadic eye movement, a view from the retinal periphery is generated and activates the view-specific category neuron in region 9 (Figure 12E3) and the corresponding view category integrator neuron (Figure 12F3) that activates a new invariant object category neuron (Figure 12H, green curve). By the same process that was explained above, the view category integrator neuron can learn to be associated with the previously learned -invariant object category that is activated by a view category integrator neuron after a feature on the cellphone surface is repeatedly selected (Figure 12D3). The same processes take place for objects appearing at other extra-foveal positions. As a result, ARTSCAN Search can perform positionally-invariant object category learning from multiple initial object positions.

Figure 11B shows the development of model responses across learning trials, with and without reinforcement learning. The model requires approximately 30–40 trials before the associative weights become asymptotically stable. Category learning without reinforcement learning eliminates the ITa-AMYG-ORB resonances by setting the weights from invariant object categories to value categories to zero. As a result, responses of the value category remain zero (Figure 11B2, open circles), and responses of the invariant category (Figure 11B1), object-value category (Figure 11B3), and name category (Figure 11B4) show smaller increments compared to those during reinforcement learning trials.

To carry out the reinforcement learning trials, it was assumed that the 24 objects that were conditioned were associated with one of three value categories. For definiteness (although this has no effect on the simulations), each value category was associated with 8 of the 24 objects. When the first object was associated with its value category, there was no effect of other objects because their initial conditioned reinforcer and incentive motivational weights were chosen equal to zero. Consider learning trials with the second object that is associated with a given value category. When the value category gets activated, it can send incentive motivational signals to the object-value category of the first object to be conditioned. However, as shown in Equation (A75), these conditioned signals are modulatory. Since the first object is not present, its invariant object category is inactive, and thus its object-value category does not receive an input from the object category. As a result, the object-value category of the first object remains inactive. This is also true for all objects that were associated with a given value category when a different object is presented.

### 8.3. Top-down primed search to waldo discovery

Top-down search tasks are based on the view- and positionally-invariant object category learning of 24 objects, described in section 8.2, after the learned weights between categorical layers have equilibrated. The top-down primed search can be triggered either via a name category neuron in PFC by receiving a priming name input (Figures 6A,B) or via a value category in AMYG by receiving sufficiently large internal motivational drive signal (Figures 6C,D). Either way, the corresponding object-value category in ORB can be activated and projects to the invariant object category in ITa. The amplified invariant object category top-down primes multiple learned view-specific category neurons in ITp through view category integrator neurons. During the primed search processes, the object-value categories, the invariant object categories, and view category integrators receive volition control signals from the BG to ensure the top-down prime to be appropriately activated. Bottom-up inputs from the objects in the viewed scene also activate the view-specific category neurons in ITp. The view-specific category with the best combination of top-down prime and bottom-up input will be mostly highly activated. This enables a winner-take all choice of the primed view-specific category, using the choice mechanism that was summarized in section 7.3.7. The selected view-specific category can induce eye movements toward the target object either via a direct or an indirect pathway. For the scenes simulated in this article, ARTSCAN Search achieves 100% performance accuracy of correctly finding Waldo. In response to realistic scenes, many factors may reduce performance accuracy, including distractors, internal noise, speed-accuracy tradeoffs, imperfections of figure-ground separation, and the like.

Another important factor that can limit search accuracy in the brain is the cortical magnification factor. As noted in section 9.2 pARTSCAN, and ARTSCAN Search enable positionally-invariant category learning to occur with no loss of acuity for peripherally detected objects. The high peripheral acuity is due to the fact that, for simplicity, these models do not incorporate the cortical magnification factor, which would cause object representations that are processed from extra-foveal positions to have coarse sensory representations. If several objects in a scene are featurally similar, their peripheral representations could then be associated with more than one similar object in foveal view, and thus would not unambiguously predict a definite object category. Rather, they may only predict a coarser and more abstract category. However, once these objects are foveated, they benefit from the higher resolution of foveal processing.

**Figure 13:**
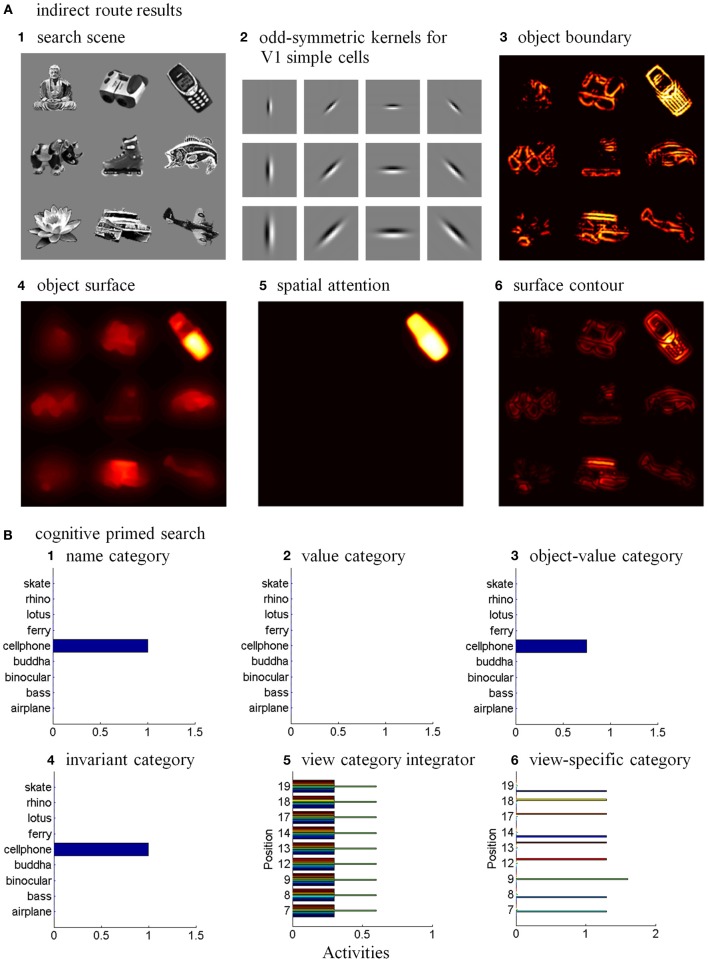
**Where’s Waldo cognitive primed search results**. Search is based on positionally-and view-invariant object category learning of 24 objects, as illustrated in **(A)**. In **(B)**, a cognitive primed search are illustrated. **(A)** In the indirect route, the amplified view-specific category selectively primes the target boundary to make it stronger than other object boundaries in the search scene. **(1)** A typical input for the search task with the cellphone denoted as the Waldo target. **(2)** Odd-symmetric kernels for V1 polarity-sensitive oriented simple cells. The kernels have four orientations and three scales. **(3)** The boundary representation gates the filling-in process of the object surface stage. Priming from the cellphone’s view-specific category increases the contrast of its target surface. **(4)**. The enhanced cellphone surface representation competitively forms the cellphone’s attentional shroud **(5)** within the spatial attention map. This shroud draws spatial attention to the primed cellphone object. The hot spots on the cellphone’s enhanced surface contour **(6)** determine eye movements to salient features on the cellphone. **(B)** Cognitive primed search. The category representations in a top-down cognitive primed search are consistent with the interactions in Figures 6A,B. The bars represent category activities at the time when the view-specific category is selectively amplified through the matching process. **(1)** Name category. Only the cellphone category receives a cognitive priming signal. **(2)** Value category. The value category remains at rest because no reinforcement signals are received. **(3)** Object-value category. The object-value category corresponding to the cellphone is primed by the cellphone name category. The object-value category also receives a volitional signal (Figure 1B), which enables its top-down prime to activate suprathreshold output signals. A volitional signal also reaches the invariant object category and view category integrator stages to enable them to also fire in response to their top-down primes, as now discussed: **(4)** Invariant object category. The cellphone invariant object category fires in response to its object-value category and volitional inputs. **(5)** View category integrator. The view category integrators corresponding to the cellphone also fire in response to their invariant object category and volitional inputs. Colored bars in each position index activations corresponding to the different objects. View category integrators at each position that learn to be associated with the cellphone’s invariant object category have enhanced representations. **(6)** View-specific category. The view-specific category at position 9 receives a top-down priming input from its view category integrator and a bottom-up input from the cellphone stimulus. It is thereby selectively amplified.

**Figure 14:**
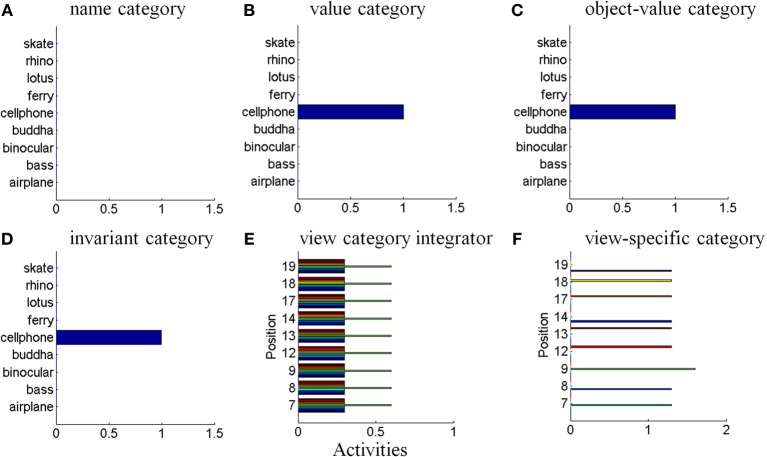
**Where’s Waldo motivational drive search results**. The category representations during a motivational drive search are consistent with the interactions in Figures 6C,D. The value category that was associated with the cellphone receives an internal motivational priming input that activates a motivational signal to the object-value category which, supplemented by a volitional signal, amplifies the corresponding invariant object category through an inferotemporal-amygdala-orbitofrontal resonance. The various results are analogous to those in Figure 13B.

Figures 13, 14 summarize model simulations of the cognitively primed search (Figure 13) and a motivational drive search (Figure 14). A search scene is composed of nine different learned objects at the central nine positions within a 5 × 5 = 25 position scene. Figure 13A is an exemplar of a search scene in which the cellphone object is denoted as Waldo.

In the simulation of a cognitively primed search that is summarized in Figure 13B, the name category neuron corresponding to the cellphone receives a priming signal (Figure 13B1) and then projects to the object-value category. The active object-value category (Figure 13B3) continually excites the corresponding invariant object category (Figure 13B4). To show the effect of a purely cognitive prime, it is assumed that the value categories are not active. In the simulation, this happens because the value categories do not receive any internal drive inputs, and thus their activities remain at the rest level (Figure 13B2). The active invariant object category, supplemented by volitional signals, top-down primes all the view- and positionally-specific categories through the view category integrator neurons. The view category integrators corresponding to different positions receive both top-down primes from the invariant object categories and volitional signals from the BG. As a result, all the view- and positionally-specific categories that were associated with cellphone object category get amplified (Figure 13B5). The view-specific category with the matched position from the bottom-up Waldo input gets the most activation (Figure 13B6); that is, the category that encodes the extra-foveal view of cellphone at the 9th position.

For the motivational drive search mechanism (Figure 14), the value category corresponding to the cellphone receives an internal drive input (Figure 14B) that triggers an incentive motivational signal to the object-value category. To distinguish the effect of motivational drive search from the cognitive primed search, the connections from the object-value categories to name categories are eliminated so that the name category neurons stay at their rest level (Figure 14A). As noted in section 7.3.7, the competitive dynamics of the model enable the active object-value category (Figure 14C) to top-down excite the corresponding invariant object category. As in the top-down cognitive primed search, the enhanced invariant object category (Figure 14D) top-down primes all the view category integrators (Figure 14E) and, in turn, its view-specific category. This prime can now amplify the most active view-specific category, which corresponds to the extra-foveal cellphone view at the 9th position, (Figure 14F).

The selected view-specific category neuron in ITp induces an eye movement to the Waldo target through either a direct or an indirect route. The direct route from the view-specific category layer to the eye movement map via a learned adaptive weight can more quickly elicit a saccadic eye movement. The learning between a view-specific category and the eye movement map occurs during positionally-invariant category learning when a non-foveal object learns to activate its view-specific category and generates an eye movement command to move the eyes to its position. Then both the view-specific category and the representation of the object’s extra-foveal position are active, so that an association between them can be learned.

This direct search route can be triggered by either the cognitive primed search pathway (Figure 6A) or the motivational drive search pathway (Figure 6C). However, along the indirect route, the selected view-specific category neuron selectively primes its target boundary representation (Figure 13A3) which gates the surface filling-in process to increase the contrast of the selected target surface (Figure 13A4). Spatial attention corresponding to the target surface competitively wins to form an attentional shroud through a surface-shroud resonance (Figure 13A5). As a result, the surface contour (Figure 13A6) of the attended surface gets strengthened, leading to selection of its hot spots as eye movement targets.

**Figure 15:**
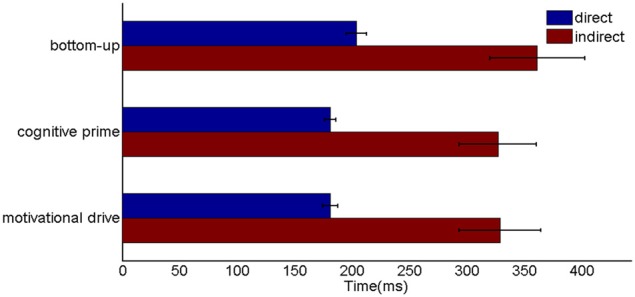
**Search reaction times under different search conditions**. The search reaction times are statistically computed in the eye movement map via bottom-up, cognitive primed, and motivational drive search mechanisms through a direct and an indirect route. Blue bars correspond to the direct route and red bars indicate the indirect route. The slowest RTs are in the bottom-up pathway via the indirect route (375 ± 50 ms). The simulation reaction times of the cognitive primed pathway (335 ± 40 ms) and motivational drive pathway via the indirect route (335 ± 45 ms) are similar. The RTs via the direct route are: bottom-up pathway (200 ± 10 ms), cognitive primed pathway (180 ± 5 ms), and motivational drive pathway (180 ± 5 ms), respectively. See the text for further discussion.

Figure 15 shows the search reaction times across search trials. For example, the cellphone object in Figure 13A is set as a Waldo target and is simulated under different search pathways via either the direct or indirect route until Waldo is foveated. The bottom-up search pathway has longer search reaction times compared to the top-down cognitive primed and the motivational drive pathways. This is because the bottom-up pathways require more processing stage interactions (see Figure 5) to locate the target. In addition, the reaction time in the direct pathway is always shorter than in the indirect pathway because the indirect pathway has more stage interactions to compute the saccadic eye movement. The search reaction times of the direct route in each search mechanism are similar because the eye movement is activated via the learned pathway from the selected view-specific category and the interactions between categorical layers are the same, whereas the search reaction times in the indirect route are different for different targets due to the different surface contour strength of the various objects.

The indirect path reaction times between 275 and 375 ms are comparable to, say, the reaction times in the Brown and Denney (2007) experiments on spatial attention shifts, which are quantitatively simulated in Foley et al. (2012) using the dARTSCAN model.

## 9. Discussion and related models

The ARTSCAN Search model builds upon the ARTSCAN model (Fazl et al., 2009) and its further development in pARTSCAN to enable both view- and positionally-invariant object categories to be learned (Cao et al., 2011). The model introduces several major additional improvements and innovations. First, incorporating positionally-invariant object category learning is necessary to perform the different search tasks, which all show how object attention in the What stream can activate spatial attention in the Where stream. The model thus incorporates multiple bi-directional connections between two cortical streams: from the Where stream to the What stream to perform both view- and positionally-sensitive and view- and positionally-invariant category learning, and from the What stream to the Where stream to perform either bottom-up or top-down primed searches. Second, volitional signals from the BG are needed to convert top-down priming signals into suprathreshold activations during search tasks. Third, during category learning in the What stream, cognitive-emotional resonances can strengthen object category, value category, object-value category, and name representations to enable valued objects to preferentially compete for object attention during search tasks. Fourth, all these processes, taken together, can support performance of bottom-up or top-down cognitive or motivational, direct or indirect pathway, Waldo searches. During the top-down searches, a primed object name, or distinctive motivational source in the What stream can interact with the Where stream to direct spatial attention and eye movements to the position of the object.

### 9.1. Spatial vs. object attention

The ARTSCAN Search model explicates neural processes that have been described in many psychological experiments and models. A large number of visual search experiments and models consider top-down priming, and how it may interact with parallel visual representations of target features (Wolfe et al., 1989; Wolfe, 1994; Itti and Koch, 2001; Müller et al., 2003), by building on feature integration theory (Treisman and Gelade, 1980) to bias spatial selection of target positions. Feature dimensions, such as color, intensity, shape, size, orientation, etc., are combined into a saliency map that enables bottom-up information to attract an observer’s attention, whereas expectancies introduce top-down constraints. Attention can be shifted to an object or a location through a combination of bottom-up and top-down processing.

The Guided Search (Wolfe, 1994) and Saliency Map models (Itti and Koch, 2001) rely on spatial competition to select the most salient feature. Unlike the ARTSCAN Search, pARTSCAN, and ARTSCAN models, these alternative models are all pixel-based, rather than object-based, models. Observers detect whether a single feature object was present or not during visual search experiments; there was no need to identify the target. These models thus do not include object-based attention or any of the other concepts and mechanisms that are needed to learn object categories and object-based searches, and cannot explain the corresponding data bases. The ARTSCAN Search model, in contrast, provides a detailed description of how spatial and object attention, invariant object category learning, predictive remapping, eye movement search, and conscious visual perception and recognition are intimately linked. In particular, the surface-shroud resonance that is predicted to correspond to paying focal spatial attention to an object and to regulate invariant object learning and eye movement search, has also been predicted to be the event that triggers conscious perception of visual qualia (Foley et al., 2012; Grossberg, 2012, 2013a).

Other models have focused on object recognition, rather than visual search *per se*. Riesenhuber and Poggio (2000) proposed a hierarchical model called HMAX to illustrate how view-invariant object recognition occurs. The HMAX model is a feedforward network that generates a sparse representation of the input to achieve its categorizations by incorporating properties of earlier models such as the Neocognitron (Fukushima, 1980, 1986) and VIEWNET (Bradski and Grossberg, 1995) models. The view-tuned units at the model’s lower stages, which are tuned to same features of the object but at different scales, rotations, and illumination, gradually and in parallel increase feature complexity and receptive field size at the higher stages. The view-invariant units at the higher stages are achieved by pooling together the appropriate view-tuned units for each object. The HMAX model differs from ARTSCAN Search in multiple ways. Most notably, ARTSCAN Search is not a feedforward model and does not depend upon generating a sparse representation of the input. Instead, ARTSCAN Search includes both bottom-up and top-down interactions, as well as recurrent interactions at multiple processing stages, to carry out its attentional, learned categorization, and search properties. In particular, in HMAX there is no spatial or object attention, or coordination of the What and Where cortical streams to learn invariant object categories and to drive object searches. Moreover, ARTSCAN Search incorporates ART dynamics to learn view-specific object categories that can be chosen from a dense, non-stationary input environment, without a loss of learning speed or stability (Carpenter and Grossberg, 1987, 1991; Carpenter et al., 1991). Feedforward categorization models fall apart under such learning conditions (Grossberg, 1988).

Kanan and Cottrell (2010) have developed a model to classify objects, faces, and flowers using natural image statistics. Their preprocessing tries to emulate luminance adaptation within individual phororeceptors. To do this, they compute the logarithm of each pixel intensity and then normalize the result. The logarithm compresses the dynamic range of the image, but has unbounded limiting values at high and low arguments, so cannot be the correct form factor for biological preprocessing. ARTSCAN Search does not try to model individual photoreceptors, although its front end can be augmented by detailed models of vertebrate photoreceptor adaptation. These models show how an intracellular shift property and Weber law can be achieved using habituative transmitter gates that normalize photoreceptor response and quantitatively fit photoreceptor psychophysical and neurophysiological data (Carpenter and Grossberg, 1981; Grossberg and Hong, 2006). Instead, ARTSCAN Search embodies the next stages of visual brain adaptation using a shunting on-center off-surround network that computes a regional contrast normalization which also exhibits the shift and Weber law properties (e.g., Grossberg, 1983, sections 21 and 23; Werblin, 1971; Grossberg and Hong, 2006). See Equation (A6). Kanan and Cottrell then use principal component analysis (PCA) to learn filters that play the role of simple cells. They discard the largest principal component, and then select *d* of the remaining components by optimizing performance on an external dataset. These useful, but computationally non-local, computer vision operations do not seem to have biological homologs. ARTSCAN Search does not learn its simple and complex cell filters [see Equations (9–17)], but these filters are similar to the oriented filters that self-organize in response to image statistics in biological self-organizing map models of cortical development (e.g., Olson and Grossberg, 1998). Kanan and Cottrell compute a saliency map from their filters using a number of other non-local operations, and their fixations are chosen randomly. In contrast, in ARTSCAN Search, the salient features that are computed from the surface contours of the attended surface generate predictive eye movement commands to fixate the positions of these salient features, until the surface-shroud resonance collapses, and enables another surface to be attended and searched [see Equations (43–49)]. Random fixations do not allow the autonomous learning of invariant object categories, and do not occur *in vivo* (Theeuwes et al., 2010). Kanan and Cottrell apply PCA to the collected feature vectors, and the 500 components with the largest eigenvalues are selected and normalized. This information is combined by assuming fixations are statistically independent. After T fixations, the class with the greatest posterior is assigned. In contrast, ARTSCAN Search can carry out incremental unsupervised or supervised learning of both view- and positionally-specific categories and view- and positionally-invariant categories using an ART classifier whose top-down expectations both dynamically stabilize the learning of multiple categories and provide the pathways for carrying out top-down Where’s Waldo searches [see Equations (53–65)].

Grossberg et al. (1994) proposed in their algorithmic Spatial-Object Search, or SOS, model how spatial attention and object attention interact with visual boundary and surface representations to direct visual search. The ARTSCAN, pARTSCAN, and ARTSCAN Search model greatly expand this framework to a dynamical neural theory which proposes how identified cortical cells in multiple regions of the What and Where streams may achieve invariant object category learning and Where’s Waldo searches.

Another extension of this framework is the ARTSCENE Search model (Huang and Grossberg, 2010) which proposed how contextually-cued search may occur (e.g., Chun and Jiang, 1998) by accumulating and categorizing sequential spatial and object contextual information via the parahippocampal and perirhinal cortices, interacting with parietal, inferotemporal, and prefrontal cortices, to direct a search based on spatial and object contextual evidence, respectively. For example, after seeing a stove and a sink, one expects to see a refrigerator more than a beach. This kind of evidence accumulation is not modeled in ARTSCAN Search and needs to be added to a future extension of the model.

### 9.2. Central vs. peripheral vision

Due to the coarse resolution of peripheral vision, high-acuity object recognition requires a combination of selective attention and successive eye movements that bring the objects of interest into foveal vision (Liversedge and Findlay, 2000). In contrast, Thorpe et al. (2001) performed an experiment in which natural images are flashed at the retinal periphery. Human subjects are asked to respond if a natural image contains an animal. The results showed that, even in the absence of foveating eye movements, visual information initiating in the retinal periphery can be processed to make superordinate categorizations, such as deciding whether or not an animal is contained in the scene. However, the subjects failed to identify the animals that they detected in the image. To identify a tiger as a tiger (rather than as an animal), objects require a more detailed analysis by foveally-mediated perceptual and categorization processes. Although pARTSCAN and ARTSCAN Search enable positionally-invariant category learning to occur, object representations that are processed from extra-foveal positions *in vivo* have coarse sensory representations due to the cortical magnification factor. If several objects in a scene are featurally similar, they can be associated with multiple similar objects in foveal view, and thus do not unambiguously predict a definite object category. Rather, they may only predict a coarser and more abstract category. However, once these objects are foveated, they benefit from the higher resolution of foveal processing. The current model does not simulate the cortical magnification factor, for simplicity, since its focus is on higher-level processes. View-invariant category learning has, however, been demonstrated using log-polar preprocessing to represent the cortical magnification factor and Fuzzy ARTMAP as the view-specific category classifier (Bradski and Grossberg, 1995; Fazl et al., 2009). These results show that including the cortical magnification factor can be successfully incorporated in a future version of the model.

### 9.3. Top-down processes: three mechanisms

Top-down processes occur in both cortical streams. For the Where cortical stream, it has been suggested that top-down attention can guide target selections by facilitating information processing of stimuli at an attended location (Wolfe, 1994; Hyle et al., 2002; Kristjánsson et al., 2002; Müller et al., 2003). Such top-down modulation can enhance the effective contrast of an attended stimulus (Carrasco et al., 2000; Reynolds and Chelazzi, 2004). Fazl et al. (2009) proposed how a surface-shroud resonance can enhance the contrast of an attended stimulus as part of the process whereby the Where stream pays focal attention to an object and modulates the learning of view-invariant object categories. The ARTSCAN Search model extends this insight to the learning of view- and positionally-invariant object categories and the capacity to carry out bottom-up and top-down searches.

For the What cortical stream, Bar (2003) proposed that low spatial frequencies in the image rapidly project to PFC through magnocellular pathways. PFC can then project back to inferotemporal cortex and to amygdala through orbitofrontal cortex. In particular, activity in the orbitofrontal cortex is involved in producing of expectations that facilitate object recognition (Bechara et al., 1996; Frith and Dolan, 1997; Bischoff-Grethe et al., 2000; Carlsson et al., 2000; Petrides et al., 2002). ARTSCAN Search, and its precursors in the CogEM, MOTIVATOR, and START models, simulate how the activation of IT is capable of learning a cognitive-emotional ITa-AMYG-ORB resonance that supports motivated attention to top-down enhance an object category representation and thus facilitate its recognition (Grossberg, 1975; Grossberg and Levine, 1987; Grossberg and Merrill, 1992; Grossberg and Seidman, 2006; Dranias et al., 2008). ARTSCAN Search further clarifies how a cognitively-mediated search that engages PFC, and a motivationally-mediated search that engages AMYG, can utilize these circuits.

A third and related mechanism drives a top-down primed search process using knowledge about the learned objects (e.g., finding Waldo), with PFC as major source of inputs to IT (Miller et al., 1996; Cavada et al., 2000). Bar (2003) also emphasized a top-down mechanism for facilitation of object recognition from prefrontal region to the IT area via expectancies from the orbitofrontal cortex. The ARTSCAN Search model, and its CogEM, MOTIVATOR, and START precursors, also clarifies the role of ORB in mediating object-value categories that are enhanced when objects are emotionally salient and can then be selectively attended through motivated attention during a primed search task (Rolls, 1999, 2000; Baxter et al., 2000; Schoenbaum et al., 2003; Pessoa and Ungerleider, 2004).

### 9.4. Model extensions

The present model carries out all of its computations in Cartesian coordinates. Future versions of the model that wish to include the compression and other representational properties of space-variant processing can preprocess the input images using the cortical magnification factor (Schwartz, 1980; Seibert and Waxman, 1992; Basu and Licardie, 1993; Bradski and Grossberg, 1995), using the foundation that is summarized in the section 9.2.

The present model simulates 2D images composed of non-overlapping natural objects. Future model extensions need to incorporate mechanisms for processing 2D images and 3D scenes with overlapping objects to show how partially occluded objects can be separated from their occluders and completed in a way that facilitates their recognition. FACADE theory proposes neural mechanisms whereby 3D vision and figure-ground separation occur, and these mechanisms have been embodied in laminar cortical circuits within the 3D LAMINART model (Grossberg, 1994; Grossberg and McLoughlin, 1997; Grossberg and Raizada, 2000; Kelly and Grossberg, 2000; Grossberg and Howe, 2003; Cao and Grossberg, 2005, 2012; Grossberg and Yazdanbakhsh, 2005; Fang and Grossberg, 2009). These mechanisms can extend the current model to carry out searches of scenes with partially occluded objects.

In order to achieve contextually-cued search, ARTSCAN Search can be combined with the ARTSCENE Search model (Huang and Grossberg, 2010) to enable sequences of spatial and object information to be stored in parallel working memories, categorized, and used to determine contextually-sensitive search decisions, by using interactions between ITa, perirhinal cortex (PRC), and ventral prefrontal cortex (VPFC) in the What stream, and PPC, parahippocampal cortex (PHC), and dorsolateral prefrontal cortex (DLPFC) in the Where stream.

Spatial attention may be distributed between several objects at a time, and a scene does not go dark around a focally attended object (Eriksen and Yeh, 1985; Downing, 1988; Pylyshyn and Storm, 1988; Yantis, 1992; McMains and Somers, 2005). Foley et al. (2012) extended the ARTSCAN model to the *distributed* ARTSCAN (dARTSCAN) model to analyze how parietal and prefrontal representations of spatial attention can together enable multi-focal attention to occur, including focal attention on an object to be learned and distributed attention to the rest of the scene, using a combination of sustained surface-driven spatial attention and transient motion-driven spatial attention, thereby enabling both attentional priming of positions where an object recently disappeared or was occluded and rapid transient interruptions of attention. This extension enables many more data to be simulated, including data about two-object cueing, useful-field-of-view, and crowding.

In summary, ARTSCAN Search can be self-consistently extended by using related models in the ARTSCAN modeling framework to enable: figure-ground separation and completion of overlapping objects in both 2D pictures and 3D scenes, contextually-cued search, and multi-focal attention and multiple target tracking as part of its invariant object category learning, recognition, and Where’s Waldo search capabilities.

### Conflict of interest statement

The authors declare that the research was conducted in the absence of any commercial or financial relationships that could be construed as a potential conflict of interest.

## Appendix: model equations for invariant object category learning and recognition

The model is a network of point neurons with single compartment membrane voltage, *V*(*t*), that obeys:

(A1) $$\begin{array}{l} C_{m}\frac{dV(t)}{dt}=-\left[V(t)-E_{leak}\right]\gamma_{leak}(t)-\left[V(t)-E_{excit}\right]\gamma_{excit}(t)\\ \;-\left[V(t)-E_{inhib}\right]\gamma_{inhib}(t), \end{array}$$

(Grossberg, 1973, 1980a,b). Constant *C_m_* is the membrane capacitance; the constant conductance γ_*leak*_ controls membrane leakage; and the time-varying conductances γ_*excit*_(*t*) and γ_*inhib*_(*t*), respectively, represent the total excitatory and inhibitory inputs to the neurons as specified by the model architecture. The three *E* terms represent reversal potentials. At equilibrium, the equation can be written as:

(A2) $$V=\frac{E_{excit}\gamma_{excit}+E_{inhib}\gamma_{inhib}+E_{leak}\gamma_{leak}}{\gamma_{excit}+\gamma_{inhib}+\gamma_{leak}},$$

Thus, increases in the excitatory and inhibitory conductance lead to depolarization and hyperpolarization of the membrane potential, respectively. All conductances contribute to divisive normalization of the membrane potential, as shown as the denominator in Equation (A2). This divisive effect includes the special case of pure “shunting” inhibition when the reversal potential of the inhibitory channel is close to the neuron’s resting potential (Borg-Graham et al., 1998). Equation (A2) can be re-written as:

(A3) $$\frac{dX}{dt}=-A_{X}X+\left(B_{X}-X\right)\gamma_{excit}-\left(C_{X}+X\right)\gamma_{inhib},$$

by setting *C_m_* = 1, *X* = *V*, *A_X_* = γ_*leak*_, *E_leak_* = 0, *B_X_* = *E_excit_*, and *C_X_* = −*E_inhib_*. Figure 7 summarizes the model interactions and the variables at every model stage. All the variables that represent cell activities in a given brain region are listed in Table A1. The adaptive weights are marked as *W* with superscripts and subscripts and the connection descriptions are listed in Table A2. Superscript letters signify the pre-synaptic cell and the postsynaptic cell, respectively. For example, the weight from the neuron with activity *X*_*i*_ to the neuron with activity *Y_j_* is denoted by *W^XY^_ij_*. The model receives 2-D 500 × 500 pixel gray-scaled images to represents the visual field during category learning and also receives a constant volition control signal while the system performs primed search. Model parameters were chosen to illustrate how attentional shrouds may be sequentially activated when a simulated scene contains multiple objects, in particular how object surface with highest contrast activities can competitively form the winning shroud while inhibiting other possible shrouds in the spatial attention map. The simulations were carried out in MathWorks MATLAB R2009a on a Microsoft Windows XP ×64 with Intel Quad-Core/2.33 GHz/14 GB of RAM.

**Table A1:** **The variables in the mathematical equations that represent the model brain regions**.

**Symbols**	**Brain region**
*X*^*g*±^	Later geniculate nucleus (LGN)
*Y^g^*, *Z^g^*	Primate visual cortex V1 (V1)
*S*^*g*±^, *S^F^*	Primate visual cortex V2/V4 (V2/V4)
*B^g^*	Primate visual cortex V2 (V2)
*C*	Primate visual cortex V3 (V3)
*E, E, y^R^*	Superior colliculus (SC)
*I^U^*, *I^D^*	Lateral intra-parietal cortex (LIP)
*A*, *y^A^*, *A^I^*, *R_WHERE_*, *y^R^*	Posterior parietal cortex (PPC)
*V*^(*q*)^, *V*(*q*), *V^I,q^*	Posterior inferotemporal cortex (ITp)
*O, O*	Anterior inferotemporal cortex (ITa)
*F, F*	Orbitofrontal cortex (ORB)
*D, D, U, T*	Amygdala (AMYG)
*N, N, P*	Prefrontal cortex (PFC)
*G*	Basal ganglia (BG)

**Table A2:** **The adaptive weight variables in the model mathematical equations**.

**Symbols**	**Description**
*W^BV^*	Object boundary to view-specific category
*W^VB^*	View-specific category to object boundary
*W^VO, q^*	View category integrator to view-invarient object category
*W^OV, q^*	View-invarient object category to view category integrator
*W^OF^*	View-invarient object category to object-value category
*W^FO^*	Object-value category to view-invarient object category
*W^OD^*	View-invarient object category to value category
*W^DF^*	Value category to object-value category
*W^FN^*	Object-value category to name category
*W^NF^*	Name category to object-value category
*W^VE^*	View-specific category to eye movement map

In the superscript notation for the weights W, the first letter represents the presynaptic population and the second letter the postsynaptic population.

### A. Retina and primary visual cortex processes

#### A.1. Retina and LGN cells

Due to the focus on the high-level interactions of the cortical What and Where streams in the model, we simplify the front-end image processing of the model. The retinal and LGN polarity-sensitive cells include ON and OFF types. The ON-cells (on-center off-surround) have small excitatory center and broader inhibitory surround receptive fields, whereas the receptive fields of the OFF-cells (off-center on-surround) have the converse relation to the ON-cells. When these fields are approximately balanced, the network discounts the illuminant and contrast-normalizes cell responses (Grossberg and Todorović, 1988). Multiple scales (small, medium, large) input to the boundary and surface representations that are used to drive spatial attention, category learning, and search. The equilibrium activities *X*^*g*+^_*ij*_ and *X*^*g*−^_*ij*_ of ON and OFF output cells, respectively, at position (*i*, *j*) with scale *g* = 1, 2, 3 (small, medium, large), are defined by:

(A4) $$X_{ij}^{g+}={\left[x_{ij}^{g}\right]}^{+}\left(1+{\hat{G}}_{ij}\right),$$

(A5) $$X_{ij}^{g-}={\left[-x_{ij}^{g}\right]}^{+}\left(1+{\hat{G}}_{ij}\right),$$

where

(A6) $$x_{ij}^{g}=\frac{\sum_{pq}I_{pq}\left(D_{pqij}^{cg}-D_{pqij}^{sg}\right)}{1+\sum_{pq}I_{pq}\left(D_{pqij}^{cg}+D_{pqij}^{sg}\right)},$$

*I_pq_* is the image input at position (*p*, *q*), *D^cg^_pqij_*, and *D^sg^_pqij_* are, respectively, the Gaussian on-center and off-surround receptive fields:

(A7) $$D_{pqij}^{vg}=N^{vg}\exp\left(-\frac{{\left(i-p\right)}^{2}+{\left(j-q\right)}^{2}}{2\sigma_{vg}^{2}}\right),\;v=c,s,$$

where constant *N^vg^* in Equation (A7) is chosen so that *N^vg^*∑_pq_ *D^vg^_pqij_* = 1. The LGN receptive fields with small, medium, and large scales are cut off at 5 × 5, 17 × 17, and 41 × 41 pixels, respectively. The on-center and off-surround scale variances are determined by (σ_*c*1_, σ_*c*2_, σ_*c*3_) = (0.3, 0.75, 2) and (σ_*s*1_, σ_*s*2_, σ_*s*3_) = (1, 3, 7), respectively. []^+^ denotes half-rectified activities with [*a*]^+^ = max (*a*, 0). A foveal advantage $\hat{G}$_*ij*_ in Equations (A4, A5) is defined by a large 2D Gaussian kernel placed in the center of the visual field which amplifies the activity of the corresponding LGN cells, so that objects near the fovea have gain-amplified representations that facilitate their recognition:

(A8) $${\hat{G}}_{ij}=\exp\left(-\frac{i^{2}+j^{2}}{2\text{ ​​​}\cdot \;6^{2}}\right).$$

#### A.2. V1 polarity-sensitive oriented simple cells

The oriented simple cells in primary visual cortical area V1 receive bottom-up activated LGN ON and OFF cell activities which are sampled as oriented differences at each image location. The simple cell, *Y^g^_ijk_*, of orientation *k* and scale *g* obeys:

(A9) $$Y_{ijk}^{g}=\sum_{\left(p,q\right)}y_{pqijk}^{g}X_{pq}^{g+}-\sum_{\left(p,q\right)}y_{pqijk}^{g}X_{pq}^{g-},$$

where *X*^*g*+^_*ij*_ and *X*^*g*−^_*ij*_ are the on-center and off-center LGN outputs at position (*i*, *j*), respectively, and the simple cell filter, *y^g^_pqijk_*, is composed of oriented odd-symmetric Gabor filter kernels (Figure 13A2) that are constructed from the combination of a sinusoid function with a Gaussian function:

(A10) $$y_{pqijk}^{g}=\frac{1}{2\pi \sigma_{hg}\sigma_{vg}}\exp\left(-\frac{1}{2}\left(\frac{x^{\prime }2_{pqijk}}{\sigma_{hg}^{2}}+\frac{y^{\prime }2_{pqijk}}{\sigma_{vg}^{2}}\right)\right)\cos\left(2\pi \frac{{x^{\prime }}_{pqijk}}{\lambda_{g}}\right),$$

where λ is the wavelength of the sinusoid factor with (λ_1_, λ_2_, λ_3_) = (3, 5, 7), *g* is the scale factor, σ is the variance of the Gaussian envelope with short-axis variance (σ_*v*1_, σ_*v*2_, σ_*v*3_) = (1, 1.5, 2) and long-axis variance (σ_*h*1_, σ_*h*2_, σ_*h*3_) = (3, 4.5, 6), and. *x*′_*pqijk*_ and *y*′_*pqijk*_ are given by:

(A11) $${x^{\prime }}_{pqijk}=(p-i)\cos\left(\frac{\pi k}{4}\right)+(q-j)\sin\left(\frac{\pi k}{4}\right),$$

and

(A12) $${y^{\prime }}_{pqijk}=-(p-i)\sin\left(\frac{\pi k}{4}\right)+(q-j)\cos\left(\frac{\pi k}{4}\right).$$

The simple cell receptive fields with small, medium, and large scales are cut-off at 19 × 5, 29 × 7, and 39 × 9 pixels, respectively. The outputs from model simple cells include both ON-cells and OFF-cells which respond to opposite contrast polarities before being half-wave rectified:

(A13) $$Y_{ijk}^{g+}={\left[Y_{ijk}^{g}\right]}^{+},$$

and

(A14) $$Y_{ijk}^{g-}={\left[-Y_{ijk}^{g}\right]}^{+}.$$

#### A.3. V1 polarity-insensitive complex cells

The activities of polarity-insensitive complex cells, *z^g^_ij_*, are determined by summing the half-wave rectified outputs of polarity-sensitive cells at the same position (*i*, *j*):

(A15) $$z_{ij}^{g}=\sum_{k}\left(Y_{ijk}^{g+}+Y_{ijk}^{g-}\right),$$

where *Y*^*g*+^_*ijk*_ and *Y*^*g*−^_*ijk*_ are the outputs of on-center and off-center polarity-sensitive cell activities, respectively. The output signals of the complex cells, *Z^g^_ij_*, are normalized by divisive normalization (Grossberg, 1973, 1980b) at each position:

(A16) $$Z_{ij}^{g}={\left[\frac{{\left(z_{pq}^{g}\right)}^{2}}{0.1^{2}+\sum_{pq}L_{pqij}{\left(z_{pq}^{g}\right)}^{2}}\right]}^{+},$$

where *L_pqij_* is a Gaussian kernel:

(A17) $$L_{pqij}=\frac{1}{2\pi }\exp\left(-\frac{{\left(i-p\right)}^{2}+{\left(j-q\right)}^{2}}{2}\right).$$

Divisive normalization helps to suppress stimuli that are presented outside of the receptive fields of neurons and sharpen the *Z^g^_ij_* boundaries around an object (Grossberg and Mingolla, 1985; Heeger, 1992; Schwartz and Simoncelli, 2001). Since the ARTSCAN Search model focuses on higher-level interactions between the What and Where cortical streams that process non-overlapping natural images with complete boundaries, several image preprocessing stages are simplified or omitted, such as interactions between cortical layers in V1 and V2 that contribute to boundary completion and figure-ground separation in response to 2D images and 3D scenes. Such interactions, which are modeled in articles about FACADE theory and the 3D LAMINART model such as Cao and Grossberg (2005, 2012), Grossberg (1999), Grossberg and Kelly (1999), Grossberg and Swaminathan (2004), and Grossberg and Yazdanbakhsh (2005), can be self-consistently added to the current model.

#### A.4. V2 boundaries and surface-to-boundary attentional priming

The object boundary activities *B^g^_ij_* are computed using small, medium, and large receptive fields, or scales, *g* that receive multiple-scale bottom-up inputs from the complex cells *Z^g^_ij_*. Each scale also receives modulatory surface-to-boundary feedback signals ∑*_pq_ C_pq_F_pqij_* from surface contours *C_pq_* that surround successfully filled-in surfaces; namely, surfaces that fill-in within closed, connected boundaries (Grossberg, 1994; Kelly and Grossberg, 2000; Grossberg and Yazdanbakhsh, 2005).

Surface contours of large-scale boundaries are also strengthened by top-down spatial attentional signals ∑_*q*_ ∑_*k*_*m*(*V*^(*q*)^_*k*_)*W^VB,q^_qkij_* from the currently active view- and position-specific category. This enhancement helps to drive indirect searches for a Waldo object that codes this category. In all, the object boundary activities *B^g^_ij_* at position (*i*, *j*) and scale *g* have the equilibrium value:

1. for small and medium boundary scales,
  (A18) $$B_{ij}^{g}={\left[\text{​}\frac{Z_{ij}^{g}\left(1+10^{4}\sum_{pq}C_{pq}F_{pqij}\right)-0.4\sum_{pq}C_{pq}}{0.1+Z_{ij}^{g}\left(1+10^{4}\sum_{pq}C_{pq}F_{pqij}\right)+0.4\sum_{pq}C_{pq}}\text{​}\right]}^{+}\text{​​​​},g=1,2;$$
2. for the large scale,
  (A19) $$\text{​​​​​​​​​​​​}B_{ij}^{3}={\left[\frac{\begin{array}{c} Z_{ij}^{3}\left(1+10^{4}\sum_{pq}C_{pq}F_{pqij}+\sum_{q}\sum_{k}m\left({\bar{V}}_{k}^{\left(q\right)}\right)W_{qkij}^{VB,q}\right)\\ -0.4\sum_{pq}C_{pq} \end{array}}{\begin{array}{c} 0.1+Z_{ij}^{3}\left(1+10^{4}\sum_{pq}C_{pq}F_{pqij}+\sum_{q}\sum_{k}m\left({\bar{V}}_{k}^{\left(q\right)}\right)W_{qkij}^{VB,q}\right)\\ +0.4\sum_{pq}C_{pq} \end{array}}\right]}^{+}\text{​​​​​},\;\;\;\;\;\;$$
  where *Z^g^_ij_* is the bottom-up complex cell input with three scales *g* = 1, 2, 3, defined in Equation (A16); *C_pq_* is the surface contour cell activity at position (*p*, *q*), defined in Equation (A27); and *F_pqij_* is the Gaussian kernel from position (*p*, *q*) on the surface contour to position (*i*, *j*) on the object boundary:
  (A20) $$F_{pqij}=\frac{1}{2\pi \;\cdot \;5^{2}}\exp\left(-\frac{{\left(i-p\right)}^{2}+{\left(j-q\right)}^{2}}{2\;\cdot \;5^{2}}\right).$$

In Equation (A19), the signal function *m* is defined by the sigmoid function:

(A21) $$m(a)=\frac{{\left[a\right]}^{+}}{0.001+{\left[a\right]}^{+}},$$

*m*(*V*^(*q*)^_*k*_) is the output signal from the *kth* view-specific category neuron in position *q*, defined in Equation (A55), and *W^VB,q^_qkij_* is the adaptive weight from the *kth* view-specific category neuron to the object boundary in position *q*. Boundary position *q* is defined by a small region of the input scene into which an exemplar of an object can occur. In the simulations, a 500 × 500 pixel input scene is divided into 25 regions with 100 × 100 pixels. The large-scale boundary Equation (A19) in each region can drive view-specific category learning of the object [see Equations (A55–A60)] and, as shown in Equation (A19), can receive learned top-down modulatory inputs from the corresponding learned view-specific category neurons. Such large-scale boundary information alone, without additional surface information about lightness or color, is sufficient to carry out accurate Where’s Waldo searches of the natural objects in the currently simulated data base.

#### A.5. V2 surface filling-in

Inputs from ON and OFF LGN cells activate a non-linear diffusion process within surface Filling-In DOmain, or FIDO, cells. The spread of LGN-activated surface activities is gated, or inhibited, by boundary signals. The LGN inputs are also modulated by top-down attentional inputs from whatever surface-shroud resonances are active. These attentional inputs increase the contrasts of the filled-in surface activities, and thus the surface contours of the attended surface, leading to preferential choice of eye movements on that surface. The attentional inputs are mediated by gain fields that convert the head-centered shroud back to retinotopic coordinates. The surface neurons also receive inhibitory inputs from reset neurons in the Where stream that facilitate instatement of the next surface to be attended after a spatial attentional shift. The ON and OFF cell surface activities *S*^*g*+^_*ij*_ and *S*^*g*−^_*ij*_, respectively, at scale *g* and position *(i, j)* are:

(A22) $$\begin{array}{l} \frac{dS_{ij}^{g+}}{dt}=-80S_{ij}^{g+}\text{​}+\text{​}\sum_{\left(p,\;q\;\in \;\Delta_{ij}\right)}P_{pqij}^{g}\left(S_{pq}^{g+}\text{​}-\text{​ }S_{ij}^{g+}\right)+100X_{ij}^{g+}\left(1\text{ ​}+\text{ ​}S_{ij}^{F}\right)\\ \;-S_{ij}^{g+}R_{WHERE}y^{R}, \end{array}$$

and

(A23) $$\begin{array}{l} \frac{dS_{ij}^{g-}}{dt}=-80S_{ij}^{g-}\text{​}+\text{​}\sum_{\left(p,q\;\in \;\Delta_{ij}\right)}P_{pqij}^{g}\left(S_{pq}^{g-}\text{​}-\text{​ }S_{ij}^{g-}\right)\text{​ }+\text{ ​}100X_{ij}^{g-}\left(1\text{ ​}+\text{ ​}S_{ij}^{F}\right)\\ \;-S_{ij}^{g-}R_{WHERE}y^{R}, \end{array}$$

where *X*^*g*+^_*ij*_ and *X*^*g*−^_*ij*_ are the bottom-up input signals from ON and OFF LGN neurons, *S^F^_ij_* are top-down attentional inputs from the gain field neurons defined in Equation (A36), *R_WHERE_* is the category reset signal defined in Equation (A50), and *y^R^* is the reset habituative transmitter defined in Equation (A52). The boundary-gated diffusion coefficient, *P_pqij_*, that regulates the magnitude of activity spread between position (*i*, *j*) and position (*p*, *q*) obeys:

(A24) $$P_{pqij}^{g}=\frac{10^{4}}{1+40\left(B_{pq}^{g}+B_{ij}^{g}\right)},$$

where

(A25) $$\Delta_{ij}=\left\{\left(i,j-1),(i-1,j),(i+1,j),(i,j+1\right)\right\},$$

are the nearest-neighbor neurons with which the diffusion occurs around cell (*i*, *j*).

After ON and OFF filling-in processes occur, the outputs from different scales are pooled to form a multiple-scale output signal (Hong and Grossberg, 2004):

(A26) $$S_{ij}=0.05\left(S_{ij}^{1+}+S_{ij}^{1-}\right)+0.1\left(S_{ij}^{2+}+S_{ij}^{2-}\right)+0.85\left(S_{ij}^{3+}+S_{ij}^{3-}\right).$$

This weighted distribution of scales, with the largest weight given to the large scale to produce a more homogeneous surface representation, is used in the competition for spatial attention to choose a winning shroud.

#### A.6. Surface contours

The filled-in ON and OFF surface activities across multiple scales of the attended object surface are averaged before being contrast-enhanced by on-center and off-center networks, half-wave rectified, and added to generate surface contour output signals *C_ij_* at position (*i*, *j*):

(A27) $$\begin{array}{l} C_{ij}={\left[\frac{\sum_{pq}(J_{pq}^{+}-J_{pq}^{-})(K_{pqij}^{+}-K_{pqij}^{-})}{40+\sum_{pq}(J_{pq}^{+}-J_{pq}^{-})(K_{pqij}^{+}+K_{pqij}^{-})}\right]}^{+}\\ \;+{\left[\frac{\sum_{pq}(J_{pq}^{+}-J_{pq}^{-})(K_{pqij}^{-}-K_{pqij}^{+})}{40+\sum_{pq}(J_{pq}^{+}-J_{pq}^{-})(K_{pqij}^{+}+K_{pqij}^{-})}\right]}^{+}. \end{array}$$

Surface contours strengthen the boundaries that formed them and inhibit spurious boundaries, as in Equations (A18, A19). When a surface-shroud resonance is active, it enhances the activation of the attended surface via gain field neurons, as in Equations (A22, A23). The enhanced surface activation, in turn, strengthens the surface contours of the surface via the signals *J*^+^_*pq*_ and *J*^−^_*pq*_ that are defined below.

In addition to selecting and strengthening the boundaries that formed them, surface contours are also processed in a parallel pathway that controls the target positions of eye movements that scan the attended object, as in Equation (A43). The role of surface contours in target selection is possible because surface contours occur at positions where surface brightnesses and colors change quickly across space, and thus mark positions where salient features exist on the surface. When surface contours signals are strengthened by spatial attention, they can compete more effectively in the eye movement map Equation (A43) to determine the positions to which the eyes will move, therefore restricting scanning eye movements to the attended surface while its shroud is active.

Surface contours are determined by weighted ON and OFF averages, *J*^+^_*pq*_ and *J*^−^_*pq*_, respectively, of filled-in surface activities across scales at each position (*p*, *q*). These averages give greater weight to the small scale because it computes better localized, signals around the salient features of the filled-in surface:

(A28) $$J_{pq}^{+}=0.8S_{ij}^{1+}+0.1S_{ij}^{2+}+0.1S_{ij}^{3+}.$$

and

(A29) $$J_{pq}^{-}=0.8S_{ij}^{1-}+0.1S_{ij}^{2-}+0.1S_{ij}^{3-},$$

where *K*^+^_*pqij*_ and *K*^−^_*pqij*_ are on-center and off-center Gaussian kernels, respectively:

(A30) $$K_{pqij}^{+}=\frac{1}{2\pi }\exp\left(-\frac{{\left(i-p\right)}^{2}+{\left(j-q\right)}^{2}}{2}\right),$$

(A31) $$K_{pqij}^{-}=\frac{1}{2\pi \;\cdot \;3^{2}}\exp\left(-\frac{{\left(i-p\right)}^{2}+{\left(j-q\right)}^{2}}{2\;\cdot \;3^{2}}\right).$$

### B. Where stream

#### B.1. Gain field

Model processes prior to the spatial attentional map are all in retinotopic coordinates, so that object positions change with every eye movement. In contrast, the spatial attention map is in head-centered coordinates that are invariant to changes in eye position. Gain fields mediate this transformation (Andersen and Mountcastle, 1983; Andersen et al., 1985; Grossberg and Kuperstein, 1986, 1989; Pouget and Sejnowski, 1997; Pouget and Snyder, 2000; Deneve and Pouget, 2003). ARTSCAN adapted the gain field model of Pouget and Snyder (2000), but this model becomes computationally unwieldy when processing natural images. However, the implementation of gain filed transformation in the ARTSCAN model increases the computational loads when input image becomes large.

To overcome this problem, ARTSCAN Search modifies the gain field model of Cassanello and Ferrera (2007), which computes the visual remapping using a product of maps instead of a linear combination. In addition, ARTSCAN Search separates the gain fields into two parallel channels, a bottom-up channel and a top-down channel. The bottom-up channel receives bottom-up retinotopic surface inputs which are shifted according to the eye position to the head-centric map, whereas the top-down channel transforms the top-down head-centric map to a retinotopic map, again modulated by eye position.

When both retinal and eye position maps are two-dimensional, the gain field will be four-dimensional. In the bottom-up channel, the activity *I^U^_mnij_* of gain field cell at position (*m*, *n*, *k*, *l*) is the product of the eye position map with the sum of the object surface map and the spatial attentional map:

(A32) $$I_{mnkl}^{U}=\left(S_{m\;-\;k,n\;-\;l}+A_{mn}\right){\bar{E}}_{kl},$$

where is the object surface activity at position (*m* − *k*, *n* − *l*), whose coordinates are shifted by the eye position at position (*k*, *l*), *A_mn_* is the activity of spatial attention at position (*m*, *n*), and

(A33) $${\bar{E}}_{kl}=\{\begin{array}{l} 1\text{ if eye position at }(k,\;l),\\ 0\text{ otherwise}. \end{array}$$

The output signals *A^I^_mn_* from the gain field to the spatial attentional map are defined as the sum of all the gain field maps corresponding to different eye positions:

(A34) $$\begin{array}{l} A_{mn}^{I}=\sum_{kl}I_{mnkl}^{U}=\sum_{kl}\left(S_{m\;-\;k,n\;-\;l}+A_{mn}\right){\bar{E}}_{kl}=S_{m\;-\;k_{0},n\;-\;l_{0}}\\ \;+A_{mn}\text{ if eye position is at }\left(k_{0},\;l_{0}\right) \end{array}$$

In the top-down channel, as in the bottom-up channel, the activity *I^D^_mnij_* of the gain field cell at position (*m*, *n*, *k*, *l*) is the product of the eye position map at position (*k*, *l*) with the sum of the shifted spatial attention map and the eye position map:

(A35) $$I_{mnkl}^{D}=\left(A_{m+k,n+l}+S_{mn}\right){\bar{E}}_{kl},$$

where *A*_*m* + *k*, *n* + *l*_ is the activity of spatial attention at position; (*m* + *k*, *n* + *l*);, whose coordinates are shifted by the eye position at position (*k*, *l*), and *S_mn_* is the object surface cell activity at the position (*m*, *n*). The output signals *S^F^_mn_* from the gain field to the object surface are again defined as the sum of all gain field maps across all the eye position maps.

(A36) $$\begin{array}{l} S_{mn}^{F}=\sum_{kl}I_{mnkl}^{D}=\sum_{kl}\left(A_{m+k,n+l}+S_{mn}\right){\bar{E}}_{kl}=A_{m+k_{0},n+l_{0}}\\ \;+S_{mn}\text{ if eye position is at }\left(k_{0},\;l_{0}\right). \end{array}$$

#### B.2. Spatial attention: attentional shroud

The outputs of the bottom-up gain field input to the spatial attention layer, where spatial competition chooses the *attentional shroud*. The shroud, in turns, feeds back via the top-down gain field to object surface representations and thereby enhances the activities of the winning surface.

The spatial attention neurons receive excitatory bottom-up inputs from the corresponding gain field neurons, as well as modulatory lateral excitation from other spatial attention neurons that is gated by habituative transmitters (Grossberg, 1972b, 1980b). Each spatial attention neuron also receives shunting lateral inhibition from the sum of gain field and attentional output signals, as well as transient reset signals that are also gated by habituative transmitters. The spatial attention neuronal activity *A_ij_* at position (*i*, *j*) thus obeys:

(A37) $$\begin{array}{l} \frac{1}{10}\frac{dA_{ij}}{dt}=-0.1A_{ij}\text{​}+\text{ ​}\left(1\text{ ​}-\text{​}A_{ij}\right)\left(g(A_{ij}^{I})\left(1\text{​ }+\text{ ​}0.2\sum_{mn}f(A_{mn})C_{mnij}\right)\right)y_{ij}^{A}\\ \;-A_{ij}\left(\sum_{mn}\left(g\left(A_{mn}^{I}\right)+f\left(A_{mn}\right)\right)E_{mnij}+10R_{WHERE}y^{R}\right), \end{array}$$

where *A^I^_ij_* is the gain field input defined in Equation (A39), *y^A^_ij_* is the excitatory habituative transmitter that gates the gain field output signal *g*(*A^I^_ij_*) and the total spatial attentional input 0.2∑*_mn_f*(*A_mn_*)C_mnij_ at position (*i*, *j*); see Equation (A42). *R_WHERE_* is the category reset signal defined in Equation (A50), and *y^R^* is the reset habituative transmitter; see Equation (A52). The gain field signal function *g* is defined by the threshold-linear function:

(A38) $$g(a)={\left[a-0.05\right]}^{+},$$

the attentional signal function *f* is defined by sigmoid function:

(A39) $$f(a)=\frac{4a^{4}}{0.35^{4}+a^{4}},$$

*C_mnij_* is the Gaussian excitatory on-center kernel from position (*m*, *n*) to (*i*, *j*):

(A40) $$C_{mnij}=\frac{1}{10}\exp\left(-\frac{{\left(i-m\right)}^{2}+{\left(j-n\right)}^{2}}{2\;\cdot \;4^{2}}\right),$$

and *E_mnij_* is the Gaussian inhibitory off-surround kernel:

(A41) $$E_{mnij}=\frac{1}{2\;\cdot \;10^{5}}\exp\left(-\frac{{\left(i-m\right)}^{2}+{\left(j-n\right)}^{2}}{2\;\cdot \;200^{2}}\right).$$

In Equation (A37), the habituative transmitter *y^A^_ij_* that mediates between the gain field output and its spatial attention cell at position (*i*, *j*) obeys:

(A42) $$\frac{dy_{ij}^{A}}{dt}=K_{A}\left(2-y_{ij}^{A}-3\;\cdot \;10^{6}\left(g(A_{ij}^{I})\left(1+0.2\sum_{mn}f(A_{mn})C_{mnij}\right)\right)y_{ij}^{A}\right),$$

where *K_A_* = 10^−8^ is a slow rate that allows the persistence of the attentional shroud during eye movement explorations of the attended object surface, 2 − *y^A^_ij_* is proportional to the rate of transmitter accumulation and the attentionally-modulated gain field input 3· 10^6^ (*g* (*A^I^_ij_*) (1 + 0.2∑*_mn_f* (*A_mn_*) *C_mnij_*)) *y^A^_ij_* determines the rate of transmitter inactivation. As the habituative transmitter depletes, the activity in the shroud neurons can collapse enough to trigger the reset signals that enable another group of neurons to form a shroud around a newly chosen surface representation.

The reset signals in the Where stream are rendered transient by habituative gates; e.g., see Equation (A37). Without these gates, the otherwise tonically active reset signals could keep the spatial attention network inhibited permanently.

#### B.3. Eye movements to salient features on the attended surface

The eye movement map receives inputs from the surface contour cells and contrast-enhances them using a recurrent on-center off-surround network to choose the most active neuron activity as the next target for fixation. This decision is also influenced by input from the currently active view-specific category which provides a direct learned route from positionally-sensitive categories in the What stream to target positions in the Where stream (see section 9). All the excitatory inputs are gated by habituative transmitters, which prevent perseveration on the previous target choice. The eye movement cell activity *E_ij_* at position (*i*, *j*) obeys:

(A43) $$\begin{array}{l} \frac{dE_{ij}}{dt}=-E_{ij}+(1-E_{ij})\\ \;\left({\left[C_{ij}\right]}^{+}+625\sum_{mn}E_{mn}^{2}J_{mnij}+\sum_{q}\sum_{k}m\left({\bar{V}}_{k}^{\left(q\right)}\right)W_{qkij}^{VE}\right)y_{ij}^{E}\\ \;-0.01E_{ij}\sum_{ij}\left({\left[C_{ij}\right]}^{+}+\sum_{mn}E_{mn}^{2}K_{mnij}\right), \end{array}$$

and the most active eye movement neuronal activity *E_ij_* is selected corresponding to the next target position (*i*, *j*):

(A44) $$E_{IJ}=\{\begin{array}{l} E_{ij},\text{ if }E_{ij}=\max_{pq}(E_{pq}>0.58),\\ 0,\text{ otherwise}. \end{array},$$

In Equation (A43), *C_ij_* is the surface contour neuron activity at position (*i*, *j*), *m*(*V*^(*q*)^_*k*_) is the output signal from view-specific category neuron defined in Equation (A55), where the sigmoid signal function *m*(*a*) is defined in Equation (A21), *J_mnij_* is the Gaussian excitatory on-center kernel:

(A45) $$J_{mnij}=\frac{1}{2\pi }\exp\left(-\frac{{\left(i-m\right)}^{2}+{\left(j-n\right)}^{2}}{2}\right),$$

*K_mnij_* is the Gaussian inhibitory off-surround kernel:

(A46) $$K_{mnij}=\frac{1}{2\pi \;\cdot \;5^{2}}\exp\left(-\frac{{\left(i-m\right)}^{2}+{\left(j-n\right)}^{2}}{2\;\cdot \;5^{2}}\right),$$

and *y^E^_ij_* is the habituative transmitter that gates the input to eye movement neuron at position(*i*, *j*):

(A47) $$\frac{dy_{ij}^{E}}{dt}=K_{E}\left(2\text{​ }-\text{​}y_{ij}^{E}\text{​}-\text{​}3\;\cdot \;10^{6}y_{ij}^{E}\left({\left[C_{ij}\right]}^{+}\text{​}+\text{​}625\sum_{mn}E_{mnij}^{2}J_{mnij}\right)\right)\text{​},\;\;\;$$

where *K_E_* = 10^−7^. Because *K_E_* in Equation (A47) is larger than *K_A_* in Equation (A42), an active shroud can be explored by several eye movements before its attentional shroud collapses. The adaptive weight *W^VE^_qkij_* from the selected *k^th^* view-specific category neuron at position *q* to the eye movement map at position (*i*, *j*) obeys:

(A48) $$\frac{1}{500}\frac{dW_{qkij}^{VE}}{dt}=m\left({\bar{V}}_{k}^{\left(q\right)}\right)h(E_{ij})\left(E_{ij}-W_{qkij}^{VE}\right),$$

where

(A49) $$h(E_{ij})=\{\begin{array}{l} 1,\text{ if }E_{ij}=\max_{pq}(E_{pq}>0.58),\\ 0,\text{ otherwise}. \end{array}$$

In Equation (A49), the function *h*(*E_ij_*) is the sign function that indicates the chosen saccadic eye movement. The weight in Equation (A48) obeys a steepest descent learning law that is called *outstar learning* (Grossberg, 1980b, WHERE Stream). Due to outstar learning, when the category *V*^(*q*)^_*k*_ is active and the eye position activity *E_ij_* is chosen, then the weight *W^VE^_qkij_* approaches *E_ij_*.

#### B.4. Object category reset by transient parietal bursts

Category reset neurons are tonically active. Their tonic activity is inhibited by inputs from all the active cells across the spatial attention map. When an attentional shroud collapses, the reset neurons are disinhibited, and generate a transient activity burst that inhibits, and thus resets, the spatial attention and object surface maps in the Where stream, as well as the view category integrator neurons in the What stream. The activity *R_WHERE_* of the reset cells obeys:

(A50) $$R_{WHERE}=100{\left[\frac{100}{100+\sum_{ij}k(A_{ij})}-\varepsilon \right]}^{+},$$

where *A_ij_* is the activity of the spatial attention neuron at position (*i*, *j*), function *k* is defined by a steep sigmoid function:

(A51) $$k(a)=\frac{{\left[a^{35}\right]}^{+}}{0.22^{35}+{\left[a^{35}\right]}^{+}},$$

with activity threshold [*w*]^+^ = max (*w*, 0) to count the number of cells in the shroud that have activity greater than 0.22, and ε = 0.07 equals the threshold total activity above which the reset signal *R_WHERE_* turns on. By Equation (A50), the total output signal ∑*_ij_ k*(*A_ij_*) from the shroud inhibits reset by making term $\frac{100}{100+\sum_{ij}k(A_{ij})}$ smaller than ε. When the total shroud output gets small enough, $\frac{100}{100+\sum_{ij}k(A_{ij})}$ exceeds ε and the reset signal fires (Figure 10B). The reset rule in Equation (A50) is more sensitive to the gradual collapse of an active shroud and better able to completely reset the system after a spatial attention shift than the reset rule used in Fazl et al. (2009).

Shortly after the transient reset is triggered, its activity-dependent neurotransmitter *y^R^* habituates:

(A52) $$\frac{dy^{R}}{dt}=6\left(2-y^{R}-2y^{R}R_{WHERE}\right),$$

thereby terminating the net reset signal 10*R_WHERE_y^R^* in Equation (A37). The transmitter gradually replenishes through time while a new object is attended, until the next reset event occurs (Figure 10C).

### C. What stream

The inputs to the category-learning neurons in area ITp of the model’s What stream are the object boundary neuron outputs in Equation (A19), which are connected to view-specific category neurons through adaptive weights. While the surface-shroud resonance of a particular object remains active, the view category integrator neurons that are activated by that object’s view-specific category neurons remain active even after the corresponding view-specific category neurons are reset in response to eye movements that activate different boundary inputs. These view category integrator neurons are associated with invariant object category neurons in the model’s area ITa, which in turn are associated with name category neurons in its prefrontal cortex. The first view-specific category to be activated in ITp by a given object boundary activates cells in ITa that will become the invariant object category through associative learning with multiple view category integrator neurons. This learning is modulated by a cognitive-emotional resonance through the invariant category, value category, and object-value category neurons.

#### C.1. View-specific categories

*Fuzzy* ART learns the view-specific categories (Carpenter et al., 1991, 1992). As noted in section A4, object boundary representations are presented in 25 distinct positionally-sensitive regions that enable the system to learn positionally-invariant object categories. In particular, a scene of 500 × 500 pixels is divided into 25 regions of 100 × 100 pixels. Only the large-scale boundary representations *B*^3^_*ij*_ in Equation (A19) are used as the inputs for category learning. The superscript “3” is omitted for simplicity. Each 100 × 100 subset of the object boundary representation is then denoted as *B*^(*q*)^ *B_ij_*, where *q* = 1, 2, 3, …, 25 and *i*, *j* = 12, 3, …, 100 denotes the indices of that part of the boundary vector that is restricted to the *q^th^* region. Each input boundary is transformed into an ON and OFF cell normalized input vector $\vec{B}$^(*q*)^ by *complement coding* before being presented to the Fuzzy ART algorithm:

(A53) $${\vec{B}}^{\left(q\right)}=\left\{u\left(B^{\left(q\right)}B_{ij}\right)\left(B^{\left(q\right)}B_{ij}\right),\;u\left(B^{\left(q\right)}B_{ij}\right)\left(1-B^{\left(q\right)}B_{ij}\right)\right\},$$

where *u*(*B*^(*q*)^ *B_ij_*) is given by:

(A54) $$u\left(B^{\left(q\right)}B_{ij}\right)=\{\begin{array}{l} 1,\text{ if }B^{\left(q\right)}B_{ij}>0,\\ 0,\text{ otherwise}. \end{array},$$

This transformation complement-codes the boundary within its region while eliminating spurious OFF cell “1” values in all other regions. As a result, 25 Fuzzy ART algorithms can independently learn to categorize the complement-coded boundary vectors that activate their respective regions.

As noted in section A6, boundaries are enhanced via surface contour signals when the attended object surface receives top-down excitatory feedback via a surface-shroud resonance. This property is important, as shown below, in enabling learning to occur of the attended object’s view-specific categories, which in turn supports learning of its invariant category.

In addition to the bottom-up input from an object’s boundary representation, a view-specific category also receives top-down modulatory input from the corresponding view category integrator neuron. In all, activity *V*^(*q*)^_*j*_ of the *j^th^* view-specific category neuron in position *q* in response to the boundary input $\vec{B}$^(*q*)^ obeys:

(A55) $$V_{j}^{\left(q\right)}=\left(1+0.1V_{j}^{I,q}\right)\frac{\left|{\vec{B}}^{\left(q\right)}\wedge W_{j}^{BV,q}\right|}{10^{-5}+\left|W_{j}^{BV,q}\right|},$$

where *W^BV,q^_j_* is the learned weight vector between the input vector $\vec{B}$^(*q*)^ and the *j^th^* view-specific category neuron, defined in Equation (A58), and *V^I,q^_j_* is the activity of the view category integrator cell [see Equation (A61)] which is connected one-to-one to the corresponding view-specific category neuron. The fuzzy AND operator, ∧, between two vectors is defined as(*x* ∧ *y*)_*i*_ = min (*x_i_*, *y_i_*), and the *L*_1_ norm operator, | • |, is defined as |*p*| = ∑_*i*_ |*p_i_*|. The term $\vec{B}$^(*q*)^ ∧ *W^BV,q^_j_* in the numerator can be interpreted as the expected number of learned sites *W^BV,q^_j_* that are activated by the input vector $\vec{B}$^(*q*)^. The more learned sites that get activated, the more similar are the weight and the input vector, and thus the more active the view category neuron becomes.

The most highly activated view-specific category neuron wins the competition among all active category neurons at its position; that is, the *J^th^* category neuron in position *Q* is chosen if:

(A56) $$V_{J}^{\left(Q\right)}=\underset{j}{\max}\left\{V_{j}^{\left(q\right)}:V_{j}^{\left(q\right)}>0\right\}.$$

As noted above, an attended object’s boundary representation is amplified when its surface is part of a surface-shroud resonance. This boundary enhancement influences the choice of view-specific categories via Equations (A55, A56).

The chosen view-specific category neuron is said to be in a *resonant* state if the selected neuron meets the matching criterion:

(A57) $$\frac{\left|{\vec{B}}^{\left(Q\right)}\wedge W_{J}^{BV,Q}\right|}{\left|{\vec{B}}^{\left(Q\right)}\right|}\geq \rho ,$$

where ρis the *vigilance* parameter that determines the sensitivity of network to the match of the bottom-up input vector $\vec{B}$^(*q*)^ and the learned top-down expectation with weight *W^BV,q^_j_*. Inequality Equation (A57) says that the amount of matched feature-expectation pattern, |$\vec{B}$^(*q*)^ ∧ *W^BV,Q^_J_*|, exceeds the product of the total input excitation |$\vec{B}$^(*Q*)^| and the vigilance ρ. Thus, vigilance is the *gain* of the excitatory input pattern $\vec{B}$^(*q*)^. The vigilance in the simulations is ρ = 0.85. Resonance triggers category learning in the weights *W^BV,q^_j_* between the boundary input pattern $\vec{B}$^(*q*)^ and the winning view-specific category neuron *J* in position *Q*:

(A58) $$W_{J}^{BV(new),Q}=\beta \left(W_{J}^{BV(old),Q}\wedge {\vec{B}}^{\left(Q\right)}\right)+(1-\beta )W_{J}^{BV(old),Q},$$

where the learning rate β is set to 1 for fast learning.

*Mismatch reset* occurs if inequality Equation (A57) is not satisfied. As a result, a previously active view-category neuron *J* is reset to inactive and the next most active view-specific category neuron tries to satisfy the vigilance criterion. The search process continues until the chosen winner satisfies inequality Equation (A57).

In addition, to bottom-up category learning, resonance also triggers top-down learning by the weight from the winning view-specific category neuron to the object boundary. The top-down weight *W^VB,Q^_QJmn_* in position *Q* is defined as a two-dimensional map extracted from the ON cell part of the latest updated weight *W^BV,q^_j_* which is given by:

(A59) $$W_{QJmn}^{VB,Q}=W_{J}^{BV(ON),Q}\text{ for }(m,\;n)=1,\;2,\;3,...,\;100.$$

The output signal *V*^(*q*)^_*j*_ from the *j^th^* view-specific category neuron in position *q* to the view category integrator neuron is defined by a normalized quenching competition (see section 7.3.7)as:

(A60) $${\bar{V}}_{j}^{\left(q\right)}=\{\begin{array}{ll} V_{j}^{\left(q\right)}, & \begin{array}{c} \text{if }V_{j}^{\left(q\right)}=\max_{k}(V_{j}^{\left(q\right)}),\text{ and }V_{j}^{\left(q\right)}>\\ V_{k}^{\left(q\right)}+0.03,\;k\neq j, \end{array}\\ V_{j}^{\left(q\right)}\left(\frac{V_{j}^{\left(q\right)}}{\sum_{k\in \Omega }V_{k}^{\left(q\right)}}\right), & \begin{array}{l} \text{if }\left|V_{j}^{\left(q\right)}-V_{k}^{\left(q\right)}\right|\leq 0.03,\;k\in \Omega ,\text{ and}\\ {\text{min}}_{j\;\in \;\Omega }(V_{j}^{\left(q\right)})>\max_{k\;\notin \;\Omega }(V_{k}^{\left(q\right)}+0.03), \end{array}\\ 0, & \text{if }j\in \Omega . \end{array}$$

#### C.2. View category integrators

View category integrator activities preserve the activities of view-specific category neurons during category learning. A view category integrator neuron receives bottom-up input from the corresponding view-specific category neuron in addition to top-down modulatory input from invariant object category neurons. The activity of the *j^th^* view category integrator neuron *V^I,q^_j_* in position *q* obeys:

(A61) $$\begin{array}{l} \frac{dV_{j}^{I,q}}{dt}=-0.01V_{j}^{I,q}+\tau \left(1+\sum_{i}m({\bar{O}}_{i})W_{ij}^{OV,q}\right)\\ \;\left({\left[{\bar{V}}_{j}^{\left(q\right)}\right]}^{+}+G\right)-R_{WHERE}, \end{array}$$

where *m*(*O_j_*) is the *j^th^* modulatory invariant object category output signal defined in Equation (A64), *W^OV,q^_ij_* is the top-down learned weight from the *i^th^* invariant object category neuron to the *j^th^* view category integrator neuron in position *q* defined in Equation (A75),*V*^(*q*)^_*j*_ is the *j^th^* bottom-up view-specific category neuron output signal in Equation (A60), and *R_WHERE_* is the reset signal triggered by the collapse of a spatial attentional shroud defined in Equation (A50). In Cao et al. (2011), parameter τ calibrates the duration that an object stays on at a particular retinal position to be consistent with experimental data (Li and DiCarlo, 2008) showing that the time foveating an object is approximately twice long as it stays in an extra-foveal position. The current simulations work if τ in Equation (A61) has twice the value at the fovea than it does in extra-foveal positions, or the same value in both. The simulations that are reported here use the value τ = 0.6 at all retinal positions.

Variable *G* in Equation (A61) is a basal ganglia volitional signal that is turned on only when a top-down primed search is executed. A volitional signal from the basal ganglia can change the excitatory/inhibitory balance in the modulatory on-center of a top-down expectation (Grossberg, 2000). In ARTSCAN Search, during primed search, the volition control signals project to view category integrators [Equation (A61)], invariant categories [Equation (A63)], and object-value categories [Equation (A70)] to enable a top-down prime to reach its associated view-specific categories:

(A62) $$G=\{\begin{array}{l} 0.1,\text{ during top}-\text{down primed search},\\ 0,\text{ during category learning}. \end{array}$$

#### C.3. Invariant object categories

Each invariant object category neuron is associated with multiple view-specific category neurons that represent different views and positions of the same object. The current simulations consider only positional variations, but the same mechanisms work for view changes. The invariant object category layer has the winner-take-all properties of the normalized quenching competition which selects the most active neuron in response to bottom-up input from view category integrator neurons, and a modulatory top-down feedback signal from object-value category neurons. Invariant object category neurons are connected one-to-one to object-value category neurons [Equation (A70)]. In addition, when either a top-down cognitive or a motivational primed search is processed, invariant object category neurons receive an excitatory volitional control signal that enables the primed category to fire. The activity of the *j^th^* invariant object category neuron *O_j_* thus obeys:

(A63) $$\begin{array}{l} \frac{1}{20}\frac{dO_{j}}{dt}=-O_{j}+\left(1+2{\bar{F}}_{j}W_{j}^{FO}\right)\\ \;\left(0.5\sum_{q}\sum_{i}m\left({\left[V_{i}^{I,q}\right]}^{+}\right)W_{ij}^{VO,q}+G\right)-R_{WHERE}, \end{array}$$

where *F_j_* is the modulatory top-down output signal from the *j^th^* object-value category neuron defined in Equations (A70, A71), *W^FO^_j_* is the weight defined in Equation (A77) from the *j^th^* object-value category neuron to the corresponding invariant category neuron, *m*([*V^I,q^_i_*]^+^) is the output signal from the *i^th^* view category integrator through signal function *m*(*a*) defined in Equation (A21), *W^VO,q^_ij_* is the weight defined in Equation (A65) between the *i^th^* view category integrator and the *j^th^* invariant object category, *W^FO^_j_* is the weight defined in Equation (A77) from the *j^th^* object-value category neuron to the corresponding invariant category neuron, *G* is the volitional signal from the basal ganglia defined in Equation (A62), and *R_WHERE_* is a reset signal triggered when the attentional shroud collapses defined in Equation (A50).

Unlike the reset signals within the Where stream, What stream resets [see Equations (A61, A63)] are not gated by a habituative transmitter. Instead, they are shut off by inhibition from the next shroud that forms. This prevents the previously active invariant category from being erroneously associated with view-specific categories of the next object.

The invariant object categories compete with each other during bottom-up processing to determine a winner-take-all choice of the most highly activated category. On the other hand, if multiple view-specific categories are activated by multiple equally salient bottom-up inputs, then the invariant object categories that they activate can all remain active, but their total activity is normalized. After they receive top-down primes from the object-value categories, a winner can be chosen if its representation is sufficiently more active than that of the other categories. Thus, the output signal *O_j_* from the *j^th^* invariant object category neuron to its object-value category via a bottom-up route, or its view category integrator neurons via a top-down route, obeys a normalized quenching competition that is given by:

(A64) $${\bar{O}}_{j}=\{\begin{array}{ll} {\left[O_{j}\right]}^{+}, & \begin{array}{c} \text{if }{\left[O_{j}\right]}^{+}=\max_{k}({\left[O_{k}\right]}^{+}),\\ \text{and }O_{j}>O_{k}+0.3,\;k\neq j, \end{array}\\ {\left[O_{j}\right]}^{+}\left(\frac{O_{j}}{\sum_{k\in \Omega }O_{k}}\right), & \begin{array}{c} \text{if }\left|O_{j}-O_{k}\right|\leq 0.3,\;k\in \Omega ,\text{ and}\\ {\text{min}}_{j\in \;\Omega }(O_{j})>\max_{k\notin \;\Omega }(O_{k}+0.3), \end{array}\\ 0,\; & \text{if }j\in \Omega . \end{array}$$

During learning, the weight *W^VO,q^_ij_* in Equation (A65) from the *i^th^* view category integrator to the *j^th^* invariant object category obeys a competitive outstar learning law (e.g., Grossberg, 1980b; Carpenter and Grossberg, 1987; Pilly and Grossberg, 2012):

(A65) $$\begin{array}{l} \frac{dW_{ij}^{VO,q}}{dt}=\alpha \left(1-W_{ij}^{VO,q}\right){\left[V_{i}^{I,q}\right]}^{+}m({\bar{O}}_{j})\\ \;-\beta W_{ij}^{VO,q}{\left[V_{i}^{I,q}\right]}^{+}\sum_{k\neq j}m\left({\bar{O}}_{k}\right), \end{array}$$

where *V^I,q^_i_* is the *i^th^* view category integrator activity, *m*(*O_j_*) is the invariant *j^th^* object category output through the signal function *m*(*a*) in Equation (A21), α scales the learning rate with α = 0.003, and β is competition gain with β = 0.001. It was shown in Cao et al. (2011) how this learning law enables positionally-invariant category learning to occur.

#### C.4. Value categories

Each value category, or drive representation, neuron is assumed to occur in the amygdala (Aggleton, 1993; LeDoux, 1993). It is activated by one or more invariant object category neurons. In the present simplified simulations, where each invariant object category activates only one value category. The connection between a value category neuron and a view-invariant object category neuron can convert into a conditioned reinforcer by strengthening its associative link from the category to the drive representation. Value category neurons also receive external reinforcers that combine with inputs from object category neurons to provide incentive motivation to the orbitofrontal representations (object-value category), or perform as the internal drive state to initiate top-down primed search through inferotemporal-amygdala-orbitofrontal resonance to strengthen the corresponding view-invariant object category neurons. The value category neuron activity *D_j_* obeys:

(A66) $$\frac{1}{20}\frac{dD_{j}}{dt}=-D_{j}+U_{j}+T_{j}+0.1\sum_{k}m({\bar{O}}_{k})W_{kj}^{OD},$$

where *U_j_* is an external reinforcer that inputs to the value category neuron during learning when the attended object is recognized and foveated:

(A67) $$U_{j}=\{\begin{array}{l} 0.2,\text{ if the }j^{th\;}\text{reinforcing input is on},\\ 0,\text{ otherwise}. \end{array}$$

For simplicity, it is assumed that the external reinforcer turns on with the object category to which it is associated, and shuts-off when the object category is reset. In the simulated network, the simultaneous presentation of object category and reinforcing input does not cause attentional blocking (Kamin, 1968, 1969) because the reinforcer is assumed not to have an object representation that can compete with the object to be conditioned. Conditioning works just as well if the reinforcing input turns on at a later time when the object category is still on (i.e., delay conditioning). For simulations of how blocking can occur in an extended network, see Grossberg and Levine (1987) and Dranias et al. (2008).

Variable *T_j_* in Equation (A66) is an internal drive input that projects to a specific value category. Among other functional roles, it can initiate a motivationally primed search:

(A68) $$T_{j}=\{\begin{array}{l} 0.2,\text{ if }j^{th}\text{ value category neuron receives internal drive},\\ 0,\text{ otherwise}; \end{array}$$

*m*(*O_k_*) is the output signal of the *k^th^* invariant object category neuron through the signal function in Equation (A21); and *W^OD^_kj_* is the weight defined in Equation (A78) from the *k^th^* invariant object category to the *j^th^* value category. The outputs of the value categories obey a normalized quenching competition:

(A69) $${\bar{D}}_{j}=\{\begin{array}{ll} D_{j}, & \begin{array}{l} \text{if }D_{j}=\max_{k}(D_{k}),;\\ \text{and }D_{j}>D_{k}+0.1,\;k\neq j, \end{array}\\ D_{j}\left(\frac{D_{j}}{\sum_{k\in \;\Omega }D_{k}}\right), & \begin{array}{l} \text{if }\left|D_{j}-D_{k}\right|\leq 0.1,\;k\in \Omega ,\;\\ \text{and }{\text{min}}_{j\in \;\Omega }(D_{j})>\max_{k\notin \;\Omega }(D_{k}+0.1), \end{array}\\ 0,\; & \begin{array}{l} \text{if }j\in \Omega . \end{array} \end{array}$$

#### C.5. Object-value categories

Object-value category neuron representations receive a driving bottom-up input from the corresponding invariant object category, a basal ganglia volition signal G that is turned on during volitional searches, and modulatory incentive motivational inputs from value categories (Baxter et al., 2000; Schoenbaum et al., 2003) as well as from name categories. The activity *F_j_* of the*j^th^* object-value category neuron obeys:

(A70) $$\begin{array}{l} \frac{1}{20}\frac{dF_{j}}{dt}=-F_{j}+\left(0.5m({\bar{O}}_{j})W_{j}^{OF}+G\right)\\ \;\left(1+\sum_{k}{\bar{D}}_{k}W_{kj}^{DF}+\sum_{i}m({\bar{N}}_{i})W_{ij}^{NF}\right), \end{array}$$

where *m*(*O_j_*) is the *j^th^* invariant object category output signal function *m*(*a*) defined in Equation (A21). The adaptive weights *W^OF^_j_* defined in Equation (A76) are strengthened by presentations of the invariant object category during reinforcement learning trials. Also in Equation (A70), *G* is the volition control signal defined in Equation (A62) which is turned on with a constant value equal to 0.1 when a top-down primed search is initiated, *D_k_* is the *k^th^* value category output, *W^DF^_kj_* is the adaptive weight defined in Equation (A79) from the *k^th^* value category neuron to the *j^th^* object-value category, *m*(*N_i_*) is the output signal of the *i^th^* name category representation through signal function *m*(*a*), and *W^NF^_ij_* is the adaptive weight defined in Equation (A81) from the *i^th^* name category to the *j^th^* object-value The outputs from object-value categories carry out a normalized quenching competition:

(A71) $${\bar{F}}_{j}=\{\begin{array}{ll} F_{j}, & \text{if }F_{j}=\max_{k}(F_{k}),\text{ and}\\ & \;F_{j}>F_{k}+0.5,\;k\neq j,\\ F_{j}\left(\frac{F_{j}}{\sum_{k\in \;\Omega }F_{k}}\right), & \text{if }\left|F_{j}-F_{k}\right|\leq 0.5,\;k\in \Omega ,\text{ and}\\ & \;{\text{min}}_{j\in \;\Omega }(F_{j})>\max_{k\notin \;\Omega }(F_{k}+0.5),\\ 0, & \text{if }j\in \Omega . \end{array}$$

The object-value category output signals generate modulatory top-down signals that amplify the activity of the corresponding invariant object category, as in Equation (A63), and bottom-up signals to activate name categories. During primed searches, object-value categories receive modulatory priming inputs from either name categories or value categories, as well as volitional signals that enable these modulatory inputs to fully activate their targeted cells and to thereby enable them to drive a search.

#### C.6. Name categories

The top What stream layer in the ARTSCAN Search model codes name categories. During training, each name category neuron receives inputs from object-value category representations and learns to be associated with the active object-value category neuron and generates feedback to strengthen the activity of this object-value category. During cognitively primed search, a particular name category receives a priming signal to initiate a search. The *j^th^* name category neuron activity*N_j_* obeys:

(A72) $$\frac{1}{20}\frac{dN_{j}}{dt}=-N_{j}+\sum_{i}m({\bar{F}}_{j})W_{ij}^{FN}+P_{j},$$

where *F_j_* is the output signal of the *j^th^* object-value category, *W^FN^_ij_* is the adaptive weight defined in Equation (A80) from the *i^th^* object-value category neuron to the *j^th^* name category neuron, and *P_j_* is the top-down priming signal that activates a specific name category to initiate a cognitively primed search:

(A73) $$P_{j}=\{\begin{array}{ll} 0.5, & \text{if }j^{th}\text{ name category neuron receives a},\\ & \text{ priming signal}\\ 0, & \text{otherwise}. \end{array}$$

The outputs from the name categories compete to select the maximally-activated name:

(A74) $${\bar{N}}_{j}=\{\begin{array}{ll} N_{j}, & \text{if }N_{j}=\max_{k}(N_{k}),\text{ and}\\ & \;N_{j}>N_{k}+0.1,\;k\neq j,\\ N_{j}\left(\frac{N_{j}}{\sum_{k\in \;\Omega }N_{k}}\right), & \text{if }\left|N_{j}-N_{k}\right|\leq 0.1,\;k\in \Omega ,\;\\ & \text{ and }{\text{min}}_{j\in \;\Omega }(N_{j})>\max_{k\notin \;\Omega }\\ & \;(N_{k}+0.1),\\ 0, & \text{if }j\in \Omega . \end{array}$$

#### C.7. What stream learning

The model employs two basic weight learning rules. One obeys the activity-gated steepest-descent *outstar* learning rule (Grossberg, 1980b) where learning is gated by a presynaptic signal and synaptic weights learn about postsynaptic activity. The connections from invariant category cells to object-value category cells Equation (A76) and from object-value category cells to name category cells Equation (A80) obey an *outstar* learning rule. The other learning processes obey a *doubly-gated outstar* learning rule (Grossberg and Merrill, 1992; Grossberg et al., 2002). Doubly-gated learning is gated by both presynaptic and postsynaptic neural activities, so that if either gate is inactive, the weight between them does not change. When both gates are active, the adaptive weight tracks the target signal by steepest descent. Doubly-gated learning includes the connections from invariant object categories to view category integrators in Equation (A75), from object-value categories to invariant object categories in Equation (A77), from invariant object categories to value categories in Equation (A78), from value categories to object-value categories in Equation (A79), and from name categories to object-value categories in Equation (A81).

(A75) $$\frac{50}{1}\frac{dW_{ij}^{OV,q}}{dt}=m({\bar{O}}_{i}){\left[V_{j}^{I,q}\right]}^{+}\left({\left[V_{j}^{I,q}\right]}^{+}-W_{ij}^{OV,q}\right),$$

(A76) $$\frac{50}{1}\frac{dW_{j}^{OF}}{dt}=m({\bar{O}}_{j})\left(m({\bar{F}}_{j})-W_{j}^{OF}\right),$$

(A77) $$\frac{50}{1}\frac{dW_{j}^{FO}}{dt}=m({\bar{F}}_{j})m({\bar{O}}_{j})\left(m({\bar{O}}_{j})-W_{j}^{FO}\right),$$

(A78) $$\frac{50}{1}\frac{dW_{ij}^{OD}}{dt}=m({\bar{O}}_{i})m({\bar{D}}_{j})\left(m({\bar{D}}_{j})-W_{ij}^{OD}\right),$$

(A79) $$\frac{50}{1}\frac{dW_{ij}^{DF}}{dt}=m({\bar{D}}_{i})m({\bar{F}}_{j})\left(m({\bar{F}}_{j})-W_{ij}^{DF}\right),$$

(A80) $$\frac{50}{1}\frac{dW_{ij}^{FN}}{dt}=m({\bar{F}}_{i})\left(m({\bar{N}}_{j})-W_{ij}^{FN}\right),$$

(A81) $$\frac{50}{1}\frac{dW_{ij}^{NF}}{dt}=m({\bar{N}}_{i})m({\bar{F}}_{j})\left(m({\bar{F}}_{j})-W_{j}^{NF}\right),$$

The output signals *O*, *D*, *F*, and *N* come from invariant, value, object-value, and name categories, respectively. The sigmoid signal function *m*(*a*) is defined in Equation (A21). *W^OV,q^_ij_* is the modulatory adaptive weight from the *i^th^* invariant object category neuron to the *j^th^* view category integrator in position *q*, *W^OF^_j_* is the weight from the *j^th^* invariant object category to the corresponding object-value category, *W^FO^_j_* is the modulatory adaptive weight from the *j^th^* object-value category to the corresponding invariant object category, *W^OD^_ij_* is the weight from the *i^th^* invariant object category to the *j^th^* value category, *W^DF^_ij_* is the modulatory weight from the *i^th^* value category to the j^th^ object-value category, *W^FN^_ij_* is the weight from the *i^th^* object-value category to the *j^th^* name category, and *W^NF^_ij_* is the modulatory adaptive weight from the *i^th^* name category to the *j^th^* object-value category.

### D. Top-down attentional primed search

ARTSCAN Search proposes that different top-down pathways from the What stream to the Where stream can achieve a Where’s Waldo search. A top-down primed search can be initiated either when a name category in Equation (A72) receives a priming signal in Equation (A73) or when a value category in Equation (A66) receives an internal motivational drive signal in Equation (A68), hereby priming the associated object-value category in Equation (A70). When such a prime occurs when a volitional signal in Equation (A62) is active, it can fire the corresponding invariant object category in Equation (A63) and then attentively primes, via the associated view category integration categories in Equation (A61), the view-specific categories in Equation (A55) at multiple positions. The most highly activated view-specific category can trigger an eye movement in Equation (A43) toward the desired Waldo target via a direct or an indirect route, respectively.

A value category may prime more than one object-value category if multiple objects are associated with this value category during reinforcement learning. As noted in section 7.3.7, if all the primed object-value categories have equal or similar motivational salience, then all the primed object-value categories can prime the corresponding invariant object categories because the output competition from the object-value categories in Equation (A71) will have approximately equal responses as a result of the normalized quenching competition. All the primed invariant object categories can then prime the corresponding view-specific categories across all positions. In our search examples, just one Waldo object is in a search scene. Whether the top-down prime, or the bottom-up Waldo input, occurs first, when these bottom-up and top-down signals are matched at the primed view-specific category, its activity is enhanced relative to the activities of other view-specific categories and thus can win the output competition from the view-specific categories because of the winner-take-all properties of the normalized quenching competition when one activity is sufficiently big relative to the others.

This choice can then drive where the eyes will next look via either the direct or indirect routes. The direct route (Figures 1B, 6A,C) activates the eye movement map Equation (A43) directly from the view-specific categories Equation (A55). The eye movement map can then make a winner-take-all choice based upon the position of the value-enhanced category. The indirect route (Figures 1C, 6B,D) uses the property that the competition among view-specific categories enables the primed view-specific category to win Equation (A60) after the view-specific categories receive their top-down priming signals. As a result, just the winning view-specific category can enhance the activation of its boundary representation Equation (A19). The boundary representation, in turn, can thereby increase contrast of its object surface through the surface filling-in process Equations (A22, A23). The enhanced surface representation projects to the spatial attention map Equation (A37) to win its competition through a surface-shroud resonance and its surface contour Equation (A27) is thereby enhanced. This winning shroud thus draws spatial attention as the largest hot spot on the enhanced surface contour determines an eye movement Equation (A43) to the target.

## References

[B1] Aggleton J. P. (1993). The contribution of the amygdala to normal and abnormal emotional states. Trends Neurosci. 16, 328–333 10.1016/0166-2236(93)90110-8 7691009

[B2] Andersen R. A. Bracewell R. M. Barash S. Gnadt J. W. Fogassi L. (1990). Eye position effects on visual, memory, and saccade-related activity in areas LIP and 7a of macaque. J. Neurosci. 10, 1176–1196 2329374 10.1523/JNEUROSCI.10-04-01176.1990 PMC6570201

[B3] Andersen R. A. Essick G. K. Siegel R. M. (1985). Encoding of spatial location by posterior parietal neurons. Science 230, 456–458 10.1126/science.4048942 4048942

[B4] Andersen R. A. Mountcastle V. B. (1983). The influence of the angle of gaze upon the excitability of the light- sensitive neurons of the posterior parietal cortex. J. Neurosci. 3, 532–548 6827308 10.1523/JNEUROSCI.03-03-00532.1983 PMC6564545

[B5] Baloch A. A. Waxman A. M. (1991). Visual learning, adaptive expectations, and behavioral conditioning of the mobile robot MAVIN. Neural Netw. 4, 271–302 10.1016/0893-6080(91)90067-F

[B6] Bar M. (2003). A cortical mechanism for triggering top-down facilitation in visual object recognition. J. Cogn. Neurosci. 15, 600–609 10.1162/089892903321662976 12803970

[B7] Barbas H. (1995). Anatomic basis of cognitive-emotional interactions in the primate prefrontal cortex. Neurosci. Biobehav. Rev. 19, 499–510 10.1016/0149-7634(94)00053-4 7566750

[B8] Barbas H. (2000). Complementary roles of prefrontal cortical regions in cognition, memory, and emotion in primates. Adv. Neurol. 84, 87–110 11091860

[B9] Barceló F. Suwazono S. Knight R. T. (2000). Prefrontal modulation of visual processing in humans. Nat. Neurosci. 3, 399–403 10.1038/73975 10725931

[B10] Basu A. Licardie S. (1993). Modeling fish-eye lenses, in Proceedings of the IEEE/RSJ International Conference on Intelligent Robots and Systems (Yokohama), 1822–1828

[B11] Baxter M. G. Parker A. Lindner C. C. C. Izquierdo A. D. Murray E. A. (2000). Control of response selection by reinforcer value requires interaction of amygdala and orbital prefrontal cortex. J. Neurosci. 20, 4311–4319 10818166 10.1523/JNEUROSCI.20-11-04311.2000 PMC6772657

[B12] Bechara A. Tranel D. Damasio H. Damasio A. R. (1996). Failure to respond autonomically to anticipated future outcomes following damage to prefrontal cortex. Cereb. Cortex 6, 215–225 10.1093/cercor/6.2.215 8670652

[B13] Bischoff-Grethe A. Proper S. M. Mao H. Daniels K. A. Berns G. S. (2000). Conscious and unconscious processing of nonverbal predictability in Wernicke’s area. J. Neurosci. 20, 1975–1981 10684898 10.1523/JNEUROSCI.20-05-01975.2000 PMC6772930

[B14] Booth M. C. Rolls E. T. (1998). View-invariant representations of familiar objects by neurons in the inferior temporal visual cortex. Cereb. Cortex 8, 510–523 10.1093/cercor/8.6.510 9758214

[B15] Borg-Graham L. J. Monier C. Fregnac Y. (1998). Visual input evokes transient and strong shunting inhibition in visual cortical neurons. Nature 393, 369–373 10.1038/30735 9620800

[B16] Bradski G. Grossberg S. (1995). Fast learning VIEWNET architectures for recognizing 3-D objects from multiple 2-D views. Neural Netw. 8, 1053–1080 10.1016/0893-6080(95)00053-4

[B16a] Brown J. M. Denney H. I. (2007). Shifting attention into and out of objects: evaluating the processes underlying the object advantage. Percept. Psychophys. 69, 606–618 17727114 10.3758/bf03193918

[B17] Brunel N. (2003). Dynamics and plasticity of stimulus selective persistent activity in cortical network models. Cereb. Cortex 13, 1151–1161 10.1093/cercor/bhg096 14576207

[B18] Bülthoff H. H. Edelman S. (1992). Psychophysical support for a two-dimensional view interpolation theory of object recognition. Proc. Natl. Acad. Sci. U.S.A. 89, 60–64 10.1073/pnas.89.1.60 1729718 PMC48175

[B19] Bülthoff H. H. Edelman S. Y. Tarr M. J. (1995). How are three-dimensional objects represented in the brain? Cereb. Cortex 5, 247–260 10.1093/cercor/5.3.247 7613080

[B20] Cao Y. Grossberg S. (2005). A laminar cortical model of stereopsis and 3D surface perception: Closure and da Vinci stereopsis. Spat. Vis. 18, 515–578 10.1163/156856805774406756 16312095

[B21] Cao Y. Grossberg S. (2012). Stereopsis and 3D surface perception by spiking neurons in laminar cortical circuits: a method of converting neural rate models into spiking models. Neural Netw. 26, 75–98 10.1016/j.neunet.2011.10.010 22119530

[B22] Cao Y. Grossberg S. Markowitz J. (2011). How does the brain rapidly learn and reorganize view- and positionally-invariant object representations in inferior temporal cortex? Neural Netw. 24, 1050–1061 10.1016/j.neunet.2011.04.004 21596523

[B23] Caplovitz G. P. Tse P. U. (2007). Rotating dotted ellipses: Motion perception driven by grouped figural rather than local dot motion signals. Vision Res. 47, 1979–1991 10.1016/j.visres.2006.12.022 17548102

[B24] Carlsson K. Petrovic P. Skare S. Petersson K. M. Ingvar M. (2000). Tickling expectations: neural processing in anticipation of a sensory stimulus. J. Cogn. Neurosci. 12, 691–703 10.1162/089892900562318 10936920

[B25] Carpenter G. A. Grossberg S. (1981). Adaptation and transmitter gating in vertebrate photoreceptors. J. Theor. Neurobiol. 1, 1–42 (Reprinted in *The Adaptive Brain,* Vol. 2, ed Grossberg S. Amsterdam: Elsevier).

[B26] Carpenter G. A. Grossberg S. (1987). A massively parallel architecture for a self-organizing neural pattern-recognition machine. Comp. Vis. Graph. Image Process. 37, 54–115 10.1016/S0734-189X(87)80014-212662581

[B27] Carpenter G. A. Grossberg S. (1991). Pattern Recognition by Self-Organizing Neural Networks. Cambridge, MA: MIT Press

[B28] Carpenter G. A. Grossberg S. Markuzon N. Reynolds J. H. Rosen D. B. (1992). Fuzzy ARTMAP: a neural network architecture for incremental supervised learning of analog multidimensional maps. IEEE Trans. Neural Netw. 3, 698–713 10.1109/72.159059 18276469

[B29] Carpenter G. A. Grossberg S. Rosen D. B. (1991). Fuzzy ART: fast stable learning and categorization of analog patterns by an adaptive resonance system. Neural Netw. 4, 759–771 10.1016/0893-6080(91)90056-B

[B30] Carpenter G. A. Ross W. D. (1995). ART-EMAP: a neural network architecture for object recognition by evidence accumulation. IEEE Trans. Neural Netw. 6, 805–818 10.1109/72.392245 18263371

[B31] Carrasco M. Penpeci-Talgar C. Eckstein M. (2000). Spatial covert attention increases contrast sensitivity across the CSF: support for signal enhancement. Vision Res. 40, 1203–1215 10.1016/S0042-6989(00)00024-9 10788636 PMC3825514

[B32] Cassanello C. R. Ferrera V. P. (2007). Visual remapping by vector subtraction: analysis of multiplicative gain field models. Neural Comput. 19, 2353–2386 10.1162/neco.2007.19.9.2353 17650063

[B33] Cavada C. Tejedor J. Cruz-Rizzolo R. J. Reinoso-Suárez F. (2000). The anatomical connections of the macaque monkey orbitofrontal cortex. A review. Cereb. Cortex 10, 220–242 10.1093/cercor/10.3.220 10731218

[B34] Cavanagh P. Hunt A. R. Alfraz A. Rolfs M. (2010). Visual stability based on remapping of attention pointers. Trends Cogn. Sci. 14, 147–153 10.1016/j.tics.2010.01.007 20189870 PMC2847621

[B35] Chang H.-C. Cao Y. Grossberg S. (2009a). Where’s Waldo? How multiple perceptual, cognitive, and emotional brain regions cooperate during learning to categorize and find desired objects in a cluttered scene. Soc. Neurosci. 503, 12 10.3389/fnint.2014.00043 PMC4060746 24987339

[B36] Chang H.-C. Cao Y. Grossberg S. (2009b). Where’s Waldo? How the brain learns to categorize and discover desired objects in a cluttered scene. J. Vis. 9:173 10.1167/9.8.173 PMC4060746 24987339

[B37] Chang H.-C. Cao Y. Grossberg S. (2013). Where’s Waldo? How multiple perceptual, cognitive, and emotional brain regions cooperate during learning to categorize and find desired objects in a cluttered scene, in International Conference on Cognitive and Neural Systems (Boston, MA). 10.3389/fnint.2014.00043 PMC4060746 24987339

[B38] Chiu Y.-C. Yantis S. (2009). A domain-independent source of cognitive control for task sets: Shifting spatial attention and switching categorization rules. J. Neurosci. 29, 3930–3938 10.1523/JNEUROSCI.5737-08.2009 19321789 PMC2817948

[B39] Chun M. M. Jiang Y. (1998). Contextual cueing: implicit learning and memory of visual context guides spatial attention. Cogn. Psychol. 36, 28–71 10.1006/cogp.1998.0681 9679076

[B40] Cohen M. A. Grossberg S. (1984). Neural dynamics of brightness perception: features, boundaries, diffusion, and resonance. Percept. Psychophys. 36, 428–456 10.3758/BF03207497 6398424

[B41] Colby C. L. Duhamel J. R. Goldberg M. E. (1993). The analysis of visual space by the lateral intraparietal area of the monkey: the role of extraretinal signals. Prog. Brain Res. 95, 307–316 10.1016/S0079-6123(08)60378-7 8493341

[B42] Damasio A. R. (1999). The Feeling of What Happens: Body and Emotion in the Making of Consciousness. New York, NY: Harcourt Brace

[B43] Deneve S. Pouget A. (2003). Basis functions for object-centered representations. Neuron 37, 347–359 10.1016/S0896-6273(02)01184-4 12546828

[B44] Desimone R. Gross C. G. (1979). Visual areas in the temporal cortex of the macaque. Brain Res. 178, 363–380 10.1016/0006-8993(79)90699-1 116712

[B45] Downing C. J. (1988). Expectancy and visual-spatial attention: effects on perceptual quality. J. Exp. Psychol. Hum. Percept. Perform. 14, 188–202 10.1037/0096-1523.14.2.188 2967876

[B46] Dranias M. Grossberg S. Bullock D. (2008). Dopaminergic and non-dopaminergic value systems in conditioning and outcome-specific revaluation. Brain Res. 1238, 239–287 10.1016/j.brainres.2008.07.013 18674518

[B47] Duhamel J.-R. Colby C. L. Goldberg M. E. (1992). The updating of the representation of visual space in parietal cortex by intended eye movements. Science 255, 90–92 10.1126/science.1553535 1553535

[B48] Eriksen C. W. Yeh Y. Y. (1985). Allocation of attention in the visual field. J. Exp. Psychol. Hum. Percept. Perform. 11, 583–597 10.1037/0096-1523.11.5.583 2932532

[B49] Erkelens C. J. Hooge I. T. C. (1996). The role of peripheral vision in visual search. J. Videol. 1, 1–8

[B50] Fang L. Grossberg S. (2009) From stereogram to surface: how the brain sees the world in depth. Spat. Vis. 22, 45–82 10.1163/156856809786618484 19055887

[B51] Fazl A. Grossberg S. Mingolla E. (2009). View-invariant object category learning, recognition, and search: how spatial and object attention are coordinated using surface-based attentional shrouds. Cogn. Psychol. 58, 1–48 10.1016/j.cogpsych.2008.05.001 18653176

[B52] Fecteau J. H. Munoz D. P. (2006). Salience, relevance, and firing: a priority map for target selection. Trends Cogn. Sci. 10, 617–631 10.1016/j.tics.2006.06.011 16843702

[B53] Foley N. C. Grossberg S. Mingolla E. (2012). Neural dynamics of object-based multifocal visual spatial attention and priming: object cueing, useful-field-of-view, and crowding. Cogn. Psychol. 65, 77–117 10.1016/j.cogpsych.2012.02.001 22425615 PMC4784991

[B54] Frith C. Dolan R. J. (1997). Brain mechanisms associated with top-down processes in perception. Philos. Trans. Roy. Soc. Lond. B Biol. Sci. 352, 1221–1230 10.1098/rstb.1997.0104 9304688 PMC1692001

[B55] Fukushima K. (1980). Neocognitron: a self-organizing neural network model for a mechanism of pattern recognition unaffected by shift in position. Biol. Cybern. 36, 193–202 10.1007/BF00344251 7370364

[B56] Fukushima K. (1986). Neocognitron: a hierarchical neural network capable of visual pattern recognition. Neural Netw. 1, 119–130 10.1016/0893-6080(88)90014-7

[B57] Fuster J. M. Jervey J. P. (1981). Inferotemporal neurons distinguish and retain behaviorally relevant features of visual stimuli. Science 212, 952–955 10.1126/science.7233192 7233192

[B58] Gancarz G. Grossberg G. (1999). A neural model of the saccadic eye movement control explains task-specific adaptation. Vision Res. 39, 3123–3143 10.1016/S0042-6989(99)00049-8 10664809

[B59] Goldberg M. E. Bruce C. J. (1990). Primate frontal eye fields. III. Maintenance of a spatially accurate saccade signal. J. Neurophysiol. 64, 489–508 2213128 10.1152/jn.1990.64.2.489

[B60] Goodale M. A. Milner A. D. (1992). Separate visual pathways for perception and action. Trends Neurosci. 15, 20–25 10.1016/0166-2236(92)90344-8 1374953

[B61] Gottlieb J. P. Kusunoki M. Goldberg M. E. (1998). The representation of visual salience in monkey parietal cortex. Nature 391, 481–484 10.1038/35135 9461214

[B62] Gross C. G. Rocha-Miranda C. E. Bender D. B. (1972). Visual properties of neurons in inferotemporal cortex of the Macaque. J. Neurophysiol. 35, 96–111 4621506 10.1152/jn.1972.35.1.96

[B63] Grossberg S. (1971). On the dynamics of operant conditioning. J. Theor. Biol. 33, 225–255 10.1016/0022-5193(71)90064-6 4332676

[B64] Grossberg S. (1972a). A neural theory of punishment and avoidance, I: qualitative theory. Math. Biosci. 15, 39–67 10.1016/0025-5564(72)90062-4

[B65] Grossberg S. (1972b). A neural theory of punishment and avoidance, II: quantitative theory. Math. Biosci. 15, 253–285 10.1016/0025-5564(72)90038-7

[B66] Grossberg S. (1973). Contour enhancement, short-term memory, and constancies in reverberating neural networks. Stud. Appl. Math. 52, 213–257

[B67] Grossberg S. (1975). A neural model of attention, reinforcement, and discrimination learning. Int. Rev. Neurobiol. 18, 263–327 10.1016/S0074-7742(08)60037-9 1107246

[B68] Grossberg S. (1980a). Biological competition: decision rules, pattern formation, and oscillations. Proc. Natl. Acad. Sci. U.S.A. 77, 2338–2342 10.1073/pnas.77.4.2338 16592807 PMC348710

[B69] Grossberg S. (1980b). How does a brain build a cognitive code? Psychol. Rev. 87, 1–51 10.1037/0033-295X.87.1.1 7375607

[B70] Grossberg S. (1982). Processing of expected and unexpected events during conditioning and attention: a psychophysiological theory. Psychol. Rev. 89, 529–572 10.1037/0033-295X.89.5.529 7178332

[B71] Grossberg S. (1983). The quantized geometry of visual space: the coherent computation of depth, form, and lightness. Behav. Brain Sci. 6, 625–692 10.1017/S0140525X00017763

[B72] Grossberg S. (1984). Some psychophysiological and pharmacological correlates of a developmental, cognitive and motivational theory. Ann. N.Y. Acad. Sci. 425, 58–151 10.1111/j.1749-6632.1984.tb23523.x 6146280

[B73] Grossberg S. (1988) Nonlinear neural networks: principles, mechanisms, and architectures. Neural Netw. 1, 17–61 10.1016/0893-6080(88)90021-418579344

[B74] Grossberg S. (1994). 3-D vision and figure-ground separation by visual cortex. Percept. Psychophys. 55, 48–121 10.3758/BF03206880 8036093

[B75] Grossberg S. (1997). Cortical dynamics of three-dimensional figure-ground perception of two-dimensional figures. Psychol. Rev. 104, 618–658 10.1037/0033-295X.104.3.618 9243966

[B76] Grossberg S. (1999). How does the cerebral cortex work? Learning, attention and grouping by the laminar circuits of visual cortex. Spatial Vis. 12, 163–186 10.1163/156856899X00102 10221426

[B77] Grossberg S. (2000). How hallucinations may arise from brain mechanisms of learning, attention, and volition. J. Int. Neuropsychol. Soc. 6, 579–588 10.1017/S135561770065508X 10932478

[B78] Grossberg S. (2007). Consciousness CLEARS the mind. Neural Netw. 20, 1040–1053 10.1016/j.neunet.2007.09.014 17964756

[B79] Grossberg S. (2009). Cortical and subcortical predictive dynamics and learning during perception, cognition, emotion, and action. Philos. Trans. R. Soc. Lond. B Biol. Sci. 364, 1223–1234 10.1098/rstb.2008.0307 19528003 PMC2666707

[B80] Grossberg S. (2012). Adaptive resonance theory: how a brain learns to consciously attend, recognize, and predict a changing world. Neural Netw. 37, 1–47 10.1016/j.neunet.2012.09.017 23149242

[B81] Grossberg S. (2013a). Adaptive Resonance Theory. Scholarpedia. Available online at: http://www.scholarpedia.org/article/Adaptive_resonance_theory

[B82] Grossberg S. (2013b). Recurrent Neural Networks. Scholarpedia. Available online at: http://www.scholarpedia.org/article/Recurrent_neural_networks

[B83] Grossberg S. Bullock D. Dranias M. (2008). Neural dynamics underlying impaired autonomic and conditioned responses following amygdala and orbitofrontal lesions. Behav. Neurosci. 122, 1100–1125 10.1037/a0012808 18823167

[B84] Grossberg S. Hong S. (2006). A neural model of surface perception: lightness, anchoring, and filling-in. Spat. Vis. 19, 263–321 10.1163/156856806776923399 16862842

[B85] Grossberg S. Howe P. D. L. (2003). A laminar cortical model of stereopsis and three-dimensional surface perception. Vision Res. 43, 801–829 10.1016/S0042-6989(03)00011-7 12639606

[B86] Grossberg S. Hwang S. Mingolla E. (2002). Thalamocortical dynamics of the McCollough effect: boundary-surface alignment through perceptual learning. Vision Res. 42, 1259–1286 10.1016/S0042-6989(02)00055-X 12044758

[B87] Grossberg S. Kelly F. J. (1999). Neural dynamics of binocular brightness perception. Vision Res. 39, 3796–3816 10.1016/S0042-6989(99)00095-4 10746149

[B88] Grossberg S. Kuperstein M. (1986). Neural Dynamics of Adaptive Sensory-Motor Control: Ballistic Eye Movements. Amsterdam: Elsevier Science

[B89] Grossberg S. Kuperstein M. (1989). Neural Dynamics of Adaptive Sensory-Motor Control: Expanded Edition. Elmsford, NY: Pergamon Press

[B90] Grossberg S. Levine D. S. (1987). Neural dynamics of attentionally modulated Pavlovian conditioning: blocking, inter-stimulus interval, and secondary reinforcement. Appl. Opt. 26, 5015–5030 10.1364/AO.26.005015 20523481

[B91] Grossberg S. McLoughlin N. (1997). Cortical dynamics of 3-D surface perception: binocular and half-occluded scenic images. Neural Netw. 10, 1583–1605 10.1016/S0893-6080(97)00065-8

[B92] Grossberg S. Merrill J. W. L. (1992). A neural network model of adaptively timed reinforcement learning and hippocampal dynamics. Cogn. Brain Res. 1, 3–38 10.1016/0926-6410(92)90003-A 15497433

[B93] Grossberg S. Mingolla E. (1985). Neural dynamics of perceptual grouping: textures, boundaries, and emergent segmentations. Percept. Psychophys. 38, 141–171 10.3758/BF03198851 4088806

[B94] Grossberg S. Mingolla E. Ross (1994). A neural theory of attentive visual search: Interactions of boundary, surface, spatial, and object representations. Psychol. Rev. 101, 470–489 10.1037/0033-295X.101.3.470 7938340

[B95] Grossberg S. Raizada R. D. (2000). Contrast-sensitive perceptual grouping and object-based attention in the laminar circuits of primary visual cortex. Vision Res. 40, 1413–1432 10.1016/S0042-6989(99)00229-1 10788649

[B96] Grossberg S. Schmajuk N. A. (1987). Neural dynamics of attentionally-modulated Pavlovian conditioning: conditioned reinforcement, inhibition, and opponent processing. Psychobiology 15, 195–240 20523481

[B97] Grossberg S. Seidman D. (2006). Neural dynamics of autistic behaviors: cognitive, emotional, and timing substrates. Psychol. Rev. 113, 483–525 10.1037/0033-295X.113.3.483 16802879

[B98] Grossberg S. Swaminathan G. (2004). A laminar cortical model for 3D perception of slanted and curved surfaces and of 2D images: development, attention, and bistability. Vision Res. 44, 1147–1187 10.1016/j.visres.2003.12.009 15050817

[B99] Grossberg S. Todorović D. (1988). Neural dynamics of 1D and 2D brightness perception: A unified model of classical and recent phenomena. Percept. Psychophys. 43, 241–277 10.3758/BF03207869 3347487

[B100] Grossberg S. Yazdanbakhsh A. (2005). Laminar cortical dynamics of 3D surface perception: stratification, transparency, and neon color spreading. Vision Res. 45, 1725–1743 10.1016/j.visres.2005.01.006 15792846

[B101] Heeger D. (1992). Normalization of cell responses in cat striate cortex. Vis. Neurosci. 9, 181–197 10.1017/S0952523800009640 1504027

[B102] Hirsch J. Alonso J. Reid R. Martinez L. (1998). Synaptic integration in striate cortical simple cells. J. Neurosci. 18, 9517–9528 9801388 10.1523/JNEUROSCI.18-22-09517.1998 PMC6792880

[B103] Hong S. Grossberg S. (2004). A neuromorphic model for achromatic and chromatic surface representation of natural images. Neural Netw. 17, 787–808 10.1016/j.neunet.2004.02.007 15288898

[B104] Huang T.-R. Grossberg S. (2010). Cortical dynamics of contextually cued attentive visual learning and search: spatial and object evidence accumulation. Psychol. Rev. 117, 1080–1112 10.1037/a0020664 21038974

[B105] Hubel D. H. Wiesel T. N. (1959). Receptive fields of single neurones in the cat’s striate cortex. J. Physiol. 148, 574–591 14403679 10.1113/jphysiol.1959.sp006308 PMC1363130

[B106] Hubel D. H. Wiesel T. N. (1962). Receptive fields, binocular interaction and functional architecture in the cat’s visual cortex. J. Physiol. 160, 106 14449617 10.1113/jphysiol.1962.sp006837 PMC1359523

[B107] Hung C. P. Kreiman G. Poggio T. DiCarlo J. J. (2005) Fast read-out of object identity from macaque inferior temporal cortex. Science 310, 863–866 10.1126/science.1117593 16272124

[B108] Hyle M. Vasan N. Butcher S. Wolfe J. (2002). How fast can you change your mind? Effects of target identity cues in visual search. J. Vis. 2, 534–534 10.1167/2.7.53415066400

[B109] Ito M. Tamura H. Fujita I. Tanaka K. (1995). Size and position invariance of neuronal responses in monkey inferotemporal cortex. J. Neurophysiol. 73, 218–226 7714567 10.1152/jn.1995.73.1.218

[B110] Itti L. Koch C. (2001). Computational modelling of visual attention. Nat. Rev. Neurosci. 2, 194–203 10.1038/35058500 11256080

[B111] Jonides J. Irwin D. E. Yantis S. (1982). Integrating visual information from successive fixations. Science 215, 192–194 10.1126/science.7053571 7053571

[B112] Kamin L. J. (1968). Attention-like processing in classical conditioning, in Miami Symposium on the Prediction of Behavior: Aversive Stimulations. ed Jones M. R. (Coral Gables, FL: University of Miami Press), 9–31

[B113] Kamin L. J. (1969). Predictability, surprise, attention, and conditioning, in Punishment and Aversive Behavior, eds Campbell B. A. Church R. M. (New York, NY: Appleton-Century-Crofts), 279–296

[B114] Kanan C. Cottrell G. W. (2010). Robust classification of objects, faces, and flowers using natural image statistics, in IEEE Conference on Computer Vision and Pattern Recognition (San Francisco, CA), 2472–2479

[B115] Kelly F. Grossberg S. (2000). Neural dynamics of 3-D surface perception: figure-ground separation and lightness perception. Percept. Psychophys. 62, 1596–1618 10.3758/BF03212158 11140181

[B116] Krieger G. Rentschler I. Hauske G. Schill K. Zetzsche C. (2000). Object and scene analysis by saccadic eye-movements: an investigation with higher-order statistics. Spat. Vis. 13, 201–214 10.1163/156856800741216 11198232

[B117] Kringelbach M. L. (2005). The human orbitofrontal cortex: linking reward to hedonic experience. Nat. Rev. Neurosci. 6, 691–702 10.1038/nrn1747 16136173

[B118] Kristjánsson Á. Wang D. Nakayama K. (2002). The role of priming in conjunctive visual search. Cognition 85, 37–52 10.1016/S0010-0277(02)00074-4 12086712

[B119] LeDoux J. E. (1993). Emotional memory systems in the brain. Behav. Brain Res. 58, 69–79 10.1016/0166-4328(93)90091-4 8136051

[B120] Li N. DiCarlo J. J. (2008). Unsupervised natural experience rapidly alters invariant object representation in visual cortex. Science 321, 1502–1507 10.1126/science.1160028 18787171 PMC3307055

[B121] Liversedge S. P. Findlay J. M. (2000). Saccadic eye movements and cognition. Trends Cogn. Sci. 4, 6–14 10.1016/S1364-6613(99)01418-7 10637617

[B122] Logothetis N. Pauls J. Bülthoff H. Poggio T. (1994). View-dependent object recognition by monkeys. Curr. Biol. 4, 401–414 10.1016/S0960-9822(00)00089-0 7922354

[B123] Mathôt S. Theeuwes J. (2010). Gradual remapping results in early retinotopic and late spatiotopic inhibition of return. Psychol. Sci. 21, 1793–1798 10.1177/0956797610388813 21078894

[B124] McMains S. A. Somers D. C. (2005). Processing efficiency of divided spatial attention mechanisms in human visual cortex. J. Neurosci. 25, 9444–9448 10.1523/JNEUROSCI.2647-05.2005 16221854 PMC6725695

[B125] Miller E. K. Erickson C. A. Desimone R. (1996). Neural mechanisms of visual working memory in prefrontal cortex of the macaque. J. Neurosci. 16, 5154–5167 8756444 10.1523/JNEUROSCI.16-16-05154.1996 PMC6579322

[B126] Mishkin M. Ungerleider L. G. Macko K. A. (1983). Object vision and spatial vision: two cortical pathways. Trends Neurosci. 6, 414–417 10.1016/0166-2236(83)90190-X

[B127] Mitchell J. F. Zipser D. (2003). Sequential memory-guided saccades and target selection: a neural model of the frontal eye fields. Vision Res. 43, 2669–2695 10.1016/S0042-6989(03)00468-1 14552808

[B128] Miyashita Y. Chang H. S. (1988). Neuronal correlate of pictorial short-term memory in the primate temporal cortex. Nature 331, 68–70 10.1038/331068a0 3340148

[B129] Miyashita Y. Hayashi T. (2000). Neural representation of visual objects: encoding and top-down activation. Curr. Opin. Neurobiol. 10, 187–194 10.1016/S0959-4388(00)00071-4 10753793

[B130] Müller H. J. Reimann B. Krummenacher J. (2003). Visual search for singleton feature targets across dimensions: Stimulus-and expectancy-driven effects in dimensional weighting. J. Exp. Psychol. Hum. Percept. Perform. 29, 1021 10.1037/0096-1523.29.5.1021 14585020

[B131] Muramoto K. Ono T. Nishijo H. Fukuda M. (1993). Rat amygdaloid neuron responses during auditory discrimination. Neuroscience 52, 621–636 10.1016/0306-4522(93)90411-8 8450963

[B132] Nakamura K. Colby C. L. (2000). Visual, saccade-related, and cognitive activation of single neurons in monkey extrastriate area V3A. J. Neurophysiol. 84, 677–692 10938295 10.1152/jn.2000.84.2.677

[B133] Nishijo H. Ono T. Nishino H. (1988). Topographic distribution of modality-specific amygdalar neurons in alert monkey. J. Neurosci. 8, 3556–3569 3193170 10.1523/JNEUROSCI.08-10-03556.1988 PMC6569600

[B134] Olson S. Grossberg S. (1998). A neural network model for the development of simple and complex cell receptive fields within cortical maps of orientation and ocular dominance. Neural Netw. 11, 189–208 10.1016/S0893-6080(98)00003-3 12662831

[B135] Pessoa L. Ungerleider L. G. (2004). Neuoimaging studies of attention and the processing of emotion-laden stimuli. Prog. Brain Res. 144, 171–182 10.1016/S0079-6123(03)14412-3 14650848

[B136] Petrides M. Alivisatos B. Frey S. (2002). Differential activation of the human orbital, mid-ventrolateral, and mid-dorsolateral prefrontal cortex during the processing of visual stimuli. Proc. Natl. Acad. Sci. U.S.A. 99, 5649–5654 10.1073/pnas.072092299 11960018 PMC122825

[B137] Pilly P. K. Grossberg S. (2012). How do spatial learning and memory occur in the brain? Coordinated learning of entorhinal grid cells and hippocampal place cells. J. Cogn. Neurosci. 24, 1031–1054 10.1162/jocn_a_00200 22288394

[B138] Posner M. I. (1980). Orienting of attention. Q. J. Exp. Psychol. 32, 3–25 10.1080/00335558008248231 7367577

[B139] Pouget A. Dayan P. Zemel R. S. (2003). Inference and computation with population codes. Annu. Rev. Neurosci. 26, 381–410 10.1146/annurev.neuro.26.041002.131112 12704222

[B140] Pouget A. Sejnowski T. (1997). Spatial transformations in the parietal cortex using basis functions. J. Cogn. Neurosci. 9, 222–237 10.1162/jocn.1997.9.2.222 23962013

[B141] Pouget A. Snyder L. H. (2000). Computational approaches to sensorimotor transformations. Nat. Neurosci. 3, 1192–1198 10.1038/81469 11127837

[B142] Pylyshyn Z. W. Storm R. W. (1988). Tracking multiple independnent targets: Evidence for a parallel tracking mechanism. Spat. Vis. 3, 179–197 10.1163/156856888X00122 3153671

[B143] Qiu F. T. von der Heydt R. (2005). Figure and ground in the visual cortex: V2 combines stereoscopic cues with Gestalt rules. Neuron 47, 155–166 10.1016/j.neuron.2005.05.028 15996555 PMC1564069

[B144] Raizada R. Grossberg S. (2001). Context-sensitive binding by the laminar circuits of V1 and V2: a unified model of perceptual grouping, attention and orientation contrast. Vis. Cogn. 8, 431–466 10.1080/13506280143000070

[B145] Ranganath C. DeGutis J. D’Esposito M. (2004). Category-specific modulation of inferior temporal activity during working memory encoding and maintenance. Cogn. Brain Res. 20, 37–45 10.1016/j.cogbrainres.2003.11.017 15130587

[B146] Reid R. Alonso J. (1995). Specificity of monosynaptic connections from thalamus to visual cortex. Nature 378, 281–284 10.1038/378281a0 7477347

[B147] Reynolds J. H. Chelazzi L. (2004). Attentional modulation of visual processing. Annu. Rev. Neurosci. 27, 611–647 10.1146/annurev.neuro.26.041002.131039 15217345

[B148] Reynolds J. H. Chelazzi L. Desimone R. (1999). Competitive mechanisms subserve attention in macaque areas V2 and V4. J. Neurosci. 19, 1736–1753 10024360 10.1523/JNEUROSCI.19-05-01736.1999 PMC6782185

[B149] Reynolds J. H. Desimone R. (2003). Interacting roles of attention and visual salience in V4. Neuron 37, 853–863 10.1016/S0896-6273(03)00097-7 12628175

[B150] Reynolds J. H. Pasternak T. Desimone R. (2000). Attention increases sensitivity of V4 neurons. Neuron 26, 703–714 10.1016/S0896-6273(00)81206-4 10896165

[B151] Riesenhuber M. Poggio T. (2000). Models of object recognition. Nat. Neurosci. 3(Suppl.), 1199–1204 10.1038/81479 11127838

[B152] Rolls E. T. (1999). The Brain and Emotion. Oxford: Oxford University Press

[B153] Rolls E. T. (2000). The orbitofrontal cortex and reward. Cereb. Cortex 10, 284–294 10.1093/cercor/10.3.284 10731223

[B154] Schoenbaum G. Setlow B. Saddoris M. P. Gallagher M. (2003). Encoding predicted outcome and acquired value in orbitofrontal cortex during cue sampling depends upon input from basolateral amygdala. Neuron 39, 855–867 10.1016/S0896-6273(03)00474-4 12948451

[B155] Schwartz E. L. (1980). Computational anatomy and functional architecture of striate cortex: a spatial mapping approach to perceptual coding. Vision Res. 20, 645–669 10.1016/0042-6989(80)90090-5 7445436

[B156] Schwartz O. Simoncelli E. P. (2001). Natural signal statistics and sensory gain control. Nat. Neurosci. 4,819–825 10.1038/90526 11477428

[B157] Seibert M. Waxman A. M. (1992). Adaptive 3-D object recognition from multiple views. IEEE Trans. Pattern Anal. Mach. Intell. 14, 107–124 10.1109/34.121784

[B158] Serences J. T. Yantis S. (2006) Selective visual attention and perceptual coherence. Trends Cogn. Sci. 10, 38–45 10.1016/j.tics.2005.11.008 16318922

[B159] Tanaka K. (1993). Neuronal mechanisms of object recognition. Science 262, 685–688 10.1126/science.8235589 8235589

[B160] Theeuwes J. Mathôt S. Kingstone A. (2010). Object-based eye movements: The eyes prefer to stay within the same object. Atten. Percept. Psychophys. 72, 12–21 10.3758/APP.72.3.597 20348565

[B161] Thorpe S. J. Gegenfurtner K. R. Fabre-Thorpe M. Bülthoff H. H. (2001). Detection of animals in natural images using far peripheral vision. Eur. J. Neurosci. 14, 869–876 10.1046/j.0953-816x.2001.01717.x 11576191

[B162] Tolias A. S. Moore T. Smirnakis S. M. Tehovnik E. J. Siapas A. G. Schiller P. H. (2001). Eye movements modulate visual receptive fields of V4 neurons. Neuron 29, 757–767 10.1016/S0896-6273(01)00250-1 11301034

[B163] Tomita H. Ohbayashi M. Nakahara K. Hasegawa I. Miyashita Y. (1999). Top-down signal from prefrontal cortex in executive control of memory retrieval. Nature 401, 699–703 10.1038/44372 10537108

[B164] Toyomitsu Y. Nishijo H. Uwano T. Kuratsu J. Ono T. (2002). Neuronal responses of the rat amygdala during extinction and reassociation learning in elementary and configural associative tasks. Eur. J. Neurosci. 15, 753–768 10.1046/j.1460-9568.2002.01889.x 11886454

[B165] Treisman A. M. Gelade G. (1980). A feature-integration theory of attention. Cogn. Psychol. 12, 97–136 10.1016/0010-0285(80)90005-5 7351125

[B166] Tyler C. W. Kontsevich L. L. (1995). Mechanisms of stereoscopic processing: stereoattention and surface perception in depth reconstruction. Perception 24, 127–153 10.1068/p240127 7617422

[B167] Ungerleider L. G. Mishkin M. (1982). Two cortical visual systems, in Analysis of Visual Behavior, eds Ingle D. J. Goodale M. A. Mansfield R. J. W. (Cambridge, MA: MIT Press), 549–586

[B168] Werblin F. S. (1971). Adaptation in a vertebrate retina: intracellular recordings in Necturus. J. Neurophysiol. 34, 228–241 5545938 10.1152/jn.1971.34.2.228

[B169] Wolfe J. M. (1994). Guided Search 2.0: a revised model of visual search. Psychon. Bull. Rev. 1, 202–238 10.3758/BF03200774 24203471

[B170] Wolfe J. M. Cave K. R. Franzel S. L. (1989). Guided Search: an alternative to the feature integration model for visual Search. J. Exp. Psychol. Hum. Percept. Perform. 15, 419–433 10.1037/0096-1523.15.3.419 2527952

[B171] Xing J. Andersen R. A. (2000). Memory activity of LIP neurons for sequential eye movements simulated with neural networks. J. Neurophysiol. 84, 651–665 10938293 10.1152/jn.2000.84.2.651

[B172] Yan J. Scott T. R. (1996). The effect of satiety on responses of gustatory neurons in the amygdala of alert cynomolgus macaques. Brain Res. 740, 193–200 10.1016/S0006-8993(96)00864-5 8973814

[B173] Yantis S. (1992). Multielement visual tracking: attention and perceptual organization. Cogn. Psychol. 24, 295–340 10.1016/0010-0285(92)90010-Y 1516359

[B174] Yantis S. Schwarzbach J. Serences J. T. Carlson R. L. Steinmetz M. A. Pekar J. J. (2002). Transient neural activity in human parietal cortex during spatial attention shifts. Nat. Neurosci. 5, 995–1002 10.1038/nn921 12219097

[B175] Yarbus A. L. (1961). Eye movements during the examination of complicated objects. Biofizika 6, 52–56 14040367

[B176] Zipser D. Andersen R. A. (1988). A back-propagation programmed network that simulate response properties of a subset of posterior parietal neurons. Nature 331, 679–684 10.1038/331679a0 3344044

